# A combinatorial domain screening platform reveals epigenetic effector interactions for transcriptional perturbation

**DOI:** 10.1038/s41467-026-72227-9

**Published:** 2026-04-24

**Authors:** Hyungseok C. Moon, Michael H. Herschl, Alessandra Sclip, Vincent Q. Tran, April Pawluk, Silvana Konermann, Patrick D. Hsu

**Affiliations:** 1 Arc Institute, Palo Alto, CA USA; 2 University of California, Berkeley—University of California, San Francisco Graduate Program in Bioengineering, Berkeley, CA USA; 3 Department of Biochemistry, Stanford University, Stanford, CA USA; 4 Department of Bioengineering, University of California, Berkeley, Berkeley, CA USA; 5 Center for Computational Biology, University of California, Berkeley, Berkeley, CA USA

**Keywords:** High-throughput screening, Functional genomics, Epigenetics, Transcription, CRISPR-Cas systems

## Abstract

Epigenetic regulation involves the coordinated interplay of diverse proteins. To systematically explore these combinations, we present COMBINE (combinatorial interaction exploration), a high-throughput platform that tests over 50,000 pairs of epigenetic effector domains up to 2,094 amino acids in length for their ability to modulate endogenous human gene transcription. COMBINE reveals diverse synergistic interactions between epigenetic protein domains, including a potent KRAB-L3MBTL3 fusion that increases the effective targeting window, enhances gene silencing in dose-limited conditions, and enables robust dual-directional CRISPR perturbation. Inducible screening shows DNA methylation modifiers are essential for epigenetic memory, with distinct combinations driving long-term repression and activation. This systematic analysis of pairwise domain interactions advances our understanding of epigenetic crosstalks and the development of next-generation epigenome editing tools. More broadly, COMBINE offers a generalizable platform to functionally characterize combinatorial biological processes at scale.

## Abstract (web summary)

Epigenetic proteins cooperate to regulate gene expression, but testing combinations of large functional domains is challenging. Here, authors present COMBINE, a high throughput platform screening 50,000+ epigenetic domain pairs to identify combinations that potently and durably control gene expression.

## Introduction

Epigenetic regulation in mammalian cells is a highly coordinated process, essential for organizing genetic information and orchestrating pivotal cellular functions^1^ like gene regulation, differentiation, and DNA replication^2^ or repair^3^. Central to this regulation are large, multi-protein complexes that control epigenetic states through intricate combinations of post-translational modifications of DNA-scaffolding histone proteins and epigenetic modifications of the genome itself^4^. These complexes often contain numerous subunits with distinct functionalities^5,6^, such as the Polycomb repressive complexes^7^, that coordinate histone methylation and ubiquitination states to enable context-dependent control of gene expression and chromatin state.

Inspired by these natural mechanisms, the epigenome editing toolbox leverages similar combinatorial concepts that bring together programmable DNA-binding domains with epigenetic effector proteins^8,9^. For maximal activity, transcriptional activators such as the synergistic activation mediator (SAM) complex fuse a viral activation domain to dCas9 in addition to aptamer-based recruitment of two other activators^10^. Epigenetic silencers such as CRISPRoff or EvoETR have also benefited from combining DNA methyltransferase domains with Krüppel associated box (KRAB) repressors, enabling long-term gene silencing with hit-and-run delivery of the editor system^11–13^.

To date, the development of epigenetic editors has largely relied on rational design or low-throughput, guess-and-test approaches^10–23^. Recent advances in pooled gene synthesis have enabled the functional screening of short peptide libraries of ~80 amino acids tiling larger effector protein candidates^24–27^, further allowing the annotation of novel bivalent interactions that are synergistic or antagonistic^28^. However, the synthesis length restriction of current-generation oligonucleotide pools may not accommodate the size of functionally active elements, and the use of engineered reporter loci may not extrapolate to endogenous genetic contexts. One recent study used barcoded multicistronic adaptors to clone and screen pairs of transcription factors up to 5.8 kilobases in length using a T cell knock-in system, but the generalizability and scale of this approach is limited by the requirement of a cell-type specific functionally monoallelic target locus and a sharp length-dependent integration bias^29^.

To address these challenges, we develop a generalizable high-throughput screening platform to functionally characterize combinations of long and heterogeneous epigenetic effectors, with the goal of 1) identifying effector combinations that increase the potency or durability of transcriptional perturbations, and 2) uncovering previously unknown interactions between individual effector domains.

Here, we report COMBINE (combinatorial interaction exploration), a high-throughput and inducible screening platform that can accommodate heterogeneously-sized fusion elements, including large catalytic domains up to 6.3 kilobases in length. We apply COMBINE to measure the transcriptional perturbation landscape of an endogenous human gene with over 50,000 bivalent epigenetic effectors, composed of pairs of protein domains from diverse classes including readers, recruiters, structural factors, and catalytic writers or erasers.

Systematic analysis and validation of combinatorial interactions enables the discovery of previously unknown synergistic or antagonistic interdomain interactions that control epigenetic regulation of transcription in human cells. These results nominate epigenetic effector domain combinations for synthetic biology and genetic medicine, including a potent KRAB-L3MBTL3 fusion that increases the effective targeting window, enhances gene silencing in dose-limited conditions, and enables robust dual-directional CRISPR perturbations to simultaneously activate one target gene and silence another. Long-term analysis identifies CpG DNA methylation-dependent epigenetic memory with distinct expression profiles. Effector combinations drive heritable silencing, downregulation, or activation in a gene-specific manner, suggesting differential epigenetic susceptibility across genes. COMBINE is generalizable to a wide variety of lentivirus-transducible cell types and protein domain classes, as well as DNA and RNA elements, providing a broad platform to interrogate biological interactions at scale.

## Results

### Combinatorial interaction exploration (COMBINE) enables high-throughput bivalent domain screening

**Fig. 1:**
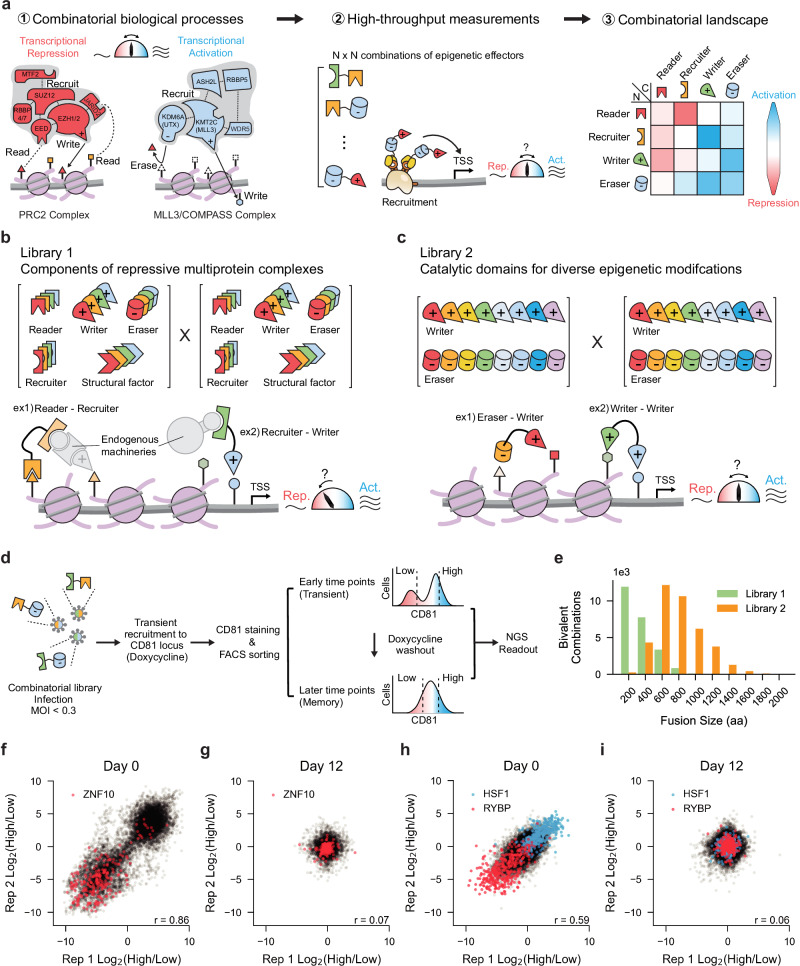
COMBINE discovers epigenetic effector pairs that regulate endogenous human transcription. **a** Conceptual framework for interrogating combinatorial biological processes through HTS of an N x N combinatorial domain library. Schematics of representative multi-protein complexes, including the Polycomb repressive complex 2 (PRC2) and the activating MLL3/COMPASS complex, are shown. N x N combinatorial domains are recruited to the endogenous target gene loci by a dCas9/MS2 system. Subpopulations of cells are enriched based on the target gene expression level in a high-throughput manner. Following the NGS readout, the combinatorial landscape of transcriptional perturbation is analyzed. **b** Schematic of Library 1 composed of epigenetic readers, recruiters, structural factors, and writers/erasers from diverse multi-protein complexes with established transcriptional repression activity. Two representative domain pairs are shown. A full list of members is available in Supplementary Data 1. **c** Schematic of Library 2 including direct writers and erasers of diverse epigenetic modifications, curated agnostic of their known effect on transcription. A full list of members is available in Supplementary Data 2. **d** Experimental procedure for the combinatorial HTS of epigenetic effector pairs. Combinatorial domain candidates are recruited to the endogenous *CD81* locus for 5-6 days with doxycycline. Cells are stained with anti-CD81 antibody and sorted into High and Low bins by expression level, followed by NGS to measure enrichment of bivalent domain candidates. Timepoints following doxycycline removal include Day 0, 6, and 12 (Library 1) and Day 0 and 12 (Library 2). For more detailed descriptions, refer to methods and Supplementary Figs. 5 and 6. **e** Theoretical size distribution of bivalent domain candidates from Library 1 and 2 assuming uniform coverage. **f**, **g** Scatter plots of enrichment scores from Library 1 at Day 0 (**f**) and Day 12 (**g**). Effectors containing the repressive *ZNF10* KRAB domain are highlighted in red. Pearson correlation coefficient (r) between replicates is shown. **h**, **i** Scatter plots of enrichment scores from Library 2 at Day 0 (**h**) and Day 12 (**i**). Effectors containing the repressive RYBP domain are highlighted in red; those containing the HSF1 activator are highlighted in blue. Pearson correlation coefficient (r) between replicates is shown. Source data are provided as a Source Data file.

Considering the combinatorial nature of epigenetic regulation (Fig. 1a), we sought to assess the functional effects of epigenetic domain pairs on gene expression to uncover new biology and potential domain combinations for epigenome editing tools. The limitations of currently available bivalent domain screening technologies necessitated the development of a new pairwise domain screening approach that could accommodate larger domain sizes (many over 1000 amino acids in length) to assess combinations of epigenetic reader, recruiter, writer, and eraser domains for their effects on gene expression in a high-throughput pooled screen.

To achieve this, we first curated two complementary libraries of epigenetic effectors, each with distinct design principles. Library 1 was designed to identify combinatorial epigenetic repressors and is composed of readers, recruiters, structural factors, writers, and erasers from known repressive epigenetic multiprotein complexes (Fig. 1b and Supplementary Data 1). It contains a total of 155 domains ranging from 80 aa to 545 aa (Supplementary Fig. 1a, b). Library 2 was designed to investigate how diverse types of catalytic epigenome modifiers interact to modulate transcription in both directions (repression or activation). This library is exclusively composed of catalytic writers and erasers of histone and DNA modifications, irrespective of their known effects on transcription (Fig. 1c and Supplementary Data 2). It contains 198 domains, with a wider size distribution from 100 aa to 1,036 aa (Supplementary Figs. 1c, d). To mitigate length bias in lentivirus generation, transduction^30^, and NGS readout^31^, we increased the relative proportion of larger library members during pooling and utilized sequential golden gate assembly with CcdB dropout selection to increase cloning efficiency (Methods, Supplementary Figs. 2 and 3).

To recruit these epigenetic effectors to an endogenous gene promoter, we used dCas9 targeting in combination with the MS2-MCP aptamer system derived from RNA bacteriophage MS2^32^, analogous to our prior designs that utilize sgRNAs with embedded MS2 stem-loops^10,33^. For temporal control over the editing complex, doxycycline-inducible promoters drove the expression of both dCas9 and the effector combination fused to monomeric MS2-coat protein (MCP) or synonymous mutated tandem MCP (stdMCP)^34^ (Supplementary Fig. 4a,e). To assess transcriptional outcomes on an endogenous target locus, we chose the gene encoding membrane protein CD81, whose expression can be measured using flow cytometry. We designed a three sgRNA expression cassette targeting upstream of the *CD81* transcription start site (TSS) and confirmed the ability to transiently activate and repress CD81 expression with this experimental setup. Using well-characterized effectors including KRAB and the transcriptional activation domain of HSF1, CD81 expression levels showed a good dynamic range of both repression and activation during doxycycline treatment, and returned to baseline by 9 days after the removal of doxycycline (Methods, Supplementary Fig. 4b-d,f-h).

For our screens, we transduced the lentiviral libraries encoding Library 1 or Library 2 into K562 cells containing Tet-On dCas9 at a low multiplicity of infection (MOI < 0.3) with a minimum effector coverage of 100X (Methods, Fig. 1d and Supplementary Figs. 5a and 6a). The combinatorial effector was recruited to the *CD81* locus for 5-6 days by adding doxycycline, followed by cell sorting based on CD81 expression (Day 0 timepoint to evaluate immediate effects on gene expression) (Methods, Supplementary Figs. 5b–d and 6b–d). This was followed by a 12-day effector washout period without doxycycline and a final round of cell sorting (Day 12 timepoint to evaluate durable effects on gene expression in the absence of effectors).

The size differences between Library 1 and Library 2 effector domain combinations (Fig. 1e) necessitated different NGS readout strategies. The majority (~82%) of bivalent members in Library 1 fell below the 1500 nucleotide upper limit for short-read NGS platforms^35^, enabling us to directly sequence the N- and C-termini of effector pairs using short-read sequencing (Supplementary Fig. 5a). In contrast, Library 2 often exceeded this limit. To overcome this, we included a short 20 N barcode in the cloning process, which was initially mapped to the effector pair by nanopore sequencing and read out by short-read sequencing at the end of the screen (Methods, Supplementary Figs. 3g–k and 6a, e). Importantly, the barcode mapping approach circumvents PCR amplification of combinatorial candidates through Cas9-mediated digestion of the plasmid pool for nanopore library preparation, thereby eliminating the risk of PCR-mediated template switching. Targeted nanopore sequencing of genome-integrated Library 2 members after lentiviral transduction indicated less than 10% barcode swapping (Supplementary Fig. 7). Barcoded approach with exponential effector pooling for Library 2 resulted in a reduction of the Day 0 dropout rate from 48.7% in Library 1 to 4.2% and successfully offset the exponential decay of effector coverage with increasing length that was observed for Library 1 and previous combinatorial screening approaches (Supplementary Fig. 8a, d)^29,36^. Effector dropout on Day 12 increased to 56.3% for Library 1 and 19.3% for Library 2 (Supplementary Fig. 8b, c, e).

The transcriptional effects of each domain combination were determined by measuring the relative abundance of each unique effector pair in the sorted populations from the high and low CD81 expression bins, calculated as log_2_ fold enrichment, across two screening replicates per library. Furthermore, the use of multiple barcodes per effector pair in Library 2 allowed statistical testing at the barcode level, with each barcode treated as an independent observation Supplementary Data 3. Both libraries effectively captured the temporal dynamics of *CD81* transcriptional perturbation induced by well-established control effectors. In Library 1, target gene repression by combinations containing the KRAB domain from ZNF10 was strong at Day 0 and disappeared by Day 12 (log_2_ fold score of −4.8 and −0.27, Fig. 1f, g and Supplementary Data 3). Similarly, repression by full-length RYBP, a known epigenetic silencer that recruits Polycomb repressive complex 1 (PRC1)^37^, and activation by the activation domain of HSF1 were captured at Day 0 and dissipated by Day 12 after doxycycline removal for Library 2 (average log_2_ fold scores of −3.6 and 0.083 for RYBP and 1.9 and 0.047 for HSF1, Fig. 1h, i and Supplementary Data 4). Based on CD81 expression levels measured by flow cytometry and the drop in both magnitude and correlation of HTS enrichment scores (r = 0.59-0.86 on Day 0 to r = 0.06-0.07 at Day 12; Supplementary Fig. 5c and Supplementary Data 3 and 4), the majority of transcriptional effects disappeared by 12 days after the removal of doxycycline (Day 12), highlighting the transient nature of perturbations introduced by the majority of epigenetic and transcriptional modifiers.

### COMBINE measures combinatorial landscapes of pairwise epigenetic interactions

**Fig. 2:**
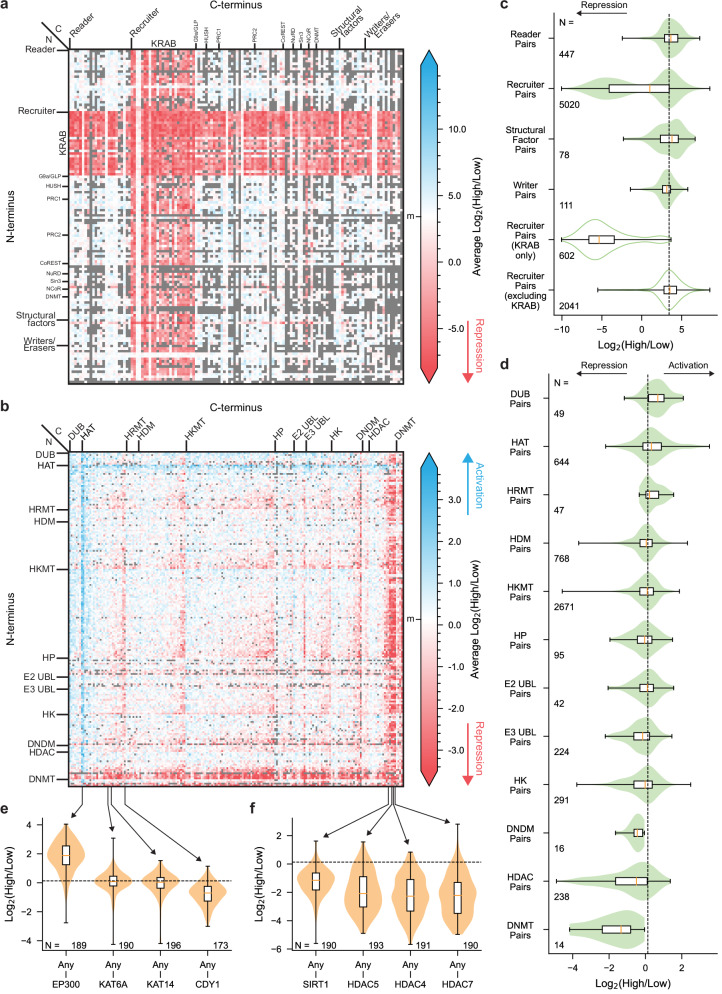
Epigenetic pairs generate a rich combinatorial landscape of transient gene expression outcomes. **a** Heatmap of enrichment scores generated from Library 1 at Day 0. Average Log_2_(High/Low) enrichment scores across both replicates are plotted. High enrichment scores do not necessarily indicate active upregulation of CD81 expression, owing to the relative nature of the FACS gating strategy (Supplementary Fig. 5) and repressor-focused composition of Library 1. Color scale is centered at the mode of the enrichment score distribution, indicated by the “m” tick. The maximum or minimum color saturation is determined by the 1st or 99th percentile, depending on which is farther from the mode, and the scale is symmetrically extrapolated to the same distance on the opposite side. For this repressor-focused library, lower Log_2_ scores indicate stronger repression. Dropouts are indicated in gray. **b** Heatmap of enrichment scores generated from Library 2 at Day 0. Average Log_2_(High/Low) enrichment scores across both replicates are plotted. Color scale conventions as in (**a**). For this library composed of putative repressors and activators, lower scores indicate more repression and higher scores indicate more activation. Dropouts are indicated in gray. Histone deubiquitinase (DUB), histone acetyltransferase (HAT), histone arginine methyltransferase (HRMT), histone demethylase (HDM), histone lysine methyltransferase (HKMT), histone phosphatase (HP), E2 ubiquitin ligases (E2 UBL), E3 ubiquitin ligases (E3 UBL), histone kinase (HK), DNA demethylation machinery (DNDM), histone deacetylase (HDAC), and DNA methyltransferase (DNMT). **c** Violin plots of all possible bivalent combinations within the same classes from Library 1. A breakdown of the recruiter class into KRAB and non-KRAB pairs is also shown (not shaded). For all violin plots (**c**–**f**): orange bar = median, box spans Q1–Q3, whiskers extend to min/max, dashed line indicates distribution mode of all pairs, and n indicates number of combinations. **d** Violin plots of all possible bivalent combinations within the same classes from Library 2. **e** Violin plots of all possible bivalent combinations with the specified HAT effectors on the C-terminus. **f** Violin plots of all possible bivalent combinations with the specified HDAC effectors on the C-terminus.

To visualize transient perturbation outcomes across the large space of combinations tested, we generated enrichment score heatmaps (Fig. 2a, b and Supplementary Fig. 9). We observed clear diagonal symmetry between the X-Y and Y-X orientations of the same two effectors, with a high degree of correlation across pairs, indicating that most effector pairs behaved similarly regardless of orientation (Library: 1 r = 0.73; Library 2: r = 0.69, Supplementary Fig. 10).

To identify general trends for the transcriptional impact of effector classes, we grouped pairs based on the families of individual effectors (Fig. 2c, d). While Library 1 revealed the strongest repressive phenotypes among KRAB family pairs, Library 2 revealed strong repression across pairs of histone deacetylases (HDACs) and DNA methyltransferases (DNMTs). We also observed a surprising trend toward mild target gene repression with pairs of DNA demethylation machinery (DNDM) domains. Pairs of histone acetyltransferases (HATs) tended to activate gene expression, as did histone deubiquitinases (DUBs) despite their ability to remove both activating and repressive histone ubiquitination marks. Finally, pairs of histone lysine methyltransferases (HKMTs) and histone kinases (HKs) ranged broadly across both activation and repression outcomes.

Next, we examined the directionality of individual effectors in the context of these class-level trends. In the HAT class, pairs containing C-terminal EP300 (a broad HAT capable of acetylating all four core histones^38^) tended to activate transcription, whereas combinations containing C-terminal CDY1 (which displays a strong acetylation preference for histone H4^39^) tended to repress transcription (Fig. 2e). In addition, some HAT effectors, such as KAT6A or KAT14 (a broad histone and weak H4 acetyltransferase, respectively^40,41^), exhibited more partner-dependent effects.

Compared to the HAT class, the HDAC domain class had more members that consistently repressed transcription irrespective of their N-terminal partners such as SIRT1 (a broad histone deacetylase and decrotonylase^42^) and class IIa HDACs (HDAC5, HDAC4, and HDAC7, Fig. 2f). However, the degree of repression ranged widely depending on the exact effector pairing. For example, the combination of EP300 and HDAC5 activated transcription with an average log_2_ fold enrichment of 1.1, while the pairing of UBE2E1 and HDAC5 repressed transcription with a score of −4.5. These global observations motivated us to quantitatively analyze the contribution of individual domain effects and domain-domain interactions to gene expression outcomes.

### Marginal effector analysis reveals how individual domains affect transcription

**Fig. 3:**
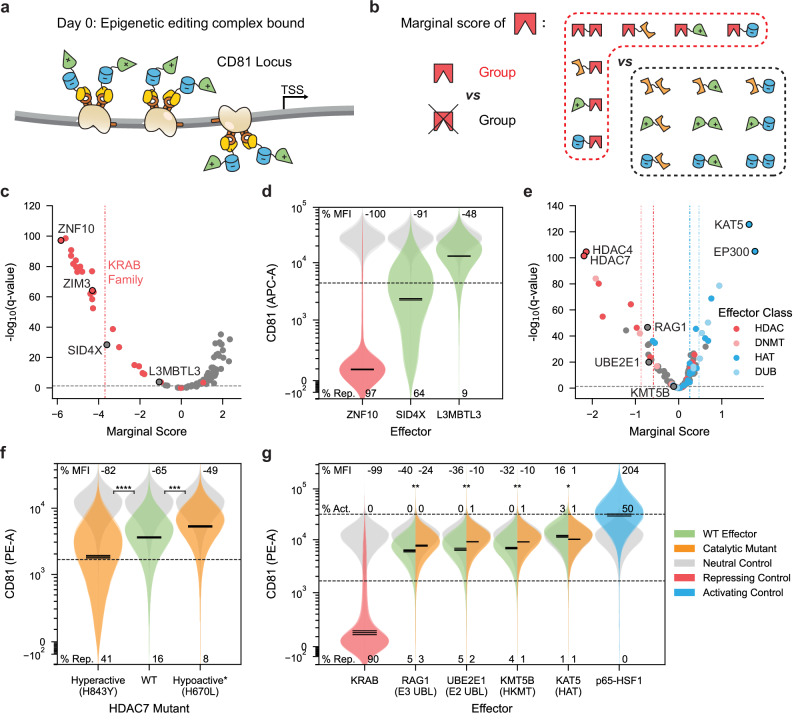
Marginal effector analysis reveals how individual domains perturb transcription across partners. **a** Schematic of the dCas9/MS2 epigenetic editing complex bound at the *CD81* locus after doxycycline addition for 5-6 days. **b** Marginal effector analysis: for a given effector, the marginal score is the mean Log_2_(High/Low) difference between pairs with and without the effector. Q-values are FDR-corrected p-values from two-tailed Welch’s t-test. **c** Volcano plot of marginal scores from Library 1 at Day 0. KRAB family members are highlighted in red. Vertical and horizontal dashed lines indicate the average KRAB marginal score and q-value of 0.05, respectively. **d** Violin plots of CD81 expression 3 days post nucleofection of Library 1 effector plasmids. Mock transfection (pUC19) in gray. Dashed line indicates repression gate at the 1st percentile of pUC19. Average percentages of repressed cells and CD81 MFI changes versus pUC19 are indicated. **e** Volcano plot of marginal scores from Library 2 at Day 0. Effectors from HDAC, DNMT, HAT, and DUB classes are highlighted in red, pink, blue, and sky blue, respectively. Vertical dashed lines indicate average marginal score for each respective colored class. Horizontal dashed line indicates q-value of 0.05. **f** Violin plots of CD81 expression after expression of HDAC7 deacetylase domain and its mutants. Dashed line indicates repression gate at the 1st percentile of the neutral condition. *proposed mutant inferred from HDAC4. **g** Violin plots of CD81 expression after expression of individual effectors from Library 2. Dashed lines indicate repression and activation gates at the 1st and 99th percentile of neutral condition. For violin plots (**d**), (**f**), and (**g**): Color coding: WT = green, mutants = orange, neutral = gray, repressing control = red, activating control = blue. **f**, **g** are 5 days post nucleofection and doxycycline induction. 2 (**d**) or 3 (**f,g**) replicates shown as translucent overlays. Geometric means of each replicate are shown as solid black lines. Average percentages of repressed/activated cells and CD81 MFI changes versus DMD are indicated for (**f**) and (**g**). Geometric means from WT versus mutant effectors were tested by two-tailed Welch’s t-test: *p ≤ 0.05, **p ≤ 0.01, ***p ≤ 0.001, ****p ≤ 0.0001, ns not significant. Source data are provided as a Source Data file.

To quantitatively assess how each of the individual effector domains in our two libraries contribute to epigenetic control of gene expression, we calculated a ‘marginal score’ for each domain (Fig. 3a, b). This score reflects the average enrichment value across all bivalent combinations containing the domain of interest relative to all combinations lacking it, yielding a measurement of each domain’s relative contribution to target gene expression (positive or negative) across pairings.

Marginal effector analysis of Library 1 clearly reflected the well-known repressive effect of KRAB domains on gene expression^16,24^. The average marginal score across the KRAB domain class was −3.7, indicating an average 13-fold decrease in enrichment scores when KRAB is present in an effector pair (2^3.7^ ≈ 13, Fig. 3c and Supplementary Data 3). The ZNF10 (KOX1) KRAB domain – which is commonly used in CRISPR interference (CRISPRi) applications^43^ – exhibited the lowest marginal score at −5.8. The more recently identified ZIM3 KRAB domain^20^ also exhibited strong repression with a score of −4.3. Notably, 23 out of 25 tested KRAB domains repressed gene expression, consistent with the well-established repressive capability of the KRAB domain family^24^.

Outside the KRAB domain family, we identified two other individual domains with significantly repressive marginal scores in Library 1: SID4X (marginal score −3.6) and the sterile alpha motif (SAM) domain of L3MBTL3 (marginal score −1.1). SID4X is an engineered ternary version of the Sin3 interacting domain (SID) from the MAX dimerization protein 1 (MXD1) that efficiently recruits the Sin3-HDAC complex^44^, and the SAM domain of L3MBTL3 is known to multimerize and recruit lysine-specific histone demethylase 1 A (LSD1/KDM1A)^45,46^, providing mechanistic hypotheses for the repressive effects of each of these domains.

To independently validate each domain’s individual contribution to transcriptional repression outside of the bivalent effector context, we fused ZNF10 KRAB, SID4X, or L3MBTL3 SAM to stdMCP and expressed them in the Tet-On dCas9 K562 cell line with a preinstalled *CD81*-targeting guide array (Fig. 3d). Repression of *CD81* target gene expression, as measured by the change in total mean fluorescence intensity (MFI), correlated with each domain’s relative marginal effect score: ZNF10 KRAB decreased CD81 MFI by 100%, SID4X by 91%, and L3MBTL3 by 48% relative to the negative control. Notably, when over-expressed in this experimental system, KRAB resulted in near-complete silencing of *CD81*, while SID4X and L3MBTL3 produced more graded, intermediate levels of repression. Repression by these effectors was targeted, as effector expression in a Tet-On dCas9 K562 cell line alongside a non-targeting guide array did not repress *CD81* (Supplementary Fig. 11a).

The marginal scores calculated from Library 2 were lower in magnitude, likely reflecting the more varied composition of this library in comparison to the repression-focused Library 1 (Fig. 3e and Supplementary Data 4). At the class level, the DNMT class exhibited the highest average repression, with a moderate effect size but high consistency across members (average marginal score −0.87). The DUB class resulted in the highest average gene activation with moderate but consistent effects (average marginal score 0.47). At the individual effector level, five of the seven top repressive domains in Library 2 belonged to the HDAC class (including HDAC7 and HDAC4 with scores of −2.2 and −2.1, respectively), while the HAT class contained the two strongest individual activators (EP300 and KAT5 with scores of 1.8 and 1.6, respectively).

Next, we confirmed the effects of 5 individual domains by fusing them to MCP and transiently expressing them in the same cell line used above. The strongest repressor, HDAC7 (marginal score −2.2), reduced CD81 MFI the most, by 65%, and was modulated by the introduction of point mutations (Fig. 3f). The hyperactive H843Y mutant^47^ increased repression to 82%, and the hypoactive H670L mutant^48^ diminished repression to 49%, both while maintaining similar expression levels when fused to mCherry (Methods, Supplementary Fig. 12c, d, g and Supplementary Data 5). Three additional repressors from diverse classes also repressed *CD81* in concordance with their marginal scores. RAG1 (marginal score −0.72), an E3 ubiquitin ligase (E3 UBL) associated with histone H3 monoubiquitination during V(D)J recombination^49,50^, repressed CD81 by 40%; UBE2E1 (marginal score −0.69), an E2 ubiquitin ligase (E2 UBL) associated with PRC1^51^, repressed CD81 by 36%; and KMT5B (marginal score −0.12), a histone lysine methyltransferase (HKMT) that writes H3K20 methylation^52^, repressed CD81 by 32% (Fig. 3g). Catalytic mutations of these three enzymes^53–55^ minimally affected expression yet reduced *CD81* repression to 24%, 10%, and 10% respectively (Supplementary Fig. 12d, g). However, *CD81* repression by RAG1 and KMT5B was guide-independent (Supplementary Fig. 11b), indicating that non-targeted effects on gene expression can also contribute to our screening results.

For activation, KAT5 (marginal score 1.6) was the second-ranking activator of gene expression in the screen, and validation experiments confirmed that it resulted in a guide-dependent 16% increase in CD81 MFI (Fig. 3g and Supplementary Fig. 11b), which was nearly abolished upon introduction of a catalytic point mutation that did not significantly impact expression (Supplementary Fig. 12d). Altogether, marginal effector analysis and experimental validation identified diverse functional protein domains with transcriptional effects spanning silencing, downregulation, and activation.

### COMBINE reveals synergy and antagonism in epigenetic domain interactions

**Fig. 4:**
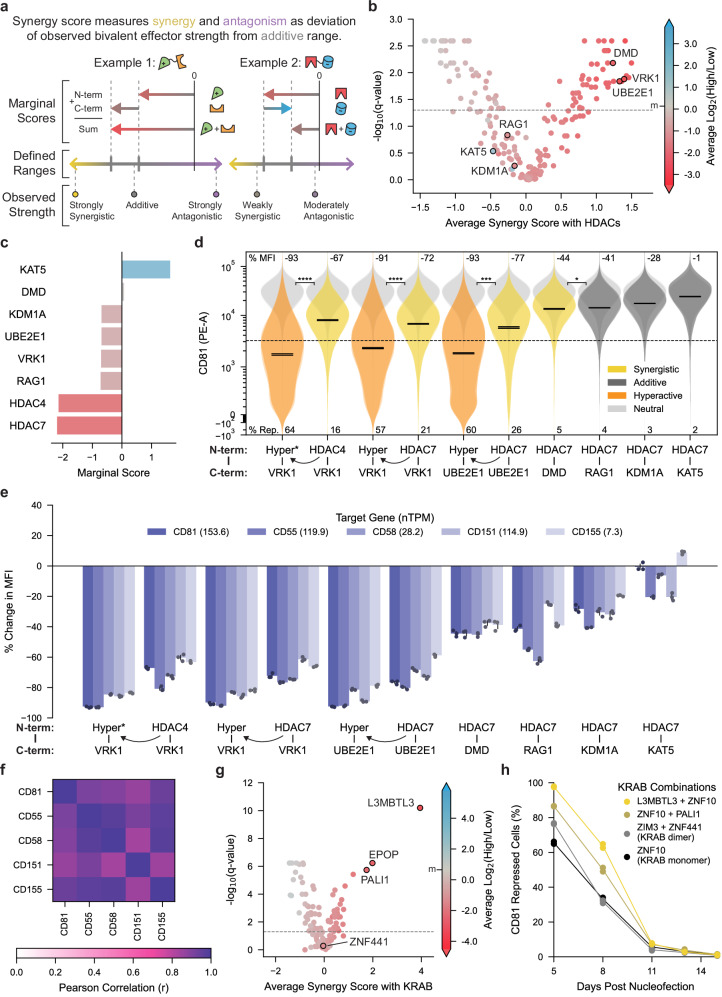
COMBINE uncovers diverse modes of epigenetic interactions. **a** Concept of synergy score calculation. For each combination, an additive range is calculated from individual effector marginal scores. Synergy scores quantify deviation from the additive range, with positive and negative values indicating synergy and antagonism (**Methods detail**). **b** Volcano plot of average synergy scores with HDAC family from Library 2 on Day 0. Effectors are colored by average Log_2_(High/Low) across all combinations containing the given effector with significant repressors of the respective family (Supplementary Fig. 17a). *Q* values are FDR-corrected *p* values from a two-sided one-sample Wilcoxon test. Horizontal dashed line indicates q-value of 0.05. **c** Marginal scores of individual domains constituting selected HDAC combinations. **d** Violin plots of CD81 expression 5 days after nucleofection and induction of HDAC combinations. Color coding: synergistic = yellow, additive = dark gray, hyperactive = orange, neutral = gray. Dashed line indicates repression gate at the 1st percentile of neutral condition. Average percentages of repressed cells and CD81 MFI changes versus DMD are indicated. *proposed mutant inferred from HDAC7. Arrows indicate WT to mutant form. 3 replicates shown as translucent overlays; geometric means as solid black lines and tested by two-tailed Welch’s t-test: *p ≤ 0.05, **p ≤ 0.01, ***p ≤ 0.001, ****p ≤ 0.0001, ns not significant. **e** Percent change in MFI relative to DMD control following dual nucleofection of plasmids encoding key HDAC combinations from Library 2 and 3X guide arrays targeting *CD55*, *CD58*, *CD151*, and *CD155*. *CD81* data are from single effector plasmid nucleofection into Tet-On dCas9 K562 (with 3X *CD81* guide array). Error bars represent the mean ± standard deviation of 3 replicates. *proposed mutant inferred from HDAC7. **f** Heatmap of Pearson correlations of percent change in MFI versus DMD control between each gene tested. Datasets consist of the 20 effectors shown in (**e**) and Supplementary Fig. 20a. **g** Volcano plot of average synergy scores with KRAB family from Library 1 on Day 6. Effectors colored and *Q* values calculated as in (**b**) (Supplementary Fig. 17b). **h** Timecourse of *CD81* repression following nucleofection of KRAB combination plasmids. Effector-expressing cells were sorted at day 2 and passaged to allow plasmid dilution (*n* = 2). Source data are provided as a Source Data file.

Beyond the effects of individual domains on target gene expression, the combinatorial design of COMBINE allows quantification of synergistic or antagonistic interactions among domain pairs. To systematically assess domain interactions, we calculated a ‘synergy score’ for each effector pair that uses individual marginal scores to calculate the difference between expected and observed effector strength for each domain combination (Methods, Fig. 4a). Positive scores indicate synergy and negative scores indicate antagonism in the direction of the stronger marginal effector. We also calculated an alternative residual-based synergy metric using measured homotypic pair enrichments to define expected effects (Supplementary Fig. 13**)**. While most pairs (~98%) showed good agreement between the two metrics, a small subset (~2%) exhibited strong anti-correlation due to sign adjustment artifacts when expected values were near zero (Supplementary Fig. 13d–f and Supplementary Note 1). We flagged these pairs, which show opposite signs despite similar synergy magnitudes (Supplementary Data 4). Across both libraries, we identified a diverse range of interactions between domains (Supplementary Figs. 14 and 15 and Supplementary Data 6 and 7). Compellingly, our analysis highlighted synergistic interactions between several PRC1 recruiters and a reader of H2AK119 monoubiquitination, recapitulating the known reader-writer synergy of variant PRC1 (Supplementary Fig. 16 and Supplementary Note 2)^37^.

We were intrigued by the broad interaction patterns observed across the robust repressive domain classes, KRAB and HDAC. When analyzing the synergy scores for all KRAB and HDAC members with clear individual repressive effects (marginal scores below −0.5, Supplementary Fig. 17), we were able to classify additive, synergistic, and antagonistic effector pairings with other library members (Fig. 4b and Supplementary Data 7). In total, 46 effectors resulted in synergistic enhancement of repression by HDAC domains. Of these effectors, we selected DMD, UBE2E1, and VRK1 with average synergy scores of 1.2, 1.3, and 1.4 and paired them with HDAC4 or HDAC7. We also experimentally validated three nonsynergistic effectors: the activator KAT5 as well as KDM1A and RAG1, which have similar marginal scores to UBE2E1 and VRK1 (Fig. 4c).

In validation experiments, we found that synergistic HDAC combinations consistently outperformed additive combinations, with UBE2E1 and VRK1 containing combinations reducing CD81 MFI by 67% to 77% relative to the neutral control (Fig. 4d). Repression by these combinations was guide-dependent and further enhanced to greater than 90% by incorporating hyperactive mutations into the HDAC partner (Supplementary Fig. 11c)^47^. Hypoactive or loss-of-function mutations^48,54,56^ in either or both of the two domains dramatically reduced but did not completely eliminate *CD81* repression (Supplementary Fig. 18a, b). Further, HDAC7 paired with neutral DMD control repressed CD81 more than combinations containing repressors RAG1 or KDM1A (−44% versus −41% and −28%, Fig. 4d). HDAC7 + DMD was expressed more highly than most combinations, potentially explaining the synergistic effect (Supplementary Fig. 12e). As the main activator-repressor pair tested, KAT5 canceled out repression by HDAC7 without reducing HDAC7 expression levels (Fig. 4d and Supplementary Fig. 12e).

To address the generalizability of HDAC combinations as well as individual domains identified from Library 2, we tested 20 key effectors on a panel of 4 additional basally expressed genes across a range of expression levels: *CD55*, *CD58*, *CD151*, and *CD155* (119.9, 28.2, 114.9, and 7.3 normalized transcripts per million, respectively^57^). Five days post-nucleofection of both effector and 3X guide array plasmids, we observed that the percent changes in MFI for each of the effectors were similar across all tested genes (Fig. 4e and Supplementary Figs. 18, 19 and 20a). The percent changes were highly correlated between genes, spanning a range of r = 0.84-0.98, demonstrating that identified HDAC combinations are broadly applicable to other target genes (Fig. 4f and Supplementary Fig. 20b).

Next, we examined synergy scores among the KRAB family of repressive domains to discern partner domains that could enhance or antagonize KRAB activity. Synergy score analysis identified the SAM domain from L3MBTL3 and two of the 27 PRC-related domains (PALI1 and EPOP) as the most potent synergistic partners of KRAB domains. All exhibited both high average synergy scores and low enrichment scores on Day 6 (average synergy scores 3.9, 1.7, and 2.0 and log_2_ fold enrichments −5.7, −3.4, and −3.7, respectively) (Fig. 4g, Supplementary Fig. 14b and Supplementary Data 6). Interestingly, PALI1 and EPOP are both accessory subunits specific to the PRC2.1 complex^58–60^. In individual validation of these domain combinations, all three KRAB combinations – L3MBTL3, PALI1, and EPOP – further enhanced repression of *CD81* compared to the ZNF10 KRAB domain alone (L3MBTL3 + KRAB, KRAB + PALI1, KRAB were 97.7%, 86.6%, and 65.6% of CD81-repressed cells, Fig. 4h and Supplementary Fig. 21a,b). Expression testing showed that synergy between KRAB and L3MBTL3 was not due to increased expression, as the combination was expressed at only half the level of KRAB alone (Supplementary Fig. 12a, b). Overall, COMBINE identified diverse interactions between epigenetic domain combinations—including synergistic, antagonistic, and additive effects—discovering combinations while validating known relationships as well.

### KRAB + L3MBTL3 combination enables potent transcriptional repression in challenging conditions

Individual KRAB domains, including those of ZNF10 and ZIM3, have been harnessed as epigenetic repressors in many contexts^20,43^ and yielded strong individual marginal scores in our screen (−5.8 and −4.3, respectively). However, further enhancement of repressive activity would be beneficial in circumstances where optimal repression is difficult to achieve. These conditions may arise due to limited delivery efficacy in therapeutic applications, target site constraints (e.g., allele-specific targeting) that limit optimal sgRNA selection, or when recruiting effectors to a locus indirectly (e.g., via an aptamer). To provide tool options for these challenging conditions, we examined the screen data for synergistic combinations that would enhance the potency of KRAB domains. In the screen data as well as in individual validation, the synergistic pairing of the KRAB domain with the SAM domain from L3MBTL3 was the most potent combination identified. Notably, the SAM domain is known for its ability to multimerize^45^, potentially recruiting multiple KRAB domains to the target gene.

**Fig. 5:**
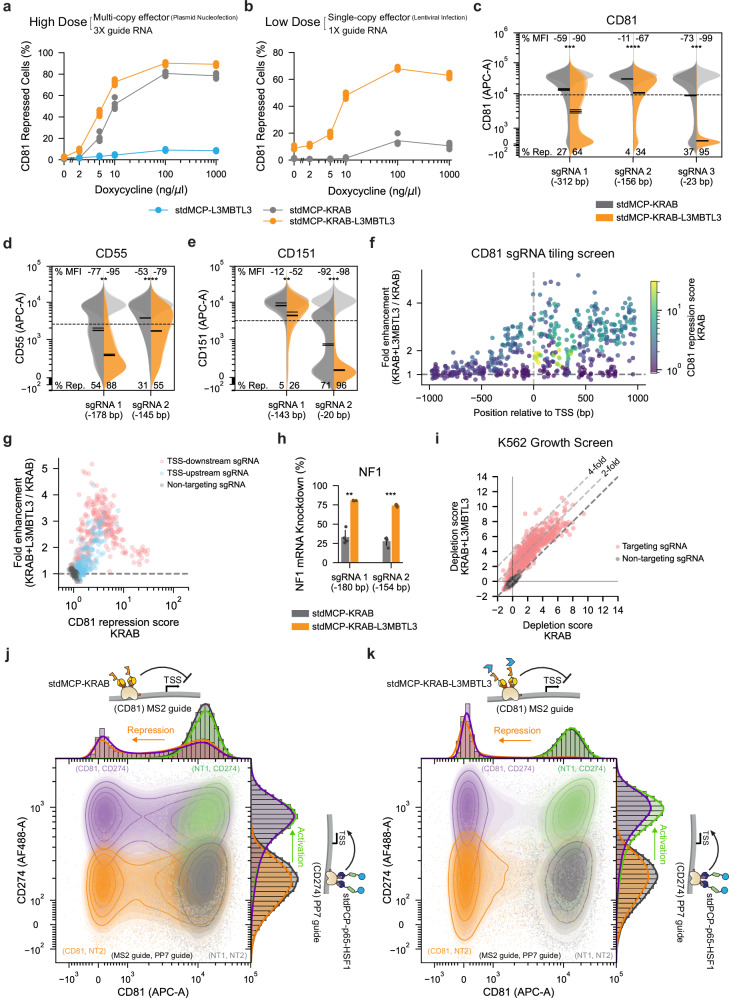
KRAB and L3MBTL3 combination enables potent transcriptional repression in challenging conditions. For **a**, **b** Dose-response curves of *CD81* repression. The KRAB domain is from *ZNF10* throughout this figure. **a** TRE3G-driven effector plasmids and 3X *CD81* guide array plasmid were nucleofected into Tet-On dCas9 K562 cells (*n* = 4, 3 days post nucleofection and doxycycline induction). **b** Stable K562 lines expressing TRE3G-driven effector were generated by sequential lentiviral transductions (**Methods detail**) (*n* = 4, 4 days post doxycycline induction). For (**c**-**e**): Violin plots of CD81 (**c**), CD55 (**d**), and CD151 (**e**) expression 5 days following lentiviral transduction with 1X sgRNA and doxycycline induction (1-2 μg/mL) to stable K562 lines expressing stdMCP-KRAB or stdMCP-KRAB-L3MBTL3. Color scheme: KRAB with on-target sgRNA (dark grey), KRAB + L3MBTL3 with on-target sgRNA (orange), KRAB with non-target sgRNA (light grey). sgRNA distance to TSS in parentheses. 3 replicates as translucent overlays; geometric means as solid black lines. Dashed lines indicate repression gate at bottom 1% of KRAB non-target condition. Average percentages of repressed cells and MFI changes relative to KRAB non-target control are indicated. Geometric means from KRAB versus KRAB + L3MBTL3 tested by two-tailed Welch’s t-test: *p ≤ 0.05, **p ≤ 0.01, ***p ≤ 0.001, ****p ≤ 0.0001. For (**f**,**g**): Fold enhancement of *CD81* repression (KRAB + L3MBTL3 / KRAB alone) from *CD81* sgRNA tiling screen. **f** Plotted against sgRNA position relative to TSS; color indicates KRAB repression score. **g** Plotted as a function of KRAB repression score; sgRNAs colored by position: TSS-upstream (blue), TSS-downstream (red), non-targeting (grey). **h** *NF1* mRNA knockdown measured by qRT-PCR 2 days after nucleofection of 1X sgRNA plasmid to stable K562 lines (*n* = 4). Error bars = mean ± SD. Color scheme, sgRNA distance to TSS, and statistical testing as in (**c**–**e**). (**i**) Scatter plot of depletion scores from CRISPRi growth screen comparing KRAB and KRAB + L3MBTL3 (*n* = 2 each). Targeting sgRNAs in red, non-targeting in grey. Dashed lines indicate equal depletion (y = x), 2-fold, and 4-fold enhancement. **j**,**k** 2D-contour plot and kernel density estimate (KDE) plots of populations transduced with dual MS2/PP7 sgRNAs, using (**j**) stdMCP-KRAB or (**k**) stdMCP-KRAB-L3MBTL3 for *CD81* repression and stdPCP-p65-HSF1 for *CD274* activation. Parenthetical genes indicate MS2 and PP7 sgRNA targets, respectively. Source data are provided as a Source Data file.

We tested the dose-dependent repressive capabilities of KRAB, L3MBTL3, and KRAB + L3MBTL3 by nucleofecting stdMCP fused effector plasmids with inducible promoters into our Tet-On dCas9 K562 cell line containing a 3X *CD81* guide array (Fig. 5a). The KRAB + L3MBTL3 combination was more potent than either effector alone across a broad range of doxycycline conditions. This difference was more pronounced under dose-limiting conditions: when effectors were delivered at low MOI via lentiviral transduction for single-copy insertion, and when targeting *CD81* with a single sgRNA also delivered by lentivirus rather than three (Fig. 5b). For example, at the 10 ng/μL doxycycline condition, KRAB domain alone repressed *CD81* in 1.4% of cells whereas KRAB + L3MBTL3 combination repressed *CD81* in 48.1% of cells.

To test the generalizability of KRAB + L3MBTL3 synergy, we first tested different guide RNAs targeting *CD81* gene and two additional gene targets (*CD55* and *CD151*) encoding cell surface proteins with basal expression in K562 cells. The KRAB + L3MBTL3 effector consistently demonstrated stronger repression across additional guide RNAs and target genes compared to KRAB alone (Fig. 5c–e), ranging from 1.1-fold repression in MFI with the highly effective *CD151* targeting sgRNA to 6.1-fold for a weaker *CD81* targeting sgRNA. Until now, individual validation experiments were performed with sgRNAs targeting upstream of the TSS. Next, we tested whether the KRAB + L3MBTL3 combination could enhance target gene repression with canonical CRISPRi sgRNAs targeting downstream of the TSS. Interestingly, with TSS-downstream sgRNAs from a previous study^61^, even dCas9 only condition without KRAB effectors was capable of repressing the target gene expression presumably through physical blockade of RNA polymerase II (Supplementary Fig. 22). Addition of the KRAB domain generated robust enhancement. Further addition of the L3MBTL3 SAM domain enhanced repression, though the effect size was moderate as KRAB alone already achieved substantial knockdown. To systematically examine how sgRNA position affects the enhancement by L3MBTL3, we performed a sgRNA tiling screen spanning ±1000 bp around the TSS of three genes: *CD55*, *CD81*, and *CD151* (Fig. 5f, Supplementary Fig. 23, and Supplementary Data 8). Highly potent sgRNAs showed a ceiling effect, with minimal additional enhancement by L3MBTL3 (Fig. 5g and Supplementary Fig. 23). In contrast, sgRNAs with moderate potency showed the greatest fold enhancement, demonstrating that L3MBTL3 can broaden the targeting window for effective gene repression. This may be particularly useful in clinical and other specialized applications where constraints such as allele-specific targeting requirements restrict sgRNA positioning to suboptimal locations. The KRAB + L3MBTL3 synergy extended beyond the ZNF10 KRAB domain to recently characterized KRAB domains from ZIM3 and ZNF705^20,27^(Supplementary Fig. 24).

We further compared by targeting neurofibromin (*NF1*), whose expression is driven by the core promoter with initiator element (INR), a distinct promoter type not present in the tested CD genes. Similar to our previous observations, the KRAB + L3MBTL3 fusion exhibited significantly enhanced repression compared to KRAB alone (Fig. 5h). To test additional target genes at scale, we conducted a CRISPRi growth screen with a sgRNA library targeting promoters of 57 genes with varying essentiality in K562 cells. For each target gene, we designed 20 sgRNAs targeting the promoter region, both upstream and downstream of the TSS. RT-qPCR validation of 5 sgRNAs targeting AQR, an essential gene included in the screen, confirmed screen results, showing enhanced repression with KRAB + L3MBTL3 and concordance between depletion scores and mRNA levels (Supplementary Fig. 23). KRAB + L3MBTL3 broadly enhanced sgRNA depletion across the screen: at the sgRNA level, 918/1140 (80.5%) targeting sgRNAs had significantly stronger depletion scores with KRAB + L3MBTL3 (difference in depletion score between KRAB + L3MBTL3 and KRAB > 2 SD of non-targeting controls), providing ~1.8X greater effect sizes overall (Fig. 5i). At the gene level, 54/57 genes (94.7%) showed significant enhancement in depletion (two-sided Wilcoxon signed-rank test, FDR < 0.05) (Supplementary Data 8). Moreover, an internally normalized competitive growth assay suggested the expression of KRAB + L3MBTL3 combination does not affect cell viability^61^ (Supplementary Fig. 21c). To validate the KRAB + L3MBTL3 combination beyond the MS2-based indirect recruitment system and K562 cell line, we tested direct dCas9 fusions in NGN2-induced neurons from H1 human embryonic stem cells (Supplementary Fig. 25). The L3MBTL3-dCas9-ZNF10 KRAB construct showed enhanced repression compared to dCas9-ZNF10 KRAB alone, though the improvement was more modest than in the MS2 system, likely because the CAG promoter-driven dCas9-ZNF10 KRAB fusion already achieved strong baseline repression.

We reasoned that the increased efficacy afforded by the KRAB + L3MBTL3 combination could be particularly enabling for dual-directional CRISPR perturbations, in which two orthogonal epigenetic effectors can be targeted to two distinct loci to achieve upregulation of one gene and simultaneous repression of another^10,62,63^. To assess this possibility, we used the stdMCP fusion strategy to recruit KRAB alone or KRAB + L3MBTL3 to an MS2-containing *CD81* sgRNA or non-targeting control guide, while using an analogous setup to recruit the p65-HSF1 CRISPRa effector^64^ using the orthogonal stdPCP-PP7 system^34,62^. We engineered cell lines to express dCas9, stdMCP-KRAB or stdMCP-KRAB-L3MBTL3, and stdPCP-p65-HSF1 in a doxycycline-inducible manner and introduced four dual MS2/PP7 sgRNA combinations – each containing either a *CD81*-targeting or a non-targeting MS2 sgRNA paired with a *CD274*-targeting or a non-targeting PP7 sgRNA – using lentivirus.

Importantly, we found that the KRAB + L3MBTL3 combination fully silenced CD81 expression to produce four distinct populations, unlike the KRAB-only condition (Fig. 5j, k). This improved separation resulted from augmented repression by the KRAB + L3MBTL3 combination (KRAB: 41.5% vs KRAB + L3MBTL3: 89.4% *CD81* repressed cells) without interfering with *CD274* activation by the PP7 system (KRAB: 66.5% vs KRAB + L3MBTL3: 74.5% *CD274* activated cells) (Supplementary Fig. 26). The KRAB + L3MBTL3 combination therefore enabled us to overcome limitations in the efficacy of MS2-based repressors and develop a robust MS2/PP7-based dual-directional perturbation system, which may be applied to future dual-directional CRISPR screening^65,66^ or therapeutic applications.

### Long-term epigenetic memory requires modifiers of DNA methylation

**Fig. 6:**
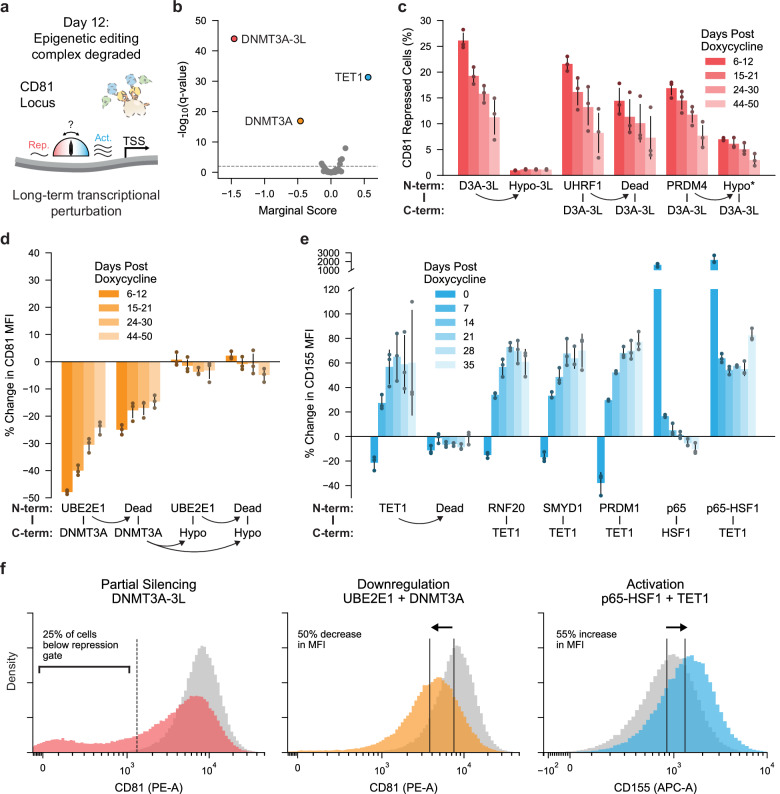
DNA methylation writers and erasers are essential for the memory of silencing, repression, and activation of gene expression. **a** Schematic showing loss of the dCas9/MS2 epigenetic editing complex 12 days post-withdrawal of doxycycline. *CD81* expression perturbations rely on sustained epigenetic modifications. **b** Volcano plot of marginal scores from Library 2 on Day 12. Horizontal dashed line indicates *q* value of 0.05. For (**c**, **d**) Day 0 indicates the day of doxycycline washout. Plasmids were nucleofected into the Tet-On dCas9 K562 cell line with the 3X CD81 guide array. Each bar represents 3 timepoints collected within the specified days, with bar heights showing the average perturbation across timepoints. Error bars represent the mean ± standard deviation of 3 replicates. Arrows point from WT effector to mutant form. **c** Timecourse of *CD81* repression following nucleofection of plasmids encoding DNMT3A-3L combinations and induction of the dCas9/MS2 complex by doxycycline for 5 days. *proposed mutant inferred from PRDM7. Lack of a C-terminal effector indicates that the effector was tested monovalently. Additional detailed data are shown in Supplementary Fig. 28. **d** Timecourse of CD81 MFI relative to the neutral DMD control following nucleofection of plasmids encoding UBE2E1 + DNMT3A combinations and induction of the dCas9/MS2 complex by doxycycline for 5 days. Additional detailed data are shown in Supplementary Fig. 29. **e** Timecourse of CD155 MFI relative to the neutral DMD control following nucleofection of plasmids encoding TET1 combinations and induction of the dCas9/MS2 complex by doxycycline for 5 days in the Tet-On dCas9 K562 cell line with 3X *CD155* guide array. Day 0 indicates the day of doxycycline washout. Each bar represents a single timepoint collected on the specified day. Error bars represent the mean ± standard deviation of 3 replicates. Lack of a C-terminal effector indicates that the effector was tested monovalently. Arrows point from WT to mutant form of the same effector. **f** Representative expression profiles illustrating three types of gene expression memory. CD81 and CD155 expression levels were measured 12 and 14 days after doxycycline washout, respectively, following 5 days of induction. Dashed black line indicates repression gate at the 1st percentile of the neutral condition. Solid black lines indicate geometric means. Source data are provided as a Source Data file.

Thus far, we identified individual effectors and combinations of effectors that transiently perturb gene expression, measuring effector strength and uncovering domain interactions. Next, we investigated durable effects on target gene expression produced by transient expression of the epigenetic editing complex (Fig. 6a). Following the initial 5-6 day effector recruitment phase, doxycycline was removed to halt any further production of dCas9 and the (std)MCP-fused effectors. Cells were cultured for twelve days following doxycycline removal, as transient KRAB repression dissipated by this timepoint (Supplementary Fig. 4b–d). While the majority of transcriptional effects had dissipated by Day 12 (Fig. 1g, i and Supplementary Data 3 and 4), marginal score analysis revealed 3 individual effectors with durable transcriptional perturbations, all of which participate in DNA methylation (Fig. 6b). Two of the identified effectors, DNMT3A-3L^17^ and DNMT3A, stably repressed target gene expression, while one effector, TET1, stably activated target gene expression. In contrast to DNMT3A-3L and DNMT3A, which are capable of de novo methylation of CpG sites^67–69^, TET1 oxidizes methylated cytosines to promote DNA demethylation^69,70^.

We followed up on each of these three domains - including different pairings suggested by screen data - with individual validation experiments. In contrast to the transient perturbations induced by KRAB and p65-HSF1, DNMT3A-3L showed moderate long-term repression both alone and in screen-identified combinations. DNMT3A-3L alone, UHRF1 + DNMT3A-3L, and PRDM4 + DNMT3A-3L repressed *CD81* in 23.4%, 19.7%, and 18.5% of cells, respectively, 12 days after doxycycline removal without significant effects on cell growth (Fig. 6c and Supplementary Figs. 27 and 28). This repression was guide-dependent and decayed slowly over 50 days, though similar experiments targeting *CD155* showed more complete and stable memory over 35 days (Supplementary Fig. 11d, e). We further tested a hypoactive mutant of UHRF1 and an inferred hypoactive mutant of PRDM4, mutating a conserved tyrosine that is catalytically active in closely related PRDM7^71,72^. Incorporating these mutations led to a 1.4-fold and 2.4-fold reduction in repression on average over the 50-day timecourse, which may be attributed to reduced catalytic activity or effector expression (Supplementary Figs. 12f, g and 28e).

For DNMT3A, we selected the combination with UBE2E1 for individual validation. Interestingly, we observed that this combination led to minimal complete silencing (2.1% cells fully silenced at 6 days after doxycycline removal) but resulted in a 49.1% reduction in mean CD81 expression across all cells (measured as MFI) at day 12 (Fig. 6d and Supplementary Fig 29a–c). This long-term downregulation was guide-dependent and decayed to 15.5% by day 50, with similar experiments targeting *CD155* showing more stable and complete silencing (Supplementary Fig. 11d,e). Notably, the downregulation effect on *CD81* was reduced by 2-fold when UBE2E1 was mutated at its catalytic cysteine and completely abolished when DNMT3A was catalytically inactivated (Supplementary Fig. 29c, d)^54,73^. Neither of these mutations significantly decreased effector expression (Supplementary Fig. 12d, f, g). These results indicate that DNMT3A is the primary mediator of this repressive epigenetic memory, but that UBE2E1 augments DNMT3A’s activity, likely through its catalytic function as an E2 UBL. The ability to downregulate gene dosage without complete silencing may be useful in therapeutic situations where maintained low-level gene expression is required.

In addition to long-term repression, we observed long-term epigenetic activation catalyzed by TET1 combinations. While TET1 has been previously used to reactivate the expression of target genes silenced by DNA methylation in synthetic or disease contexts^12,74^, marginal score analysis suggests that TET1 can also increase the expression of a basally expressed target gene not only transiently^69,75,76^ but also in a long-term manner. For individual validation, we selected the three TET1 partners with the highest activation from our screen - RNF20’s RING finger domain, SMYD1’s SET domain, and PRDM1’s PR/SET domain.

When compared to neutral DMD control, all three TET1 combinations and TET1 alone led to an increase in CD81 expression that lasted up to 50 days and reached up to 41.8% (Supplementary Figs. 30 and 31). Surprisingly, long-term activation of *CD81* was not caused by guide-directed targeting, as this effect was equally observed with non-targeting guides (Supplementary Fig. 11d). However, we did observe targeted long-term activation at *CD155* with *CD155*-targeting guides, which had previously shown stable complete silencing with DNMT3A combinations, suggesting gene-specific epigenetic susceptibility (Supplementary Fig. 11e). When compared to neutral DMD control targeting *CD155*, all three TET1 combinations and TET1 alone induced upregulation of *CD155* that reached up to 72.9% for RNF20 + TET1 and lasted up to 35 days (Fig. 6e). Long-term upregulation was not observed when targeting a catalytically dead form of TET1^70^ even though the mutation did not significantly impact expression (Supplementary Fig. 12f, g). While none of the TET1 combinations from screen significantly outperformed TET1 alone, we reasoned that combining TET1 with the strong activator p65-HSF1 might help to overcome the transient downregulation seen on day 0. The p65-HSF1 + TET1 combination retained both transient and long-term activation potential with transient activation reaching over 20-fold and long-term activation holding steady around 55% for up to 35 days (Fig. 6e, f).

## Discussion

In this work, we devise a platform technology called COMBINE for systematic, high-throughput functional screening of domain pairs. Importantly, COMBINE overcomes the length limitations and length-based biases of previous screening methodologies^28,29,36^ to enable the characterization of large protein domains and beyond. We implemented two sequencing readout strategies: direct paired-end sequencing for Library 1 and a barcode mapping approach for Library 2. Our comparison demonstrates that the barcoding approach is advantageous, showing lower dropout rates and length-independent performance for combinatorial domain candidates up to 2,094 amino acids in length. Notably, the barcoding approach circumvents PCR amplification of combinatorial candidates throughout the entire screening process, thereby eliminating PCR-biases and the risk of template switching between effectors.

Applying COMBINE to epigenetic regulation of gene expression, we test over 50,000 pairs of epigenetic reader, recruiter, writer, and eraser domains across two libraries. Our quantitative framework, incorporating marginal scores and synergy metrics, enabled comprehensive characterization at multiple levels: from broad class-level trends of epigenetic effector activity, to individual domain contributions to transcriptional modulation, to specific domain-domain interactions. This systematic analysis revealed both expected biological interactions, such as reader-writer synergies in PRC1 recruitment, and unexpected findings like the synergistic pairing of HDAC domains with UBE2E1 and VRK1. We also identified the potent KRAB + L3MBTL3 combination, which provided sgRNA position-dependent enhancement in target gene repression. By rescuing moderately potent sgRNAs, KRAB + L3MBTL3 broadened the effective targeting window and improved silencing under dose-limiting conditions. This robust activity enabled the improvement of an aptamer-based dual-directional CRISPR perturbation system^62,63^, in which one target gene is silenced while another is activated. The broader targeting window may be particularly valuable when design constraints restrict sgRNA positioning to suboptimal locations (e.g., allele-specific targeting). However, careful evaluation of potential off-target effects remains critical for any epigenome editing application. Long-term analysis after inducer washout identified DNA methylation as a primary mechanism for establishing epigenetic memory in our experimental system. Transcriptional profiles ranged from complete silencing to partial downregulation to activation in a gene-specific manner depending on the effector combinations, suggesting differential epigenetic susceptibility across genomic loci.

While our screen characterized diverse pairwise domain interactions, high-throughput screening of combinatorial domains presents several technical considerations. First, expression levels and stability can vary between fusion proteins, and some combinations could act through non-targeted mechanisms. These potential confounders could be addressed in future screens by directly fusing fluorescent proteins to domain combinations or by including non-targeting guide controls. Second, epigenetic effectors may behave differently across cellular, genomic, and protein recruitment contexts^27^. Therefore, we recommend performing screens tailored to specific contexts of interest, including the relevant cell type, target gene, targeting position relative to the gene, and effector recruitment strategy.

The COMBINE platform is generalizable and applicable beyond epigenetics to study many outstanding questions in combinatorial biology. The ability to systematically evaluate the functional interplay between entities–whether that includes DNA elements, RNA species, protein domains, or entire proteins–will unlock a sophisticated understanding of interactions in biology, where nothing acts in isolation.

## Methods

### Cell lines and cell culture

All experiments in this study were carried out in K562 cells (ATCC, CCL-243, female). Cells were cultured in a humidified incubator at 37 °C and 5% CO_2_, in RPMI 1640 (Gibco, 61870036) media supplemented with 10% fetal bovine serum (FBS) (Gibco, 26140095) and 1% penicillin-streptomycin (Gibco, 15140122). For Library 1 lentivirus generation, 293FT (Thermo Fisher Scientific) or Lenti-X 293 T (Takara) were cultured in a humidified incubator at 37 °C and 5% CO_2_, in DMEM (Gibco, 10566016) media supplemented with 10% fetal bovine serum (FBS) (Gibco, 26140095) and 1% penicillin-streptomycin (Gibco, 15140122). For Library 2 lentivirus generation, viral production cells (Gibco, A35347) were cultured according to the manufacturer’s instructions.

### Guide design and cloning

CRISPRa guides were generated using CHOPCHOP for the *CD81*, *CD55*, *CD58*, *CD151*, and *CD155* genes, and three were selected to tile each promoter region from −500-0 to the TSS (Supplementary Data 9, Guides)^77^. A tiered golden gate approach was developed to assemble a 3X guide array and to easily insert this array into a landing pad PiggyBac vector, which was cloned in part using the EMMA toolkit^78^, between divergent expression cassettes pTRE3G-dCas9-tagBFP and pEF1a-BlastR-P2A-rtTA3 (Supplementary Data 9, Plasmids, pMH224). Briefly, the three guides were cloned separately via BsmBI golden gate into three distinct guide expression plasmids containing different golden gate overhangs around the guide cassette (Supplementary Data 9, Plasmids, pMH213-pMH215). The guide scaffold contained MS2 aptamers in stemloop two and the tetraloop and was sourced from recent optimization^33^. These individual guide plasmids were then assembled via BsaI golden gate into a RFP dropout landing pad containing pSV40-mCherry for mammalian expression (Supplementary Data 9, Plasmids, pMH222). Guides were arranged in the 3X array such that the closest guide to the TSS was placed first, followed by the middle guide and then the furthest guide. The *CD81* 3X guide array plasmid was used directly for guide nucleofection in screening of Library 1 (Supplementary Data 9, Plasmids, pMH228). This 3X array was further assembled into pMH224 via a final BsmBI golden gate for PiggyBac insertion and constitutive guide expression, which was used for screening Library 2 (Supplementary Data 9, Plasmids, pMH260). Additional sgRNAs targeting before the TSS of *CD55*, *CD151*, and *CD274* were generated using CHOPCHOP (Fig. 5 and Supplementary Data 9, Guides)^77^. *CD55* and *CD151* guides were inserted into a lentiviral vector containing the recently optimized MS2 guide scaffold^33^ driven by human U6 promoter and SV40 promoter driven-mCherry marker. sgRNAs targeting upstream of the *NF1* TSS were designed using CRISPick^79^ and cloned into a transfectable vector with the optimized MS2 guide scaffold (U6 promoter) and mCherry marker (SV40 promoter). For dual-directional perturbation experiments, the *CD274* guide (Supplementary Data 9, Guides) was cloned into the human U6-driven PP7 guide scaffold within a lentiviral vector, where both MS2 sgRNA and PP7 sgRNA are expressed from distinct mouse and human U6 promoters, respectively.

### PB Tet-On dCas9 cell line generation

To generate Tet-On dCas9 K562 cell lines expressing pTRE3G-dCas9-tagBFP, pEF1a-BlastR-P2A-rtTA3 with or without 3X guide arrays, K562 cells were nucleofected with 600 ng of pMH224 with or without golden gate insertion of a 3X guide array (pMH260 for 3X *CD81*) and 200 ng of a separate PiggyBac transposase plasmid using SF Cell Line 96-well Nucleofector kit and protocol (Lonza, V4SC-2096) (Supplementary Data 9, Plasmids). Two days following nucleofection, a 10-day selection began with 10 μg/mL blasticidin (Gibco, A1113903). After selection, cells were expanded and assessed for BFP induction in response to 1 μg/mL doxycycline. This Tet-On dCas9 K562 (with *CD81* guide array) cell line was used for Library 2 HTS and Library 1 validation experiments, while this Tet-On dCas9 K562 (without *CD81* guide array) was used for validation of Library 2 effectors on additional genes.

An additional Tet-On dCas9 K562 (without *CD81* guide array) cell line was generated that expresses pTREtight-dCas9 and pEF1a-BlastR-P2A-rtTA3. K562 cells were co-transfected with Piggybac plasmid encoding both pTREtight-dCas9 and pEF1a-BlastR-P2A-rtTA3 (without transposases) and PiggyBac transposase plasmid using Lipofectamine 2000 (Invitrogen). Two days following transfection, 20 μg/mL blasticidin (Gibco, A1113903) selection was performed for at least 10 days. This Tet-On dCas9 K562 (without *CD81* guide array) cell line was used for Library 1 HTS.

### Library 1 design and cloning

A literature review was conducted to cover critical components of the key repressive epigenetic multiprotein complexes and structural factors for chromatin organization. The majority of library members were selected based on previous arrayed studies employing truncation-based co-immunoprecipitation experiments or luciferase assays, except for the few members from high-throughput studies^24^. Peptides shorter than 80 amino acids were expanded equally on either side to reach 80 amino acids in total. Start codons were removed as effectors were to be fused to the C-terminus of aptamer binding protein. Amino acid sequences were back-translated with mammalian species codon optimization and forbidding 3′ end 6-mer of both amplification primers motifs (GGTGTG and ATGGCC) by Geneious Prime. Second codon optimization was performed for human codon usage, removing several type IIS restriction enzyme sites (BbsI, BsaI, BsmBI, BspQI, BtgZI, and SapI) and constraining GC content to between 35% and 65% in every 50 nucleotides window by DNA chisel^80^. Forward amplification primer binding handle (TCCaGACCGTTCaGGTGTG adapted from previous study^24^), BsaI binding site (GGTCTCT), and overhang (GTCA) facilitating ‘Glutamic-acid (E) – Serine (S)’ peptide overhang for C-terminus of XTEN16 linker were appended to 5′ of the gene fragment (Supplementary Fig. 2a). Overhang (AGTG) for ‘Serine (S) – Glycine (G)’ peptide overhang at N-terminus of XTEN16 linker, BsaI binding site (CGAGACC), and reverse amplification primer binding handle (gGCCaTGCGGaATGGGTTA) were appended to 3′ of the gene fragment. Optimized golden gate assembly overhangs were selected based on a previous study^81^. A few BsmBI sites, BsaI sites, and 3′ end 6-mer of both amplification primers motifs (GGTGTG and ATGGCC) that were unintentionally generated by former procedures were manually modified by silent mutations. Finally, library members that did not pass the Gblock or Eblock complexity test were human codon-optimized by IDT webtool, and again BsmBI, BsaI sites, and 3′ end 6-mer of both amplification primers motifs were manually modified by silent mutations.

Double-stranded gene fragments were synthesized (IDT and Twist Biosciences) and then pooled manually without PCR amplifications to make a 20 nM solution with the molarity of each fragment increasing proportional to its length with a slope of 1. For example, we added 1.5-fold of the 450 bp gene fragment compared to the 300 bp gene fragment. The pooled library was subcloned into KanR1_receiver (pCM001) and KanR2_reveiver (pCM002) plasmids by two separate golden gate reactions. For a total volume of 40 μL golden gate reaction, we used 185 ng of KanR1 (pCM001) or KanR2 (pCM002) receiver plasmid, 10 μL of 20 nM pooled library for ~2:1 molar ratio between backbone:insert, 2 μL of BsaI-HFv2 (NEB, 20000 U/mL), 2 μL of T4 DNA ligase (NEB, 400000 U/mL), and 4 μL of 10X T4 DNA ligase buffer (NEB). The thermocycling protocol was 30 cycles of 5 min digestion at 37 °C and 5 min ligation at 16 °C, followed by a final 5 min digestion at 37 °C, 5 min heat inactivation at 60 °C and an extra 20 min heat inactivation at 70 °C. The reactions were then purified with DNA Clean & Concentrator-5 (Zymo) and were eluted by 8 μL of nuclease-free water (Ambion). 1 μL of purified DNA was transformed into 25 μL of Endura electrocompetent cells (Lucigen) following the manufacturer’s instructions with 2 mL of prewarmed recovery media. 2 mL of cells were plated onto a 245 mm × 245 mm plate, and serially diluted cells were plated onto 100 mm diameter plates for the estimation of colony numbers. After overnight incubation at 30 °C, colonies were collected, and plasmid libraries were extracted with Maxiprep (Machery-Nagel). Colony coverage was at least 34000X for 155 library members.

For the final bivalent library construction, the LV_AmpR_backbone (pCM003) was predigested with FastDigest Esp3I (Thermo Fisher Scientific) and gel extracted. For a total volume of 100 μL golden gate reaction, we used 800 ng of predigested and gel-extracted LV_AmpR_backbone (pCM003), 1000 ng of N-terminus effector (KanR1_recevier) library, 1000 ng of C-terminus effector (KanR2_receiver) library, 10 μL of FastDigest Esp3I (Thermo Fisher Scientific), 5 μL of T4 DNA ligase (NEB, 400000 U/mL), and 10 μL of 10X T4 DNA ligase buffer (NEB). The thermocycling protocol was initial 5 min digestion at 37 °C, 30 cycles of 5 min digestion at 37 °C and 10 min ligation at 16 °C, final 20 min ligation at 16 °C, final 5 min digestion at 37 °C, and 20 min heat-inactivation at 75 °C. The golden gate reaction was then isopropanol precipitated into 10 μL of nuclease-free water (Ambion)^82^. Again, 1 μL of purified DNA was transformed into 25 μL of Endura electrocompetent cells (Lucigen) following the manufacturer’s instructions with 2 mL of prewarmed recovery media. 2 mL of cells were plated onto a 245 mm × 245 mm plate and serially diluted cells were plated onto 100 mm diameter plates for the estimation of colony numbers. After overnight (15 hours) incubation at 30 °C, colonies were collected, and plasmid pools were extracted with Maxiprep (Machery-Nagel). Colony coverage for 1 bioassay plate was at least 1100X for 155^2^ library members. Two separate maxi-prepped DNA solutions from two bioassay plates were mixed to achieve at least 2200X colony coverage of library members.

### High-throughput assay to measure transcriptional effects of Library 1

Low passage 293FT cells (Invitrogen) or Lenti-X (Takara) 293 T cells were grown in DMEM (Invitrogen, 10566-016) supplemented with 10% FBS (Gibco) and 1% Penicillin-Streptomycin (Gibco). For one T225 flask, 39.9 μL of 1 mg/mL PEI MAX (Polysciences) and 1 mL of DMEM were mixed by inversion and incubated at room temperature for 10 min. During incubation, 5.6 μg of pMD2.G, 11.3 μg of psPAX2, and 22.7 μg of target plasmid or plasmid library were added to new 1 mL of DMEM and were thoroughly mixed by vortexing. After incubation, plasmids + DMEM mixture was added to PEI MAX + DMEM mixture followed by 30 sec vortexing. The final ~2 mL mixture of plasmids + PEI MAX + DMEM was incubated at room temperature for 30 min. During incubation, 293FT or Lenti-X 293 T cells were split by TrypLE (Gibco) and 33.4 million cells were added to a standing T225 flask in 5 mL of fresh growth media. After the 30 min incubation, plasmids + PEI MAX + DMEM mixture was added to the flask, and the flask was gently mixed by swirling. The flask was incubated for 5 min in the upright position. Following the 5 min incubation, fresh growth media was added up to 45 mL, and cells were incubated at 37 °C, 5% CO_2_ overnight. The next day (Day 1), the media was changed to 45 mL of fresh growth media and incubated for an additional 2 days, after which the supernatant was collected and concentrated by Lenti-X concentrator (Takara) following the manufacturer’s protocol.

Upon infection of 40 million PB Tet-On dCas9 cells (without *CD81* guide array) with 200 μL of lentivirus per replicate on Day −13, cells were cultured in growth media containing 1-2 μg/mL doxycycline (Supplementary Fig. 5). In parallel, 2 control cell lines (negative control: PB Tet-On dCas9 cells without any effector & positive control: PB Tet-On dCas9 cells with LV-pTRE3G-PuroR-T2A-stdMCP-eGFP-KRAB (ZNF10) preinstalled by lentiviral infection) started doxycycline incubation as well. Doxycycline was replaced or added daily, maintaining the concentration of 0.5-2 μg/mL to compensate for the short half of doxycycline in the media. 1 day post-infection (Day −12), puromycin selection started with a 1 μg/mL concentration. To simultaneously measure the lentiviral titer, subsets of cells were passaged to 96 well plates for the viability assay. 3 days after the start of puromycin selection (Day −9), lentiviral titer was measured by the CellTiter-Glo 2.0 Cell Viability Assay (Promega G9242) as previously described^82^. Measured viability was 0.25-0.29 suggesting successful low MOI infection. Puromycin selection was continued for an additional 3 days until Day −6. We observed an increase in the percentage of live cells by trypan blue staining (Thermo Fisher Scientific) during the additional 3 days of selection, suggesting the outgrowth of infected cells over debris from dead cells.

After a total of 6 days of puromycin selection and outgrowth (Day −6), 39.2 million surviving cells were mixed with 0.8 million positive control cells (LV-pTRE3G-PuroR-T2A-stdMCP-eGFP-KRAB (ZNF10)) per replicate. On Day −6, mixed cells were nucleofected with the *CD81* targeting 3X guide array plasmid (pMH228). For each cuvette, 4 million cells were nucleofected with 4.5 μg of pMH228 following the manufacturer’s K562-specific protocol (4D X-unit, FF-120 program, Lonza). For each replicate, 40 million cells were nucleofected by 10 cuvettes. 4 million positive control cells were also nucleofected by 1 cuvette. 3 days post-nucleofection (Day −3), mCherry-positive cells were sorted. To reduce the variability generated by sgRNA expression level, a population expressing moderate levels of mCherry was sorted (Supplementary Fig. 5b). In total, ~9.7 million cells (~400X coverage) were sorted for each replicate. 3 days post-sorting (Day 0), at least 11.5 million cells per replicate were frozen for future FACS sorting. The remaining half of at least 11.5 million cells were centrifuged and passaged to media without doxycycline to stop the expression of dCas9 and the stdMCP-combinatorial effectors. Cells were continuously expanded until Day 6 in culture media without doxycycline. On Day 6, 50 million cells were frozen for each replicate. Cells were again continuously expanded until Day 12. On Day 12, 120 million cells were frozen for each replicate.

On the day of FACS, frozen cells from various timepoints (Day 0, 6, and 12) were thawed briefly and washed twice with eBioscience Flow Cytometry Staining Buffer (Invitrogen). APC Mouse Anti-Human CD81 antibody (Cat# 561958, BD Bioscience) was diluted 1:10 in 100 μL stain buffer per 0.1 million cells. Staining was performed on a 3D rotary mixer in a cold room (4 °C) for 1 hour. Cells were washed twice with stain buffer and sorted using a FACSAria Fusion Special Order Research Product (BD Bioscience). mCherry-negative singlet populations were separated into two populations based on CD81 level (Supplementary Fig. 5c). Sorted cells were frozen at −80 °C until genomic DNA extraction.

Genomic DNA (gDNA) was extracted using columns from the Quick-DNA Miniprep Plus kit (Zymo Research) and reagents from the Quick-DNA Midiprep Plus kit (Zymo Research) with the following modifications in the protocol. Cells were split into multiple columns if the number of cells was larger than 3 million. Per 3 million cells, excess genomic lysis buffer (Beta-Mercaptoethanol added) with a volume of 1.6 mL from the Quick-DNA Midiprep Plus kit (Zymo Research) was used instead of BioFluid & Cell Buffer from the Quick-DNA Miniprep Plus kit (Zymo Research). After the addition of 1.6 mL genomic lysis buffer to the cells, the mixture was vortexed thoroughly. Cells were lysed in genomic lysis buffer for 1 hour at room temperature with 3D rotation. Excess genomic lysis buffer ensured complete lysis of the cells, yielding a high amount of extracted gDNA. Except for the described cell lysis step, we followed the manufacturer’s protocol for the Quick-DNA Miniprep Plus kit (Zymo Research).

Enriched combinatorial library members were PCR amplified from the gDNA using the following conditions. For one 50 μL of the PCR reaction, 25 μL of 2X NEBNext HiFi (NEB), 1 μg of gDNA, and a final concentration of 0.5 μM for each forward and reverse NGS primers (Supplementary Data 9) were prepared and mixed on ice. One-step NGS primers were designed to have 0 ~ 7 random spacer nucleotides to increase the base diversity during the initial sequencing cycles^83^. The thermocycling protocol was 1 cycle of 98 °C for 3 min, 27 cycles of 98 °C for 10 sec, 64 °C for 30 sec, 72 °C for 75 sec, and final 1 cycle of 72 °C for 2 min. After thermocycling, PCR reactions were pooled and run through multiple lanes in 1.2% TBE gel. Rectangular regions of the gel spanning from 800 bp ~ 2 kb were excised, and PCR products were extracted using Monarch DNA Gel Extraction Kit (NEB). The gel-extracted library was quantified by Nanodrop (Thermo Fisher Scientific), Qubit HS kit (Thermo Fisher Scientific), Bioanalyzer (Agilent Technologies), and KAPA Library Quantification Kit (Roche). We noticed approximate agreement in quantification between Qubit and Bioanalyzer while KAPA library quantification exhibited the most discrepancy, potentially due to differences in the size between our library and the standards provided by the kit. Based on the results of Qubit and Bioanalyzer, the library was pooled with ~10% PhiX control (Illumina) and sequenced on a NextSeq2000 (Illumina) with 2 × 300 bp P2 kits (Illumina). In total, two 2 × 300 bp P2 kits were used. Libraries from Day 0 and 6 were pooled together and sequenced with one kit, and the Day 12 library was sequenced separately using the second kit.

### NGS analysis of bivalent domains from Library 1

R1 and R2 reads were mapped to individual effectors by minimap2^84^ with ‘map-iclr’ option (Supplementary Data 11; Minimap_batch_Lib1.py^85^). Count tables for bivalent combinations were generated by pairing R1 and R2 mapped effectors. If the sum of NGS reads from High and Low bins was less than 40 in at least one replicate, the bivalent combination was considered a dropout. Some combinations are present only in the LOW bin or the HIGH bin due to tight sorting strategy for Library 1 (Supplementary Fig. 5), which could result in division by zero when calculating the HIGH/LOW ratio. To prevent this issue, we added a count of +5 to non-dropout counts. Read counts were normalized by the sum of each bin, and the enrichment scores were calculated as the ratio of the normalized counts in the High bin versus the Low bin.

### Secondary validation of individual and bivalent effectors from Library 1

To harness previously ordered effector gene fragments, we again generated a series of landing pad plasmids for nucleofection-based delivery of effectors. For bivalent domain candidates, similar to the library cloning procedure, 3 sequential golden gate reactions were performed. The landing pad plasmid for the 1st golden gate reaction was the same KanR1_receiver (pCM001) as the library cloning, whereas KanR2_individual_validation_receiver (pCM023) was used as the landing pad plasmid for the 2nd golden gate reaction. For the final golden gate reaction, the landing pad was pCAG_AmpR_backbone (pCM024), which enables the constitutive expression of the effector by the CAG promoter. For monovalent domain plasmid cloning, 2 sequential golden gate reactions were performed. For the 1st golden gate reaction, the landing pad plasmid was KanR_monovalent_receiver (pCM025). For the 2nd golden gate reaction, the landing pad plasmid was the same as the bivalent domain candidate cloning, pCAG_AmpR_backbone (pCM024).

For cloning linear gene fragments into KanR1_receiver (pCM001), KanR2_individual_validation_receiver (pCM023), and KanR_monovalent_receiver (pCM025), 0.25 μL of 60 ng/μL landing pad plasmids, 2.5 μL of 20 nM gene fragment, 0.5 μL of 10X T4 ligase buffer (NEB), 0.25 μL BsaI-HFv2 (NEB, 20000 U/mL), 0.25 μL T4 Ligase (NEB, 400000 U/mL) and 1.25 μL nuclease-free water (Ambion) was mixed well resulting in a total of 5 μL reaction. The thermocycling condition was 30 cycles of 37 °C for 1 min and 16 °C for 1 min followed by 1 cycle of 37 °C for 5 min and 75 °C for 5 min. For the final golden gate reaction, 0.5 μL of 100 ng/μL pCAG_AmpR_backbone (pCM024), 1 μL of 50 ng/μL N-terminus effector plasmid, 1 μL of 50 ng/μL C-terminus effector plasmid, 1 μL of FastDigest Esp3I (Thermo Fisher Scientific), 0.5 μL of T4 DNA ligase (NEB, 400000 U/mL), 1 μL of 10X T4 buffer (NEB), and 5 μL of nuclease-free water (Ambion) were mixed well resulting in a total of 10 μL reaction. KanR_monovalent_receiver (pCM025) was used instead of KanR1_receiver (pCM001) or KanR2_individual_validation_receiver (pCM023) for cloning the monovalent effector plasmid. The thermocycling protocol was the same: 30 cycles of 37 °C for 1 min and 16 °C for 1 min followed by 1 cycle of 37 °C for 5 min and 75 °C for 5 min.

Nucleofection of effector plasmids was performed with SF Cell Line 4D-Nucleofector X Kit S following the manufacturer’s protocol (4D X-unit, FF-120 program, Lonza). PB Tet-On dCas9 K562 cell line (with *CD81* guide array) was preinduced with 1–2 μg/mL doxycycline starting 0–3 days before the day of nucleofection. For complete details refer to Supplementary Data 10. On the day of measurement, ~300 μL of cells were washed twice with eBioscience Flow Cytometry Staining Buffer, and 99 μL staining buffer with 1 μL of APC Mouse Anti-Human CD81 antibody (Cat# 561958, BD Bioscience) was used per well in 96 well plates. Following 1 hour of incubation at 4 °C, cells were washed twice, and the CD81 level was measured by Attune NxT Flow Cytometer (Thermo Fisher Scientific). Empty vector pUC19 nucleofected cells were used as control cells for gating on BFP, GFP, mCherry, and CD81 (APC) levels.

### Library 2 design, cloning, and barcode mapping

A literature review was conducted to identify minimally active catalytic epigenetic effectors across 11 well-established classes: DNA demethylation machinery (DNDM), DNA methyltransferase (DNMT), E2 and E3 ubiquitin ligases (UBL), histone acetyltransferase (HAT), histone arginine methyltransferase (HRMT), histone deacetylase (HDAC), histone demethylase (HDM), histone deubiquitinase (DUB), histone kinase (HK), histone lysine methyltransferase (HKMT), and histone phosphatase (HP). Human proteins associated with each class were identified using Gene Ontology terms, except for HKMTs, which were identified as human proteins containing SET domains in addition to GO terms. Within each list, each protein was manually reviewed to assess for possible catalytic activity and to search for catalytically active truncations. In total, 195 catalytic domains were identified across all classes, with ~75% sourced directly from literature, while the remaining domain bounds were sourced from alignment with closely related proteins or simply estimated from Pfam annotations. Additionally, three non-catalytically active controls were included: RYBP for silencing^37^, a 400 aa fragment of DMD determined to be neutral from HT-recruit^24^, and HSF1 for activation^10^. All domains and references are included in Supplementary Data 2.

Amino acid sequences for each effector were codon optimized for human expression using DNA Chisel with constraints to avoid restriction enzyme sites (BsaI, BsmBI, BbsI, BtgZI), avoid patterns (5 × 3-mer, 2 × 15-mer, 2 × 12-mer, 8 x homopolymer), and enforce GC content between 35–65% in each 50 bp window^80^. Each effector was ordered from Twist Biosciences as a clonal gene in pTwist Kan High Copy with flanking sequences containing nested BsmBI and BsaI golden gate sites to facilitate cloning bivalent combinations (Supplementary Fig. 3a and Supplementary Data 9, Misc., 5′ and 3′ Effector Flank). All golden gate overhangs were sourced from a 15 member high fidelity set, and all landing pads contained CcdB dropout genes to improve cloning efficiency^81^. Individual effector plasmids were transformed into MACH1 cells (Thermo Fisher Scientific) and miniprepped using QIAprep Spin Miniprep Kit (Qiagen, 27106). Because lentiviral transduction efficiency decreases roughly 3.5X per additional kilobase of DNA^30^, effector plasmids were pooled exponentially by effector size by manually adding 0.2*3.5^(x/1000) pmol of plasmid DNA, where x is the length of the effector in basepairs (Supplementary Fig. 3b, c). The BsmBI sites in this pool (EF2 pool) were later used to clone into the N-terminal Effector 2 position, and the BsaI sites were used to subclone the pooled effectors into a new high copy Chloramphenicol resistant backbone via golden gate reaction (50 ng pMH249 (Supplementary Data 9, Plasmids), 2-fold molar excess EF2 pool, 1 μL BsaI-HFv2 (NEB, R3733L), 1 μL T4 ligase (NEB, R0202L), 2.5 μL T4 ligase buffer (NEB, B0202S), and H_2_O to 25 μL incubated at 37 °C for 5 min, followed by 30 cycles of 37 °C for 5 min and 16 °C for 10 min, followed by final ligation at 16 °C for 20 min, final digestion at 37 °C for 30 min, and heat inactivation at 80 °C for 20 min) (Supplementary Fig. 3d). The reaction was then cleaned using DNA Clean & Concentrator-5 (Zymo, D4013) and eluted in 6 μL H_2_O, before electroporating 2 μL into 25 μL of Endura electrocompetent cells following the manufacturer’s instructions (Biosearch Technologies, 60242-1). Recovered cells were plated onto a 245 mm × 245 mm plate and incubated overnight at 37 °C. The next day, colonies were scraped from the plate and the plasmid pool was maxiprepped (Machery-Nagel, 740424.50). The resulting EF1 pool contained BsmBI golden gate sites at the same 4 bases as the BsaI insertion of the effector to allow for subsequent cloning into the C-terminal Effector 1 position (Supplementary Fig. 3e).

Before the final bivalent assembly of the EF1 and EF2 pool into our lentiviral landing pad, the XTEN16 linker and variable barcodes were prepared. The XTEN16 linker was ordered as two ssDNA oligos with the correct overhangs for assembly between the EF1 and EF2 position and annealed by equimolar mixing at room temperature (Supplementary Fig. 3f and Supplementary Data 9, Misc.). Variable barcodes were ordered from Integrated DNA Technologies (IDT) as a 20xN ssDNA oligo flanked by NGS primer binding sites and BsmBI golden gate sites for assembly after EF2 (Supplementary Fig. 3g and Supplementary Data 9, Misc.). The oligo was ordered preannealed with the reverse complement of the 3′ flanking sequence to facilitate filling in of the bottom strand of the barcode and 5′ flanking sequence by polymerase extension (2.5 μL of preannealed oligos at 10 uM, 12.5 μL KAPA HiFi HotStart ReadyMix (Roche KK2601), and H_2_O to 25 μL followed by incubation at 98 °C for 3 min and 72 °C for 30 min). The extended barcode was cleaned with DNA Clean & Concentrator-5 (Zymo, D4013) and eluted in 20 μL H_2_O, before predigestion (1 μg extended barcode, 2 μL Esp3I (Thermo Fisher Scientific, FD0454), 2 μL 10X FastDigest buffer (Thermo Fisher Scientific, B64), and H_2_O to 20 μL incubated at 37 °C for 25 min). The digested barcode was cleaned with DNA Clean & Concentrator-5 (Zymo, D4013) and diluted to 2 ng/μL. Final bivalent assembly was performed via 5-piece golden gate reaction into our lentiviral landing pad, which was cloned in part using the EMMA toolkit^78^, containing pTRE3G-MCP-XTEN80 and pSV40-PuroR cassettes (250 ng pMH276 (Supplementary Data 9, Plasmids), 1X molar EF1 pool, 1X molar annealed XTEN16 linker, 1X molar EF2 pool, 1X molar digested barcode, 1 μL Esp3I (Thermo Scientific, FD0454), 1 μL T4 ligase (NEB, R0202L), 2.5 μL T4 ligase buffer (NEB, B0202S), and H_2_O to 25 μL incubated at 37 °C for 5 min, followed by 30 cycles of 37 °C for 5 min and 16 °C for 10 min, followed by final ligation at 16 °C for 20 min, final digestion at 37 °C for 30 min, and heat inactivation at 80 °C for 20 min) (Supplementary Fig. 3h). The reaction was then cleaned using DNA Clean & Concentrator-5 (Zymo, D4013) and eluted in 6 μL H_2_O, before electroporating 2 × 2 μL into 25 μL of Endura electrocompetent cells each following the manufacturer’s instructions (Biosearch Technologies, 60242-1). Recovered cells were serially diluted and plated onto 100 mm diameter plates for the estimation of colony numbers, while the remaining cells were plated onto several 245 mm × 245 mm plates. After overnight incubation at 30 °C, colonies on dilution plates were counted, and enough large plates were scraped and maxiprepped (Machery-Nagel, 740424.50) such that the average coverage was between 100-150X colonies per member. Strict coverage constraints were necessary both to adequately represent shorter effector pairs and to ensure that there were few enough barcodes to map via long-read sequencing.

Long-read nanopore sequencing was used to map barcodes to bivalent effector pairs. The plasmid pool was prepared for sequencing using two Cas9 digestion steps followed by nanopore adapter ligation in an attempt to bias reads towards the barcoded bivalent effector region (Supplementary Fig. 3i). First, two Alt-R *S.p*. Cas9 (IDT, 1081060) RNP pools were made, one containing the outer guides M1 and P1 and the other containing the inner guides M2 and P2, by prepooling 1 μL of each 100 μM crRNA and following the Alt-R CRISPR-Cas9 System protocol (Supplementary Data 9, Guides). The plasmid pool was then digested with the outer guides M1 and P1 (5 μg plasmid pool, 10 μL M1 + P1 Cas9 RNP, 4 μL 10X rCutSmart buffer (NEB, B6004S), and H_2_O to 40 μL incubated at 37 °C for 1 hour). The reaction was stopped by the addition of Proteinase K ( + 5 μL 20 mg/mL Proteinase K (Zymo, D3001-2-20), incubated at 56 °C for 10 min). A 1X AMPure XP bead (Beckman Coulter, A63881) cleanup was performed before dephosphorylation of ends followed by second digestion with the inner guides M2 and P2 (24 μL eluate, 3 μL Quick CIP (NEB, M0525S), and 3 μL 10X rCutSmart buffer (NEB, B6004S) incubated at 37 °C for 10 min and heat inactivated at 80 °C for 2 min, followed by addition of 10 μL M2 + P2 Cas9 RNP and incubation at 37 °C for 1 hour). The reaction was stopped by the addition of Proteinase K ( + 5 μL 20 mg/mL Proteinase K (Zymo, D3001-2-20), incubated at 56 °C for 10 min). A 0.5X AMPure XP bead (Beckman Coulter, A63881) cleanup was performed before dA tailing using Taq polymerase (34 μL eluate, 1 μL 10 mM dNTP mix (NEB, N0447S), 1 μL Taq polymerase (NEB, M0267S), and 4 μL 10X rCutSmart buffer (NEB, B6004S) incubated at 72 °C for 5 min). Another 0.5X AMPure XP bead (Beckman Coulter, A63881) cleanup was performed before nanopore adapter ligation (60 μL eluate, 25 μL Ligation Buffer (LNB, Oxford Nanopore, SQK-LSK114), 10 μL NEBNext Quick T4 DNA Ligase (NEB, E6056S), and 5 μL Ligation Adapter (LA, Oxford Nanopore, SQK-LSK114) incubated at room temperature for 20 min). A final AMPure XP bead (Beckman Coulter, A63881) cleanup was performed following the SQK-LSK114 protocol using the Short Fragment Buffer (SFB, Oxford Nanopore, SQK-LSK114). The resulting library was quantified using Qubit 1X dsDNA High Sensitivity Assay (Invitrogen, Q33231) and diluted to 20 fmol in 12 μL elution buffer (EB, Oxford Nanopore, SQK-LSK114) for sequencing on a MinION flow cell (Oxford Nanopore, FLO-MIN114) followed by a PromethION flow cell (Oxford Nanopore, FLO-PRO114M).

Nanopore reads were basecalled and duplexed using Dorado’s super-accuracy model v4.1.0 before conversion to.fastq file format by executing supduplex2fastq.sh on an appropriately configured GPU virtual machine. Barcode to effector pairings were then mapped by executing bc_extraction.py. Briefly, Effector 1, Effector 2, and barcode sequences were extracted from each read using cutadapt and appropriate flanking sequences before effectors were mapped to individual effectors using minimap2^84,86,87^. Effector mappings with <75% identity were discarded. The mapped barcodes from all sequencing runs were then combined into a single table of unique pairings and filtered to remove any duplicate barcodes (mappedBCs_filt.csv). In total, 7.8 million barcodes were mapped and represented every possible bivalent combination with the number of barcodes mapped scaling exponentially by bivalent effector length (Supplementary Fig. 3j, k).

### High-throughput assay to measure transcriptional effects of Library 2

A pooled lentiviral library was generated from Library 2 using the LV-MAX kit (Gibco, A35348) and protocol in a 1 L flask. Lentivirus was concentrated 10X using Lenti-X concentrator following the manufacturer’s instructions (Takara, 631232). Functional titer was measured by transducing 40,000 K562 cells per well of a 96 well plate with serial dilutions of lentivirus and 8 μg/mL polybrene (Millipore, TR-1003-G) via 90 minute spinfection at 1000 g and 32 °C. After overnight incubation, media containing lentivirus was replaced with fresh media and cells were allowed to recover for another 24 hours before being split equally into media with and without puromycin (2 μg/mL final, Gibco, A1113803). After five days of selection, cell survival in each well was quantified on a Tecan Spark plate reader using CellTiter-Glo 2.0 Cell Viability Assay (Promega, G9242). Percent survival was calculated as the ratio of luminescence in the presence versus absence of puromycin for each lentiviral dilution, and functional lentiviral titer was calculated and averaged for all dilutions with 5-30% survival. Two independent full scale transductions were then performed by transducing the PB Tet-On dCas9 3X *CD81* K562 cell line at 100X library coverage and an MOI < 0.1 with 8 μg/mL polybrene (Millipore, TR-1003-G) via 90 minute spinfection at 1000 g and 32 °C. After overnight incubation, media containing lentivirus was replaced with fresh media and cells were allowed to recover for another 24 hours before starting selection with 2 μg/mL puromycin for 5 days (Gibco, A1113803).

To assess bivalent chromatin effector activity in high-throughput, engineered cells were induced with 1 μg/mL doxycycline in 200 mL media in a 1 L Erlenmeyer flask, maintaining >1000X library coverage and replacing doxycycline media each day. After 5 days of induction (Day 0 timepoint), roughly 150 million cells (~3750X coverage) were frozen in 10 mL media supplemented with 10% DMSO at −80 °C in a 15 mL falcon tube. Roughly 50 million remaining cells (~1250X coverage) were passaged to assess memory by washing twice with DPBS (Gibco 14190144) and resuspending in 400 mL fresh media without doxycycline in a 2 L Erlenmeyer flask. Every 3 days for 24 total days, two frozen stocks of ~3000X cells were made as described above, and cells were diluted to a minimum density of 1e5/mL in 400 mL media in a 2 L Erlenmeyer flask. Two frozen timepoints, Day 0 and Day 12, were chosen to assess transient effector activity and epigenetic memory on *CD81* (Supplementary Fig. 6a). For a given timepoint and replicate, the frozen aliquot was thawed in a 37 °C water bath for 4 min before being diluted in 35 mL stain buffer (BD, 554656) and pelleted at 300 g for 10 min. The cell pellet was resuspended in 14 mL stain buffer containing 750 μL PE-conjugated CD81 monoclonal antibody (Invitrogen, MA1-10292), and the tube was rotated for 30 min at 4 °C. After staining, cells were spun down at 300 g for 8 min and washed twice with 15 mL stain buffer before resuspending in 15 mL and straining through a 40 μM filter. Using a BD FACSAria Fusion Special Order Research Product, between 7–13 million cells were sorted from the top and bottom 25% of the PE fluorescence distribution (Supplementary Fig. 6b-d).

To measure bivalent effector representation in each sorted population, genomic DNA was harvested using NucleoSpin Blood L kit and protocol (Machery-Nagel, 740954.20), adding 200 μL Monarch RNAse A (NEB, T3018L) before lysis and using high-yield elution recommendations. Then NGS libraries of bivalent effector barcodes were prepared using two sequential PCR steps. In PCR1, barcodes were amplified from gDNA using primer pairs containing staggers to diversity NGS reads (Supplementary Data 9, Primers, MH160-MH175) (up to 100 μg gDNA, 24 μL PCR1 primer set at 50 μM each, 600 μL NEBNext Ultra II Q5 MM (NEB, M0544L), and H_2_O to 1200 μL, divided into 12 × 100 μL reactions in a PCR plate and incubated at 98 °C for 30 sec, 20 cycles of 98 °C for 10 sec and 65 °C for 75 sec, final extension at 65 °C for 5 min). Split reactions were pooled and mixed thoroughly before 25 μL were cleaned using a two sided AMPure XP bead (Beckman Coulter, A63881) cleanup from 0.7X to 1.8X. In PCR2, sample indices were added along with flow cell binding sequences p5 and p7 (50-100 ng purified PCR1, 5 μL PCR2 primer set at 10 μM each, 25 μL NEBNext Ultra II Q5 MM (NEB, M0544L), and H_2_O to 50 μL, incubated at 98 °C for 30 sec, 7 cycles of 98 °C for 10 sec and 65 °C for 75 sec, final extension at 65 °C for 5 min). The reactions were cleaned using a two sided AMPure XP bead (Beckman Coulter, A63881) cleanup from 0.7X to 1.5X. Each sample was quantified using Qubit 1X dsDNA High Sensitivity Assay (Invitrogen, Q33231) and 60 ng of each were pooled into a single NGS library that was sequenced on a NextSeq 2000 using a P3 2 × 51 cycle paired-end run (Illumina, 20040559).

### Targeted nanopore sequencing to assess barcode swapping in Library 2

To address the issue of barcode swapping, which is commonly associated with the use of barcodes in lentiviral libraries^88^, Library 2 contained only a short 39 bp homologous sequence between the end of the effector fusion and the barcode. Still, to quantitatively measure lentiviral recombination between bivalent effector pairings and barcodes, barcode swapping was assessed post-integration via long-read nanopore sequencing using Cas9-guided adapter ligation (Supplementary Fig. 7a)^89^. Genomic DNA from one full-scale lentiviral infection replicate was extracted using Quick-DNA Midiprep Plus kit and protocol (Zymo, D4075). An Alt-R *S.p*. Cas9 (IDT, 1081060) RNP pool was made by prepooling 1 μL of crRNAs M1, M2, P1, and P2 at 100 μM and following the Alt-R CRISPR-Cas9 System protocol (Supplementary Data 9, Guides). Genomic DNA was dephosphorylated before Cas9 cleavage and dA-tailing (5 μg gDNA, 3 μL Quick CIP (NEB, M0525S), 3 μL 10X rCutSmart buffer (NEB, B6004S), H_2_O to 30 μL incubated at 37 °C for 10 min and heat inactivated at 80 °C for 2 min, followed by addition of 10 μL M1 + M2 + P1 + P2 Cas9 RNP, 1 μL dATP at 10 mM (NEB, N0440S), and 1 μL Taq polymerase (NEB, M0267S) and incubation at 37 °C for 20 min and 72 °C for 5 min). The reaction was cleaned using AMPure XP beads (Beckman Coulter, A63881) at 0.5X and DNA eluted in 60 μL H_2_O. Nanopore adapters were ligated following the SQK-LSK114 protocol, and the resulting library was sequenced on a MinION flow cell (Oxford Nanopore, FLO-MIN114). Nanopore reads were basecalled and converted to.fastq as previously described. Sequences containing complete reads through both effector positions were extracted using cutadapt and appropriate flanking sequences^87^. Barcode to effector pairings were mapped using bc_extraction.py^85^ (Supplementary Data 11), modified to retain all found barcodes, which were then referenced against mappedBCs_filt.csv. Reads from any mismatches between barcode to effector pairings were manually examined to determine the mismatch position (Supplementary Fig. 7b).

### NGS analysis of barcodes and bivalent domains from Library 2

NGS reads were converted into barcode counts using the script count_barcodes.py^85^ (Supplementary Data 11). In summary, reads were merged with fastp before barcodes were excised with cutadapt using forward and reverse NGS primer sequences^86,87^. Then, barcodes were dereplicated and tabulated using SeqFu, and the results were combined into a single table of barcode occurrences by experimental condition^90^. After counting, any barcodes that were not mapped to effector pairs via nanopore were discarded, leaving an average of 76.6% of NGS reads (Supplementary Fig. 6e), and any barcodes containing homopolymer repeats of 8 or more were discarded due to limitations of nanopore sequencing. Next, barcode counts mapping to the same effector pair were combined to produce a total read count for each bivalent effector in each condition. Effector pairs were considered dropouts if there were less than 100 cumulative reads in both bins in either replicate and/or less than 5 reads in any bin of either replicate to prevent artifactual inflation or deflation of enrichment scores. Then read counts were normalized by the sum of each bin, and the enrichment scores were calculated as the ratio of the normalized counts in the High bin versus the Low bin. The average enrichment score from both replicates was computed using a geometric mean of the High/Low enrichment ratio.

### Barcode-level enrichment analysis for Library 2

Read counts for individual barcodes across both replicates and sorting bins (HIGH and LOW) were treated as separate observations. To reduce noise from low-coverage barcodes, we applied a minimum threshold requiring the sum of reads across all bins and replicates to be at least 10 reads per barcode. For barcodes passing this filter, pseudocounts of +1 were added. Read counts were then normalized by the sum of reads in each bin, and enrichment ratios High/Low were calculated for each barcode in each replicate independently. For each effector domain pair, all log₂-transformed enrichment ratios from both replicates were aggregated. The mean and standard deviation of these log₂ ratios were calculated to obtain barcode-level average enrichment scores and their associated variability (Supplementary Data 4). For each effector pair, a two-sided Wilcoxon signed-rank test was performed comparing the distribution of barcode-level log₂ enrichment ratios against the mode of the distribution of all enrichment scores. *P* values were corrected for multiple testing using the Benjamini-Hochberg false discovery rate (FDR) procedure to obtain *q* values.

### Secondary validation of individual and bivalent effectors from Library 2

Selected individual and bivalent effectors from Library 2 were individually assessed via transient transfection into the PB Tet-On dCas9 K562 cell line with the 3X *CD81* guide array (for assessing activity on *CD81*) or without the 3X *CD81* guide array (for assessing activity on additional genes). To clone the constructs for transient transfection, a CcdB dropout landing pad was first made to assemble the pTRE3G-MCP-XTEN80-effector fusion while also expressing pEF1a-EGFP-P2A-PuroR in a divergent orientation (Supplementary Data 9, Plasmids, pMH308). The individual clonal effectors ordered from Twist Biosciences were directly used for insertion into the Effector 2 position via the BsmBI sites in the flanking sequences. To generate the proper overhangs for insertion into the Effector 1 position, clonal effectors were predigested with BsaI (600 ng clonal fragment, 1 μL BsaI-HFv2 (NEB, R3733L), 1 μL Quick CIP (NEB, M0525S), 1 μL 10X rCutSmart buffer (NEB, B6004S), and H_2_O to 10 μL incubated at 37 °C for 2 hours and 80 °C for 1 hour). The same XTEN16 linker was used for bivalent assembly, while an Effector 2 stuffer was used in place of both Effector 2 and the XTEN16 linker for monovalent assembly (Supplementary Data 9, Misc.). To prepare the XTEN16 linker and Effector 2 stuffer, individual ssDNA oligos were ordered, annealed, and phosphorylated (1 μL 100 μM F oligo, 1 μL 100 μM R oligo, 1 μL T4 ligase buffer (NEB, B0202S), 1 μL T4 PNK (NEB, M0201S), and H_2_O to 6 μL incubated at 37 °C for 30 min, 65 °C for 20 min, and 95 °C for 5 min before cooling down to 4 °C over 15 min). The final assembly of bivalent effectors was performed using Golden Gate assembly (50 ng pMH308, 0.5 μL EF1 predigest reaction, 0.2 μL XTEN16 oligo anneal, 0.5 μL EF2 clonal fragment at ~60 ng/μL, 1 μL T4 ligase buffer (NEB, B0202S), 0.5 μL Esp3I (Thermo Fisher Scientific, FD0454), 0.5 μL T4 ligase (NEB, R0202L), and H_2_O to 10 μL incubated at 37 °C for 5 min, followed by 30 cycles of 37 °C for 5 min and 16 °C for 10 min, followed by final ligation at 16 °C for 20 min, final digestion at 37 °C for 30 min, and heat inactivation at 80 °C for 20 min). The final assembly of monovalent effectors was also performed using golden gate assembly (50 ng pMH308, 0.5 μL EF1 predigest reaction, 0.2 μL EF2 stuffer oligo anneal, 1 μL T4 ligase buffer (NEB, B0202S), 0.5 μL Esp3I (Thermo Scientific, FD0454), 0.5 μL T4 ligase (NEB, R0202L), and H_2_O to 10 μL incubated at 37 °C for 5 min, followed by 30 cycles of 37 °C for 5 min and 16 °C for 10 min, followed by final ligation at 16 °C for 20 min, final digestion at 37 °C for 30 min, and heat inactivation at 80 °C for 20 min). 2 μL of each reaction were transformed into MACH1 cells (Thermo Fisher Scientific) before individual colonies were picked and grown overnight in 3 mL TB for plasmid purification with NucleoSpin Plasmid Transfection-grade miniprep kit (Machery-Nagel, 740490). All plasmids were sequence confirmed via full plasmid nanopore sequencing.

Catalytic mutations for specific effectors were sourced through literature review (Supplementary Data 5). To clone mutations into sequence confirmed individual validation constructs generated above, 2-3 PCR fragments were made to overlap over the mutation site or sites and the GFP gene for Gibson assembly of the mutant plasmids (Supplementary Data 9, Primers, MH302-MH345). PCR fragments were generated from corresponding unmutated plasmids (10 ng parent plasmid, 0.75 μL 10 μM F primer, 0.75 μL 10 μM R primer, 12.5 μL KAPA HiFi HotStart ReadyMix (Roche, KK2601), H_2_O to 10 μL incubated at 95 °C for 3 min, 18 cycles of 98 °C for 20 sec and 72 °C for 3 min, and 72 °C for 6 min, followed by addition of 1 μL DpnI (NEB, R0176S) and incubation at 37 °C for 1 hour and 80 °C for 20 min). 2 μL of the resulting reactions were analyzed for product length and purity via gel electrophoresis before mutant plasmids were assembled via Gibson assembly (1 μL each corresponding PCR reaction, 10 μL 2X Gibson MM, and H_2_O to 10 μL incubated at 50 °C for 1 hour). 2 μL of each reaction were transformed into MACH1 cells (Thermo Fisher Scientific) before individual colonies were picked and grown overnight in 3 mL TB for plasmid purification with NucleoSpin Plasmid Transfection-grade miniprep kit (Machery-Nagel, 740490). All mutant plasmids were sequence confirmed via full plasmid nanopore sequencing.

Sequence confirmed validation plasmids were nucleofected into the PB Tet-On dCas9 K562 cell lines using SF Cell Line 96-well Nucleofector kit (Lonza, V4SC-2096). The manufacturer’s instructions were followed using 800 ng plasmid DNA per nucleofection. For testing effectors on *CD81*, only the effector plasmid was nucleofected into the Tet-On dCas9 cell line with the 3X *CD81* guide array. For testing effectors on additional genes, the effector plasmid and the corresponding 3X guide array plasmid were nucleofected at a 1:1 mass ratio into the Tet-On dCas9 cell line without the 3X *CD81* guide array. After nucleofection, cells were plated directly into 1 μg/mL final concentration of doxycycline to induce dCas9 and MCP-effector expression. One day after nucleofection and every subsequent day until 5 days post-nucleofection, media was replaced with fresh media containing 1 μg/mL doxycycline and 2 μg/mL puromycin (Gibco, A1113803). Five days post-nucleofection, cells were analyzed for transient effector activity on the gene target by transferring to 96 well U-bottom plates and spinning down at 300 g for 3 min. Media was aspirated, and cells were resuspended in 100 μL stain buffer (BD, 554656) per well. Then 100 μL stain buffer containing 1.25 μL fluorescently conjugated antibody was added to each well and mixed by pipetting before incubation at 4 °C for 30 min (APC CD55 antibody (Biolegends, 311311), Alexa Fluor 647 CD58 antibody (BD Pharmingen, 563567), PE CD81 antibody (Invitrogen, MA1-10292), APC CD151 antibody (Biolegends, 350405), and APC CD155 antibody (eBioscience, 2H7CD155). After incubation, cells were washed twice with 200 μL stain buffer before final resuspension in 200 μL stain buffer per well. Stained cells were analyzed by flow cytometry on an Attune NxT Flow Cytometer (Thermo Fisher Scientific). To assess for long-term effector activity, cells at five days post-nucleofection were transferred to 96 well V-bottom plates and washed twice with 200 μL DPBS (Gibco, 14190144) per well before resuspending in fresh media without doxycycline or puromycin. Three days later and at subsequent timepoints, cells were split to maintain growth, and the remaining cells were stained and analyzed as above. To measure the doubling time of cells during the memory phase, cell density was determined at three days post-doxycyline washout and 24 hours later by using CellTiter-Glo Cell Viability Assay (Promega G7571) and comparing to a standard curve of WT K562 cells. The change in cell density was then used to calculate the doubling time for each condition.

To systematically analyze large batches of flow cytometry data, Cytoflow’s Jupyter notebook integration was used^91^. Live and singlet gates were first defined on WT K562 cells measured using the same cytometer settings, and the gates were applied to each sample file. Then, for transient timepoint analysis, cells were further gated for BFP and GFP, indicating dCas9 expression and the presence of the MCP-effector plasmid. Transient timepoints on genes other than *CD81* were also gated on mCherry, indicating the presence of the 3X guide array plasmid. Threshold gates were defined at the 99.9th percentile of the channel measurements in WT K562 cells. For memory timepoints, no gating beyond live and singlet gates was performed. After gating, mean fluorescence intensity (MFI) was calculated for each sample using the flow.geom_mean function in Cytoflow on the channel corresponding to the fluorescent antibody. Percent change in MFI was calculated using the MFI from the neutral DMD control as a reference. Percent cells activated and percent cells repressed were calculated using threshold gating at the 1st and 99th percentile of the neutral DMD control effector, and the percent cells silenced was calculated using threshold gating at the 99th percentile of unstained WT K562 cells.

### Analysis of individual and bivalent effector strength

A marginal effect test was defined to assess the marginal, or average, effect of a given individual effector. To conduct the test for a given effector, the bivalent pairs and corresponding average fold enrichment scores were split into two groups, those containing the effector and those not containing the effector. The marginal score was defined as the difference in mean fold enrichment between the two groups, and if ≥ 30 pairs were present in both groups, a Welch’s t-test was conducted. It was determined that a difference of means t-test was necessary, rather than a one sample t-test, to correct for library composition bias. In other words, the mean fold enrichment of any given individual effector would naturally be positive or negative if the overall library had a bias in either direction simply because the given effector is paired with every other library member. A Welch’s t-test was chosen as opposed to a Student’s t-test because it was expected that individual effectors may vary in their interactions with other library members, thus not only altering the mean fold enrichment but also the standard deviation of fold enrichment scores. Once all the effectors were analyzed, *p* values were corrected for a false discovery rate of 5%.

### Synergy scoring to identify synergistic and antagonistic combinations

To identify bivalent combinations with unexpected activities, synergy scores were calculated for each bivalent combination based on the expected effects of each individual effector (Fig. 4a). First, the individual effects of each effector were calculated using our marginal score metric, with the difference that each effector was scored for both the N-terminal and C-terminal position. Then, the sum of the corresponding marginal scores for each bivalent combination was computed. Because effectors could still be additive in nature even if the measured score did not exactly match the sum, a range for additive activity was defined that assumes that effectors may not contribute their full marginal strength to the combination but that the contribution of each effector should still be in the expected direction. For expected repressors, the lower bound of the additive range was defined as the minimum of the N-terminal marginal score, the C-terminal marginal score, and the sum of the marginal scores (min(N-term, C-term, Sum)), and the upper bound of the range was defined as the maximum of the sum of the marginal scores and the minimum of the two marginal scores (max(Sum, min(N-term, C-term))). For expected activators, the expressions are reversed and the additive range becomes (min(Sum, max(N-term, C-term))) to (max(N-term, C-term, Sum)). Log_2_ fold enrichment scores from HTS data were first mean centered, a logical step considering that marginal scores are relative contributions and are unaffected by central tendency, and subsequently compared to the additive range. In all cases, if the observed data fell within the additive range, the pair was considered additive and assigned a synergy score of 0. For expected repressors, bivalent combinations were considered “synergistic” if the measured score fell below the additive range, i.e., it was more repressive than expected, and the combination was considered “antagonistic” if the measured score fell above the additive range, i.e., it was less repressive than expected. Scores for expected activators were determined in the same way but in the opposite direction, with synergistic combinations exhibiting unexpectedly high observed scores and antagonistic combinations exhibiting unexpectedly low observed scores. In both cases, the synergy score was defined as how far outside the additive range the observed score fell, being positive for synergy and negative for antagonism. Because Library 1 was entirely composed of expected repressors, every combination was scored as a repressor. For Library 2, the sum of the marginal scores determined whether the combination was scored as an activator or a repressor with positive sums being scored as expected activators and negative sums being scored as expected repressors.

To test if individual effectors broadly synergize or antagonize with KRAB members from Library 1 or HDAC members from Library 2, the synergy scores from all combinations containing the given effector with a KRAB or HDAC member were tested against the null hypothesis that the average synergy score was zero using a two-sided one-sample Wilcoxon test. Only KRAB or HDAC members with marginal scores less than −0.5 were considered, and *p* values were subsequently FDR corrected for multiple hypothesis testing. In addition to performing significance testing, the average synergy score and the average log_2_ fold enrichment score for the combinations were calculated.

### Calculation of residual-based synergy scores using homotypic pairs

In addition to the original marginal score-based synergy calculations, we developed an alternative residual-based approach using measured homotypic pair (homodimer) values as the baseline expectation (Supplementary Fig. 13). All Log₂(HIGH/LOW) enrichment scores were first centered by subtracting the mode of the distribution, calculated by identifying the peak of a 50-bin histogram of all enrichment values. For each heterodimer A-B, the expected enrichment was calculated as the average value of the two corresponding mode-corrected homodimer measurements: Expected = (A-A + B-B)/2, where A-A and B-B represent the measured enrichment scores of the respective homodimers. We performed linear regression of observed enrichment scores against expected values (Observed ~ Expected) across all heterodimer pairs with available homodimer measurements. The residual for each pair was calculated as: Residual = Observed - Predicted, where Predicted is the value from the regression line. To ensure consistent biological interpretation of synergy across the full range of enrichment values, we applied context-dependent sign adjustments. For Library 1, which consists predominantly of transcriptional repressors with negative enrichment scores, all residuals were multiplied by −1 to maintain the convention that positive synergy scores indicate stronger-than-expected effects (more repression than expected from individual repressors). For Library 2, which contains both repressors and activators spanning positive and negative enrichment values, we applied conditional sign inversion: residuals for pairs with negative expected values (Expected <0) were multiplied by −1, while residuals for pairs with positive expected values (Expected > 0) remained unchanged. For Library 2, we classified data points as “within distribution” or “outside distribution” based on their deviation from the expected linear relationship. Using high-confidence pairs (Day 0: |sum of marginal scores | ≥ 0.3 and |average of homodimer enrichment scores | ≥ 1; Day 12: |sum of marginal scores | ≥ 0.15 and |average of homodimer enrichment scores | ≥ 0.5) as a reference population, we fitted a regression line and calculated the standard deviation (σ) of residuals from this line. Points were classified as “within distribution” if their distance from this regression line was ≤ 4.5σ, and “outside distribution” otherwise. This threshold was chosen to capture the main correlation pattern while identifying outliers.

### Comparison between stdMCP-KRAB and stdMCP-KRAB-L3MBTL3

pTRE3G-PuroR-T2A-stdMCP-XTEN80-KRAB(ZNF10, ZIM3, ZNF705)-L3MBTL3-WPRE and pTRE3G-PuroR-T2A-stdMCP-XTEN80-KRAB(ZNF10, ZIM3, ZNF705)-WPRE were infected by lentivirus to Tet-On dCas9 K562 (without *CD81* guide array) in MOI of less than 0.4.

For comparison across different panels of surface marker genes and guides, lentivirus encoding both sgRNA and mCherry marker were infected to both cell lines. Doxycycline concentration was maintained at 1-2 µg/mL since the LV-sgRNA infection. 5 days after lentiviral infection of sgRNAs, cells were stained with corresponding antibodies (APC anti-human CD55 antibody (Cat# 311311, Biolegends), APC Mouse Anti-Human CD81 antibody (Cat# 561958, BD Bioscience), and APC anti-human CD151 antibody (Cat# 350405, Biolegends)) and expression level was measured by Attune NxT Flow Cytometer (Thermo Fisher Scientific).

For *CD55*/*CD81*/*CD151* promoter tiling screens, the 2 kb sequence tiling −1000 bp to +999 bp region around the TSS of MANE Select transcript (hg38 genomic coordinates: *CD55* chr1:207320678-207322677, *CD81* chr11:2376310-2378309, *CD151* chr11:831952-833951) were used as inputs for CRISPick^79^ (Human GRCh38 (Ensemble v.111), CRISPRi, SpyoCas9, Hsu (2013) tracrRNA, Library mode, No-Site Controls = 25, Intergenic Controls = 25, CRISPick Quota = 1000, Do not allow picking guides with MAX off-target matches). The first letter of sgRNA sequence was replaced with guanosine (G) for efficient transcription by the U6 promoter. Oligo pools were synthesized (Twist Bioscience) after appending 65 bp 5′ stuffer and 64 bp 3′ stuffer sequences which contain primer binding sites for PCR and Esp3I binding sites for golden gate assembly.

Oligo pools were resuspended in TE buffer with the final concentration of 10 ng/µL. After resuspension, oligo pools were PCR amplified with the following protocol. For total volume of 50 µL PCR reaction, 0.5 µL oligo pool (10 ng/µL), 25 µL NEBNext High-Fidelity 2X PCR Master Mix (NEB), and a final concentration of 0.5 μM for each forward and reverse primers were prepared and mixed on ice. The thermocycling protocol was 1 cycle of 98 °C for 3 min, 27 cycles of 98 °C for 10 sec, 70 °C for 30 sec, 72 °C for 30 sec, and final 1 cycle of 72 °C for 2 min. After thermocycling, 36 µL PCR reactions were run through 9 lanes in 1% E-Gel EX Agarose Gel (Thermo Fisher Scientific). Bands with expected sizes were excised, and PCR products were extracted using Monarch DNA Gel Extraction Kit (NEB). Lentiviral backbone plasmids for MS2 sgRNAs were simultaneously digested and dephosphorylated with the following protocol. For 20 µL reaction, 2 µg of backbone plasmid, 2 µL FastDigest Esp3I (Thermo Fisher Scientific), 2 µL FastAP Thermosensitive Alkaline Phosphatase (Thermo Fisher Scientific), 4 µL 10X FastDigest Buffer, and water were mixed well. The thermocycling protocol was 37 °C for 1 hour and 70 °C for 5 min. Digested backbone was gel-extracted using Monarch DNA Gel Extraction Kit (NEB). Gel-extracted insert and backbone were ligated using the following golden gate reaction. For a total volume of 20 µL, 1 µL backbone, 1 µL insert, 1 µL FastDigest Esp3I (Thermo Fisher Scientific), 1 µL T4 DNA Ligase (40000 U/mL), 2 µL 10X T4 Ligase Buffer, and 14 µL water were mixed well. The thermocycling protocol was 5 min at 37 °C, 30 cycles of 5 min at 37 °C and 10 min at 16 °C, 20 min at 16 °C, 30 min at 37 °C, 20 min at 75 °C, and hold at 4 °C. The golden gate reaction was purified with DNA Clean & Concentrator-5 (Zymo) and eluted in 6 µL nuclease-free water. 1 µL of purified DNA was transformed into 25 µL Endura electrocompetent cells (Lucigen) following the manufacturer’s instructions. Colony coverage was at least 100X for each library.

For the dual-directional cell lines (mentioned in the dual-directional perturbation “Method” section below; stdMCP-KRAB with stdPCP-p65-HSF1 and stdMCP-KRAB-L3MBTL3 with stdPCP-p65-HSF1), on Day 0, 1.5 million cells were infected with 125 μL of lentivirus per replicate. On Day 5 post-infection, 8 million cells were stained with corresponding antibodies (APC anti-human CD55 antibody (Cat# 311311, BioLegend), APC Mouse Anti-Human CD81 antibody (Cat# 561958, BD Biosciences), and APC anti-human CD151 antibody (Cat# 350405, BioLegend)) in eBioscience Flow Cytometry Staining Buffer (Invitrogen). mCherry positive cells were sorted into ~30% high and low expression bins for each corresponding target gene by FACS. At least 0.125 million cells were collected for each bin. Genomic DNA was harvested using the NucleoSpin Blood kit (Macherey-Nagel). Enriched guide RNA sequences were PCR amplified from the gDNA using the following conditions. For one 50 μL PCR reaction, 25 μL of 2X NEBNext Ultra II Q5 Master Mix (NEB), 1 μg of gDNA, and a final concentration of 0.5 μM for each forward and reverse NGS primers were prepared and mixed on ice. The thermocycling protocol was 1 cycle of 98 °C for 30 sec, 27 cycles of 98 °C for 10 sec, 58 °C for 30 sec, 72 °C for 20 sec, and final 1 cycle of 72 °C for 2 min. After thermocycling, PCR reactions were pooled and run through 1% E-Gel EX Agarose Gel (Thermo Fisher Scientific). PCR products were extracted using Monarch DNA Gel Extraction Kit (NEB). Based on the quantification with the Qubit HS kit (Thermo Fisher Scientific), the library was sequenced with NextSeq 2000 with a P1 kit 300 cycles (Illumina) in paired-end sequencing mode.

Paired-end reads were merged using fastp with default parameters^86^. Merged reads were filtered by length (155–170 bp) using seqkit^92^. sgRNA sequences were counted from filtered reads using sgcount (https://github.com/noamteyssier/sgcount). For each sgRNA, read counts were normalized to total read counts per sample to obtain read fractions. Repression scores were calculated as the ratio of read fractions between low and high expression bins (LOW/HIGH) for each replicate. Repression scores were normalized by dividing the repression score of each targeting sgRNA by the average repression score of non-target sgRNAs, such that non-target sgRNAs have a baseline value of 1.

For comparing *NF1* gene mRNA knockdown levels, cells were nucleofected with plasmids encoding sgRNA (Supplementary Data 10). Doxycycline was maintained at 1-2 µg/mL starting one day before nucleofection and continuing until qPCR measurement. Two days post-nucleofection, cells were lysed and *NF1* mRNA levels were measured following a previous protocol^82^.

For comparing AQR gene expression levels, stable K562 cell lines expressing either stdMCP-KRAB or stdMCP-KRAB-L3MBTL3 from a TRE3G promoter were infected with lentivirus encoding both AQR-targeting sgRNA and mCherry in doxycycline-free media (Supplementary Data 9). 3 days after lentiviral infection, mCherry positive cells were sorted. After recovery in doxycycline-free media for 1 day, induction with 1 μg/mL doxycycline started. AQR mRNA levels were measured by qRT-PCR 2 days after doxycycline induction.

After removing cell culture media and washing with calcium- and magnesium-free DPBS (Gibco), cells were treated with 50 µL of Complete RNA Lysis Buffer (9.6 mM Tris–HCl (pH 7.8), 0.5 mM MgCl_2_, 0.44 mM CaCl_2_, 10 μM DTT, 0.1% (wt/vol) Triton X-114, and 300 U/mL DNAse I, 6 U/mL Proteinase K) and incubated at room temperature for 8 minutes in 96-well tissue culture plates. For lysis termination, 3 µL of RNA lysis stop solution (1 mM Proteinase K inhibitor, 90 mM EGTA, and 113 μM DTT in UltraPure water) was pre-aliquoted into a 96-well PCR plate. Following thorough pipetting for the lysis, 30 µL of the lysed cell mixture was added to the 3 µL stop solution and incubated for 3 minutes. The reverse transcription was performed by combining 8 µL of the terminated lysis mixture with 32 µL of RevertAid RT Reverse Transcription Kit (Thermo Fisher Scientific) mixture containing random hexamer primers, prepared according to manufacturer’s protocol. The reverse transcription reaction underwent thermal cycling at 25 °C for 10 minutes, followed by 37 °C for 60 minutes, and 95 °C for 5 minutes, after which the resulting cDNA was either used immediately or stored at −80 °C.

The qPCR reaction mixture consisted of 12 µL of 2X TaqMan Fast Advanced Master Mix (Thermo Fisher Scientific), 1.2 µL 20X FAM TaqMan Gene Expression Assays for the target gene *NF1* (Thermo Fisher Scientific, Hs01035108_m1) or AQR (Thermo Fisher Scientific, Hs00390757_m1), 1.2 µL 20X VIC Human GAPD (GAPDH) Endogenous Control (Thermo Fisher Scientific, 4310884E), and 9.6 µL of cDNA, totaling 24 µL per reaction. Three 5-µL technical replicates of this qPCR master mix were aliquoted into a 384-well optical plate. The qPCR cycling conditions included initial polymerase activation at 95 °C for 2 minutes, followed by 50 cycles of denaturation at 95 °C for 1 second and annealing/extension at 60 °C for 20 seconds. Following qPCR completion, the target gene expression fold change relative to control was calculated using the ddCt method.

For the dual-directional perturbations using the MS2/PP7 system, pTREtight-stdPCP-p65-HSF1-T2A-BFP were lentivirally infected to the previous two cell lines described above. pTREtight-stdPCP-p65-HSF1-T2A-BFP was cloned from the construct pHR-CMV-stdPCP-p65-HSF1-T2A-GFP (Addgene #199456)^64^. The same amounts of lentivirus encoding both mouse U6 promoter-driven 2x MS2 sgRNA and human U6 promoter-driven 2x PP7 sgRNA were infected to both cell lines. Doxycycline concentration was maintained at 1-2 µg/mL since the day of LV-dual MS2/PP7 sgRNAs infection. CD81/CD274 expression profiles were measured 5 days post-infection of dual MS2/PP7 sgRNAs following staining with antibodies against both targets (APC Mouse Anti-Human CD81 antibody (Cat# 561958, BD Bioscience) and Alexa Fluor 488 Mouse Anti-Human CD274 antibody (Cat# 53-5983-42, Invitrogen)) by Attune NxT Flow Cytometer (Thermo Fisher Scientific).

For growth measurement of the KRAB + L3MBTL3 combination, an mCherry-positive parental cell line was first generated by infecting the Tet-On dCas9 K562 cell line (without 3X guide arrays) with pSV40-mCherry-WPRE lentivirus, followed by sorting for mCherry-positive cells using FACSAria Fusion (BD Bioscience). After 2 days of recovery, mCherry-positive cells were mixed at ~50:50 ratio with either the parental cell line without mCherry or the previously generated cells expressing pTRE3G-PuroR-T2A-stdMCP-XTEN80-KRAB(ZNF10)-L3MBTL3-WPRE in 3 independent replicates. Following mixing, cells were induced with doxycycline and passaged in 96-well plates, with mCherry-positive proportions measured at multiple timepoints using an Attune NxT Flow Cytometer (Thermo Fisher Scientific).

A comparison between dCas9-KRAB and L3MBTL3-dCas9-KRAB in Ngn2-induced neurons from human embryonic stem cells was performed on the H1 cell line (male, WiCell (Cat # WA01)). Cells were cultured in a humidified incubator at 37 °C and 5% CO_2_, in mTeSR™ Plus media (STEMCELL Technologies) on Cultrex-coated (R&D Systems, 343400502) 6-well plates.

To generate PB-dCas9 H1 cell lines expressing dCas9-KRAB and L3MBTL3-dCas9-KRAB, dCas9 fusions were cloned into a PB vector under the control of the CAG promoter. BFP and Neomycin resistance cassettes were also included. Cells were transfected with 70 ng of plasmid and 30 ng of a separate PiggyBac transposase plasmid (System Biosciences) using Fugene HD (Promega Corporation), according to manufacturer recommendations. Two days following transfection, cells were selected with 100 μg/mL G418 (10131027, Gibco). After selection, cells were expanded and assessed for BFP expression. Cells were then differentiated into neurons to assess activity of dCas9-KRAB and L3MBTL3-dCas9-KRAB.

To differentiate H1 lines into neurons, cells were split with accutase and transduced in suspension with lentivirus expressing Tet-on Ngn2-P2A-puromycin resistance and rtTA under the control of the Ubiquitin promoter (UbC). Lentiblast was used to boost transduction efficiency, according to manufacturer recommendations. Cells were plated into Cultrex (R&D Systems 343400502)-coated 6-well plates with mTeSR media (StemCell Technologies) containing ROCK inhibitor Y-27632 (10 µM, Abcam). Four days later, cells were transduced as described before with lentivirus expressing a non-targeting gRNA or gRNAs targeting *CD81* (Supplementary Data 9).

When cells reached 70% confluency, they were split with Accutase and re-plated into Cultrex-coated 24-well plates with mTeSR media containing ROCK inhibitor Y-27632. The following day (Day 0), cells were induced for Ngn2 using 2 µg/mL Doxycycline (Sigma Aldrich D3072). On Day 1, media was replaced with neural induction media, containing DMEM/F12 (Gibco 11330032) supplemented with Doxycycline (2 µg/mL, Sigma D3072), Insulin (5 µg/mL, Roche 11376497001), BSA (10 mg/mL, Sigma A4161), Apo-transferrin (10 mg/mL, Sigma T1147), Putrescine (1.6 mg/mL, Sigma P57800), Progesterone (0.00625 mg/mL, Sigma P8783), Sodium selenite (0.00104 mg/mL, S5261), BDNF (10 µg/mL, Peprotech 450-02), and Puromycin (5 µg/mL, Life Technologies A1113803)). Media was changed daily. After 2 days of puromycin selection, cells were passaged with Accutase and replated in Matrigel (Corning 354230)-coated-96 well plates in neuronal maturation media containing Neurobasal Media (Gibco 21103049), DMEM Media (Gibco 10569010), HEPES (0.5x, Gibco 15630130), Glutamax (1 mM, Gibco 35050061), Doxycycline (2 µg/mL, Sigma D3072), BDNF (10 µg/mL, Peprotech 450-02), dbCAMP (49.14 µg/mL, Sigma Aldrich D0627), B27 with vitamin A (1x, Gibco 17504044), N-acetyl cysteine (5 µg/mL, Sigma A9165). On Day 4, media was replaced with neural maturation media containing AraC (2.4 µg/mL, Sigma Aldrich C1768) to remove any proliferating cells from the culture. On Day 5, human primary astrocytes (Sciencell 1800-10) were added to the cultures to support neuronal survival. Cells were maintained in Co-culture media, containing Neurobasal Media (Gibco 21103049), DMEM Media (Gibco 10569010), HEPES (0.5x, Gibco 15630130), Glutamax (1 mM, Gibco 35050061), Doxycycline (2 µg/mL, Sigma D3072), BSA (10 mg/mL, Sigma A4161), Apo-transferrin (10 mg/mL, Sigma T1147), Putrescine (1.6 mg/mL, Sigma P57800), Progesterone (0.00625 mg/mL, Sigma P8783), Sodium selenite (0.00104 mg/mL, S5261), Insulin (5 ug/mL, Roche 11376497001), T3 (34 ng/ml, Sigma Aldrich T6397), dbCAMP (49.14 µg/mL, Sigma Aldrich D0627), hbEGF (10 ng/ml, Peprotech 100-47) and N-acetyl cysteine (5 µg/mL, Sigma A9165). Activity of dCas9-KRAB and L3MBTL3-dCas9-KRAB on *CD81* was tested at Day 14. Cells were detached with warm accutase and stained for CD81 (Becton Dickinson 551112) in warm stain buffer (Becton Dickinson 554656) supplemented with 20 mM glucose (Sigma G7021). Stained cells were analyzed by flow cytometry on a NovoCyte Quanteon Flow Cytometer System (Agilent). Cells were gated for BFP, indicating dCas9 expression, and GFP, reporting for sgRNA expression. Knockdown efficiency was calculated by normalizing APC-CD81-MFI in cells transduced with *CD81*-targeting guides to APC-CD81-MFI in cells transduced with non-targeting guides.

For the CRISPRi growth screen, 57 genes with varying degrees of essentiality score were chosen and 10 CRISPRi guide RNAs and 10 CRISPRa guide RNAs were generated by CRISPick^79^ ((NCBI RefSeq v.GCF_000001405.40-RS_2024_08), RS3seq-Hsu2013). In addition, 200 no-site control guide RNAs were generated using the CRISPick yielding a total of 1340 guide RNA sequences (Supplementary Data 8). The first nucleotide of sgRNA sequence was replaced with guanosine (G) for efficient transcription by the U6 promoter. Oligo pools were synthesized (Twist Bioscience) after appending 65 bp 5′ stuffer and 64 bp 3′ stuffer sequences which contain primer binding sites for PCR and Esp3I binding sites for golden gate assembly.

Oligo pools were resuspended in nuclease-free water with the final concentration of 10 ng/µL. After resuspension, oligo pools were PCR amplified with the following protocol. For total volume of 50 µL PCR reaction, 0.5 µL oligo pool (10 ng/µL), 25 µL NEBNext High-Fidelity 2X PCR Master Mix (NEB), and a final concentration of 0.5 μM for each forward and reverse primers were prepared and mixed on ice. The thermocycling protocol was 1 cycle of 98 °C for 3 min, 27 cycles of 98 °C for 10 sec, 70 °C for 30 sec, 72 °C for 30 sec, and the final 1 cycle of 72 °C for 2 min. After thermocycling, 36 µL PCR reactions were run through 9 lanes in 1% E-Gel EX Agarose Gel (Thermo Fisher Scientific). Bands with expected sizes were excised, and PCR products were extracted using Monarch DNA Gel Extraction Kit (NEB). Lentiviral backbones plasmids for MS2 sgRNAs were simultaneously digested and dephosphorylated with the following protocol. For 80 µL reaction, 4 µg of backbone plasmid, 4 µL FastDigest Esp3i (Thermo Fisher Scientific), 4 µL FastAP Thermosensitive Alkaline Phosphatase (Thermo Fisher Scientific), 8 µL FastDigest Buffer (10X), and water were mixed well. The thermocycling protocol was 37 °C for 1 hour and 70 °C for 5 min. The digested backbone was gel-extracted by Monarch DNA Gel Extraction Kit (NEB). Gel extracted guide RNA library and gel extracted backbone were ligated using the following golden gate reaction. For total volume of 100 uL golden gate reaction, 1 µg gel extracted backbone, 187.5 ng gel extracted library, 10 µL 10X T4 Ligase buffer, 10 µL T4 Ligase, 10 µL FastDigest Esp3i (Thermo Fisher Scientific), and water were mixed well. The thermocycling protocol was 1 cycle of 37 °C for 5 min, 30 cycles of 37 °C for 5 min, 16 °C for 5 min, final 5 min digestion at 37 °C, and 25 min heat-inactivation at 75 °C. The golden gate reaction was purified with DNA Clean & Concentrator-5 (Zymo) and was eluted in 6 μL of nuclease-free water. 1 μL of purified DNA was transformed into 25 μL of Endura electrocompetent cells (Lucigen) following the manufacturer’s instructions with 2 mL of prewarmed recovery media. 2 mL of cells were plated onto a 245 mm × 245 mm plate, and serially diluted cells were plated onto 100 mm diameter plates for the estimation of colony numbers. After overnight incubation at 30 °C, colonies were collected, and plasmid libraries were extracted with Midiprep (Machery-Nagel). Colony coverage was at least 667X for 1340 library members.

For two cell lines (stdMCP-KRAB, stdMCP-KRAB-L3MBTL3), 5 million cells were infected with 80 μL of lentivirus per replicate on Day 0, cells were kept cultured in growth media containing 1-2 μg/mL doxycycline. On Day 5, at least 1000X mCherry-positive cells were sorted for each condition. Cells were kept cultured at minimum 1000X coverage until Day 14.

On Day 14, genomic DNA was harvested using the NucleoSpin Blood kit (Machery-Nagel). Enriched guide RNA sequences were PCR amplified from the gDNA using the following conditions. For one 50 μL of the PCR reaction, 25 μL of 2X NEBNext Ultra II Q5 Master Mix (NEB), 1 μg of gDNA or 2 ng of plasmid library, and a final concentration of 0.5 μM for each forward and reverse NGS primers were prepared and mixed on ice. The thermocycling protocol was 1 cycle of 98 °C for 30 sec, 27 cycles of 98 °C for 10 sec, 62 °C for 30 sec, 72 °C for 20 sec, and final 1 cycle of 72 °C for 2 min. After thermocycling, PCR reactions were pooled and run through 1% E-Gel EX Agarose Gel (Thermo Fisher Scientific). PCR products were extracted using Monarch DNA Gel Extraction Kit (NEB). Based on the quantification with Qubit HS kit (Thermo Fisher Scientific), all experimental conditions and plasmid library were pooled with ~10% PhiX (Illumina) spike in. The library was sequenced with Miseq i100 with a 25 M kit 300 cycles (Illumina) in paired-end sequencing mode.

Paired-end Illumina sequencing reads (R1 and R2) were merged using fastp^86^. Merged reads were subsequently filtered by length to retain only sequences between 155-170 bp using seqkit with gap removal enabled (-g flag) to remove any gap characters introduced during processing^92^. Guide RNA sequences were extracted from the merged and filtered reads using a sliding window approach. Each 20-nucleotide subsequence within the merged reads was compared against the reference guide library using exact sequence matching. When a match was identified, the corresponding guide sequence was recorded and the search moved to the next read. The plasmid library sample served as the baseline reference for all comparative analyses and used for subsequent fold change calculations. Depletion scores were calculated using negative log2 fold change between experimental conditions and the plasmid baseline. A pseudocount of 1 was added to the frequency terms in the log2 fold change calculation to prevent division by zero. Only guides with 10 or more reads in the plasmid baseline were included in depletion analysis to ensure reliable fold change estimates. This filtering was applied to the raw plasmid counts before any normalization or pseudocount addition.

### Statistics & Reproducibility

Statistical analyses were performed as described in the relevant Methods subsections. No statistical method was used to predetermine sample size. No data were excluded from the analyses. The experiments were not randomized. The investigators were not blinded to allocation during experiments and outcome assessment.

### Reporting summary

Further information on research design is available in the Nature Portfolio Reporting Summary linked to this article.

## Supplementary information

Supplementary Information

Description of Additional Supplementary Files

Supplementary Data 1

Supplementary Data 2

Supplementary Data 3

Supplementary Data 4

Supplementary Data 5

Supplementary Data 6

Supplementary Data 7

Supplementary Data 8

Supplementary Data 9

Supplementary Data 10

Supplementary Data 11

Reporting Summary

Transparent Peer Review file

## Source data

Source Data

## Data Availability

Illumina and Nanopore sequencing datasets generated in this study have been deposited in the NCBI Sequence Read Archive under BioProject PRJNA1143488. Source data are provided as a Source Data file. All data are publicly available without restrictions. Source data are provided with this paper.

## Code availability

The computational approach is described in detail in the Methods. Code written to analyze high-throughput screening data is freely available under the Apache 2.0 License with no restrictions, at (https://github.com/hsulab-arc/COMBINE) and deposited on Zenodo under (10.5281/zenodo.19102406).
