# Supplementary material for: A combinatorial domain screening platform reveals epigenetic effector interactions for transcriptional perturbation
Source: Nat Commun. 2026 Apr 24;17:5697. doi: 10.1038/s41467-026-72227-9 (PMC13319239; doi:10.1038/s41467-026-72227-9)
Supplement: Supplementary file 1 — Supplementary Information [file 41467_2026_72227_MOESM1_ESM.pdf]

Supplementary Figures

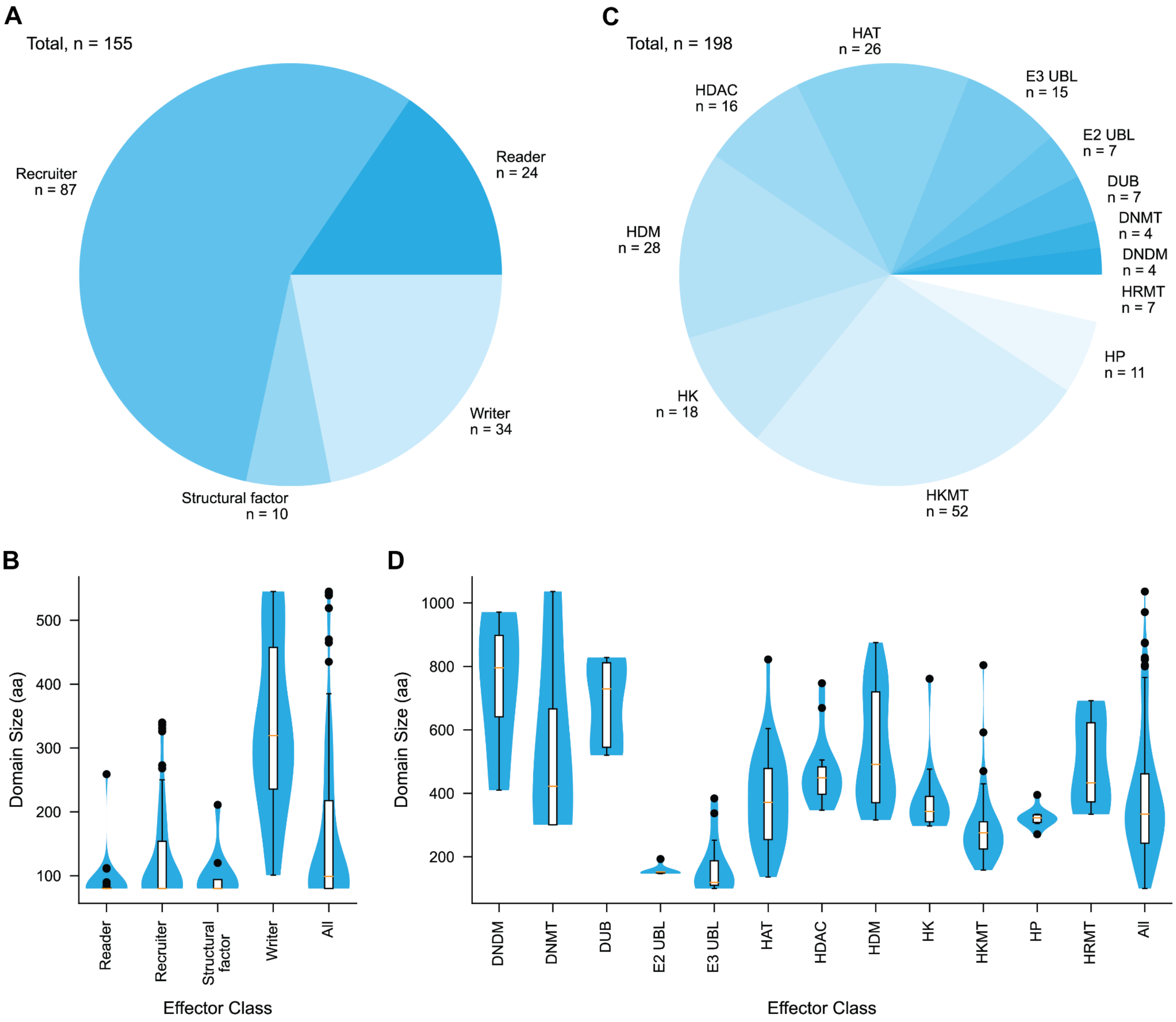

Supplementary Figure 1. Classifications and size distributions of individual domains from Library 1 and Library 2

(**A**) Classification of Library 1 members.

(**B**) Size distributions of specific classes from Library 1.

(**C**) Classification of Library 2 members. DNA demethylation machinery (DNDM), DNA methyltransferase (DNMT), E2 and E3 ubiquitin ligases (UBL), histone acetyltransferase (HAT), histone arginine methyltransferase (HRMT), histone deacetylase (HDAC), histone demethylase (HDM), histone deubiquitinase (DUB), histone kinase (HK), histone lysine methyltransferase (HKMT), and histone phosphatase (HP).

(**D**) Size distributions of specific classes from Library 2.

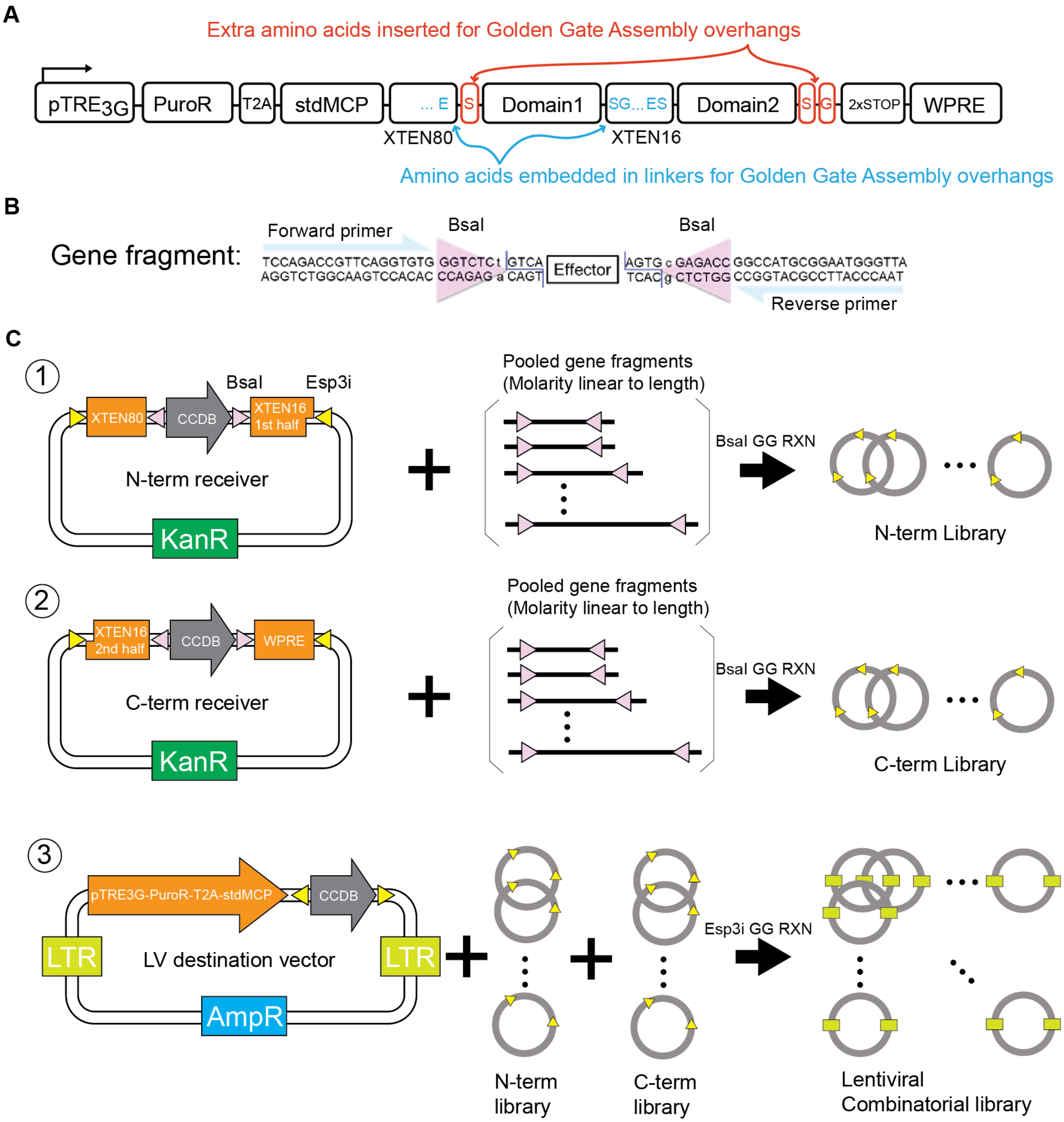

Supplementary Figure 2. Library 1 combinatorial cloning strategy

(**A**) Schematic of combinatorial effector candidate cassette in lentiviral vector for HTS of Library 1. DNA sequence encoding for Glutamic acid (E) – Serine (S) serves as a 5` overhang for the golden gate cloning. DNA sequence encoding for Serine (S) – Glycine (G) serves as a 3` overhang for the golden gate cloning. Cyan amino acid symbols indicate amino acids already encoded in the XTEN linker CDS. Orange amino acid symbols indicate amino acids inserted for the golden gate cloning overhang.

(**B**) Schematic of gene fragments ordered for Library 1 (**Supplementary Data 1**). Although we included PCR amplification handles in the design, it was not necessary to PCR amplify the gene fragments for cloning.

(**C**) Library cloning procedure for the generation of Library 1 bivalent domain candidates. The procedure consists of three sequential pooled Golden Gate assemblies. The first Golden Gate reaction involves the library cloning of pooled gene fragments into the N-term/KanR1 receiver (pCM001). Each gene fragment was pooled with a molar ratio linear to its size including the PCR amplification handles. For example, we added 1.5-fold of the 450 bp gene fragment compared to the 300 bp gene fragment. The second Golden Gate reaction involves the library cloning of pooled gene fragments into the C-term/KanR2 receiver (pCM002). The final Golden Gate reaction involves the library cloning of the N-terminus library and C-terminus library into the LV_AmpR_backbone (pCM003).

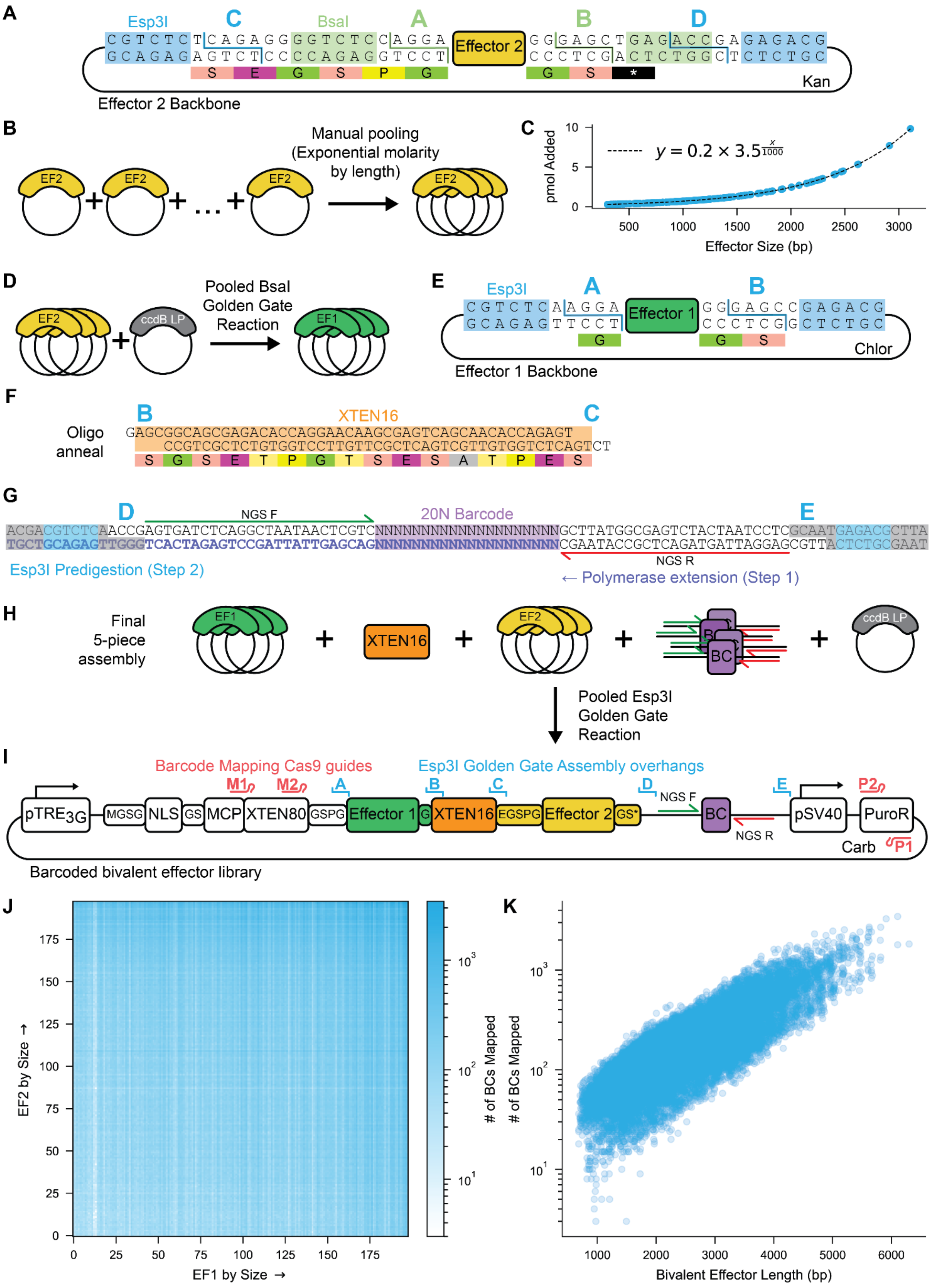
A
Esp3I
C
BsaI
A
B
D
Effector 2
Effector 2 Backbone
Kan
B
Manual pooling
(Exponential molarity
by length)
EF2
C
$y = 0.2 \times 3.5^{\frac{x}{1000}}$
pmol Added
Effector Size (bp)
D
Pooled BsaI
Golden Gate
Reaction
EF2
ccdB LP
EF1
E
Esp3I
A
B
Effector 1
Effector 1 Backbone
Chlor
F
Oligo
anneal
B
XTEN16
C
GAGCGGCAGCGAGACACCAGGAACAAGCGAGTCAGCAACACCAGAGT
CCGTCGCTCTGTGGTCCTTGTTCGCTCAGTCGTTGTGGTCTCAGTCT
G
D
NGS F
20N Barcode
E
NGS R
Esp3I Predigestion (Step 2)
← Polymerase extension (Step 1)
H
Final
5-piece
assembly
EF1
XTEN16
EF2
BC
ccdB LP
Pooled Esp3I
Golden Gate
Reaction
I
Barcode Mapping Cas9 guides
Esp3I Golden Gate Assembly overhangs
M1
M2
P2
P1
pTRE3G
MGSG
NLS
GS
MCP
XTEN80
GSPG
Effector 1
XTEN16
EGSPG
Effector 2
GS*
NGS F
BC
NGS R
pSV40
PuroR
Carb
Barcoded bivalent effector library
J
EF2 by Size →
EF1 by Size →
# of BCs Mapped
K
# of BCs Mapped
Bivalent Effector Length (bp)

Supplementary Figure 3. Library 2 combinatorial cloning and barcode mapping strategy

(**A**) Schematic of clonal gene fragments ordered for Library 2 with dual golden gate overhangs.
(**B**) Clonal fragments of individual effectors were pooled manually to form the Effector 2 pool.
(**C**) The amount of plasmid DNA added per effector increased 3.5-fold per kb.
(**D**) The first golden gate assembly reaction for cloning effectors from Effector 2 pool into Effector 1 landing pad plasmid.
(**E**) Schematic of cloned Effector 1 plasmid with golden gate overhangs.
(**F**) XTEN16 linker was ordered as ssDNA and annealed with proper golden gate overhangs.
(**G**) Barcodes were ordered as a ssDNA fragment containing a 20N sequence. Polymerase extension was used to fill in barcodes, forming a dsDNA fragment, before the fragment was predigested with Esp3I to form proper golden gate overhangs.
(**H**) Final 5-piece golden gate reaction for generating Library 2 bivalent effectors for HTS.
(**I**) Schematic of Library 2 bivalent effectors cloned into a lentiviral vector for HTS. Golden gate assembly overhang positions are indicated in blue. Barcode mapping Cas9 guides are indicated in red.
(**J**) Heatmap of barcodes mapped per bivalent effector, with effectors organized by increasing size.
(**K**) Scatterplot of bivalent effector length versus number of mapped barcodes.

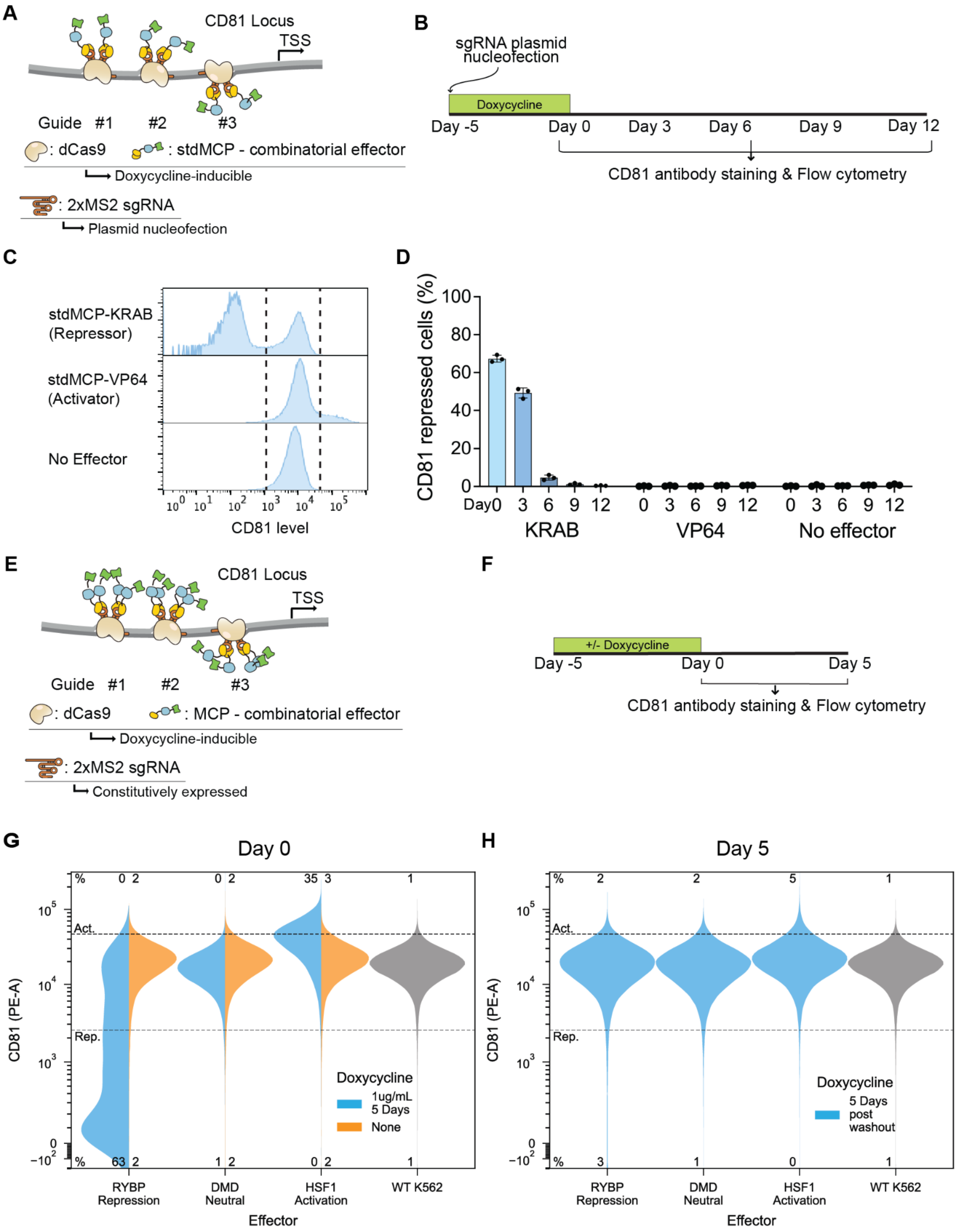
A
CD81 Locus
TSS
Guide #1 #2 #3
: dCas9
: stdMCP - combinatorial effector
Doxycycline-inducible
: 2xMS2 sgRNA
Plasmid nucleofection
B
sgRNA plasmid nucleofection
Doxycycline
Day -5
Day 0
Day 3
Day 6
Day 9
Day 12
CD81 antibody staining & Flow cytometry
C
stdMCP-KRAB (Repressor)
stdMCP-VP64 (Activator)
No Effector
CD81 level
D
CD81 repressed cells (%)
100
80
60
40
20
0
Day0 3 6 9 12
0 3 6 9 12
0 3 6 9 12
KRAB
VP64
No effector
E
CD81 Locus
TSS
Guide #1 #2 #3
: dCas9
: MCP - combinatorial effector
Doxycycline-inducible
: 2xMS2 sgRNA
Constitutively expressed
F
+/- Doxycycline
Day -5
Day 0
Day 5
CD81 antibody staining & Flow cytometry
G
Day 0
CD81 (PE-A)
Act.
Rep.
%
Doxycycline
1ug/mL 5 Days
None
RYBP Repression
DMD Neutral
HSF1 Activation
WT K562
Effector
H
Day 5
CD81 (PE-A)
Act.
Rep.
%
Doxycycline
5 Days post washout
RYBP Repression
DMD Neutral
HSF1 Activation
WT K562
Effector

Supplementary Figure 4. Doxycycline-inducible dCas9/MS2 system enables temporal control of transcriptional perturbation

(**A**) Illustration of dCas9/MS2 system for Library 1 HTS. Tet-On dCas9 K562 (without CD81 guide array) cell line was used for the experiment. Combinatorial effectors are fused to stdMCP and delivered by lentiviral infection. Both dCas9 and combinatorial effectors are inducible by doxycycline. 3X CD81 guide array is introduced by plasmid nucleofection.

(**B**) Timeline for testing dCas9/MS2 system used for Library 1 with established epigenetic repressor KRAB from ZNF10 protein, activator VP64, and no effector control.

(**C**) Representative histograms of CD81 expression at 5 days post-nucleofection of CD81 targeting 3X guide array plasmids.

(**D**) Timecourse of CD81 repressed cells after nucleofection of CD81 targeting 3X guide array plasmids. Error bars represent the mean ± standard deviation of 3 replicates.

(**E**) Illustration of dCas9/MS2 system for Library 2 HTS. Tet-On dCas9 K562 (with CD81 guide array) cell line was used for the experiment. Combinatorial effectors were fused to MCP and introduced by lentivirus. CD81 targeting sgRNAs were constitutively expressed. Both dCas9 and combinatorial effectors are inducible by doxycycline.

(**F**) Timeline for testing dCas9/MS2 system used for Library 2 with established epigenetic repressor RYBP, activator HSF1, and neutral control 400 aa fragment of DMD.

(**G**) Violin plots of CD81 expression at experimental Day 0 with or without the addition of doxycycline.

(**H**) Violin plots of CD81 expression 5 days after washout of doxycycline after initial 5 days of doxycycline treatment.

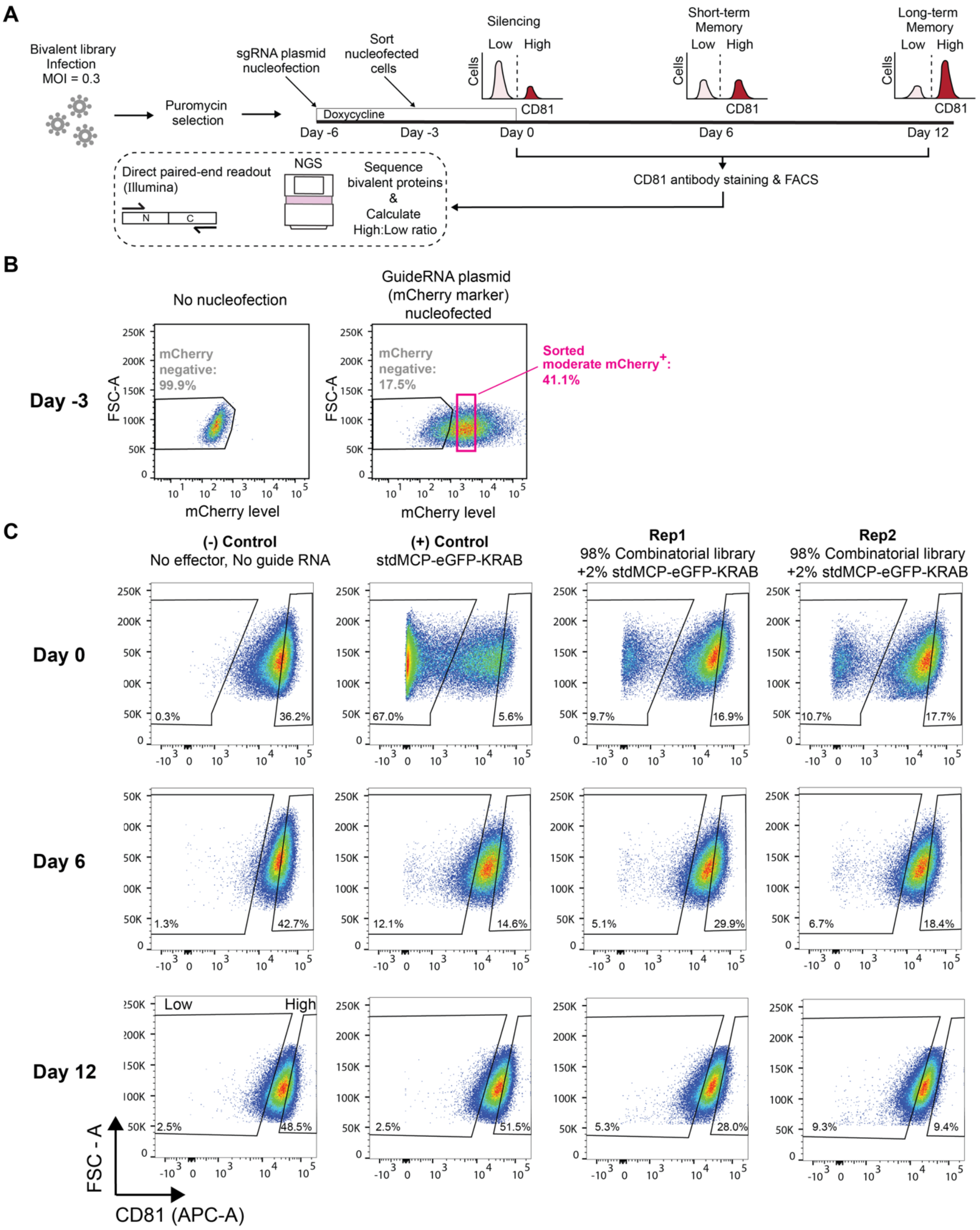

D

	Sorting criteria	Rep 1	Rep 2
Day 0	CD81 level High ~10 %	~ 0.75M	~ 1M
	CD81 level Low ~10 %	~ 0.75M	~ 1M
Day 6	CD81 level High ~8 %	~ 2.6M	~ 2.43M
	CD81 level High ~9 %	~ 2.84M	~ 2.63M
Day 12	CD81 level High ~10 %	~ 3.33M	~ 3.1M
	CD81 level Low ~10 %	~ 3.39M	~ 3.4M

Supplementary Figure 5. Detailed procedures for Library 1 HTS

(**A**) Timeline and NGS strategy for Library 1 HTS. CD81 targeting guide array plasmid was nucleofected, and nucleofected cells were sorted based on the mCherry marker present in the plasmid (**Supplementary Data 9**, Plasmids, pMH222).

(**B**) Representative FACS plots of mCherry level at 3 days post-nucleofection of 3X guide array plasmids which express mCherry as a marker. Cells that were successfully nucleofected with 3X guide array plasmids were sorted based on mCherry level. To reduce heterogeneity in the sgRNA level, moderate mCherry-expressing cells were sorted.

(**C**) FACS plots of multiple conditions from Library 1 HTS. Approximate sorting gates are drawn. The actual gating was continuously amended during sorting to match the aimed High/Low percentage of the sorted cells. This procedure was necessary as the staining (CD81, APC) histogram shifted as the sorting procedure prolonged.

(**D**) Number of cells sorted per screening condition.

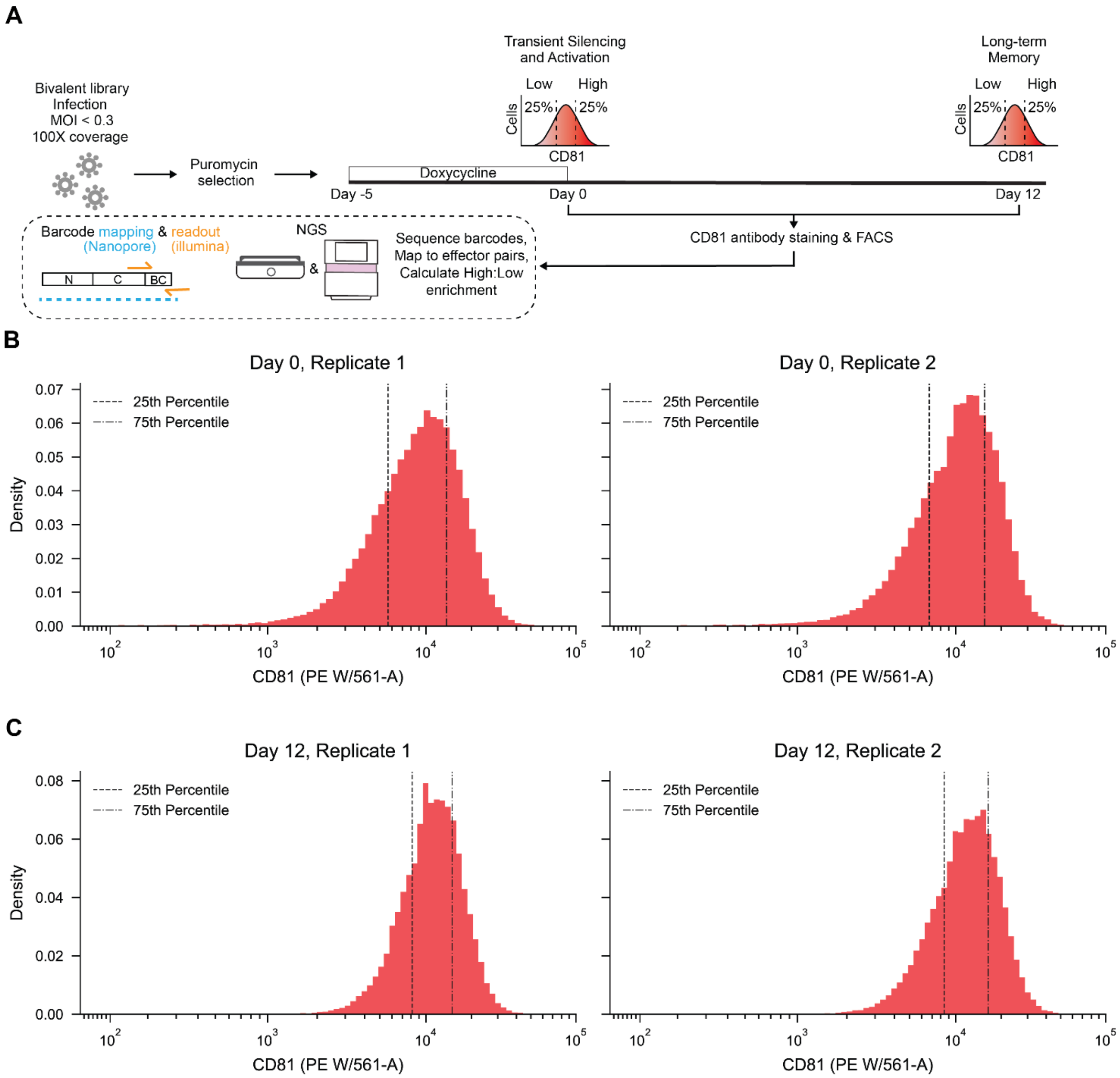

D

Day	Replicate	Low	High
0	1	12250852	10189885
0	2	8987437	7577374
12	1	8857130	7440798
12	2	11226879	9447047

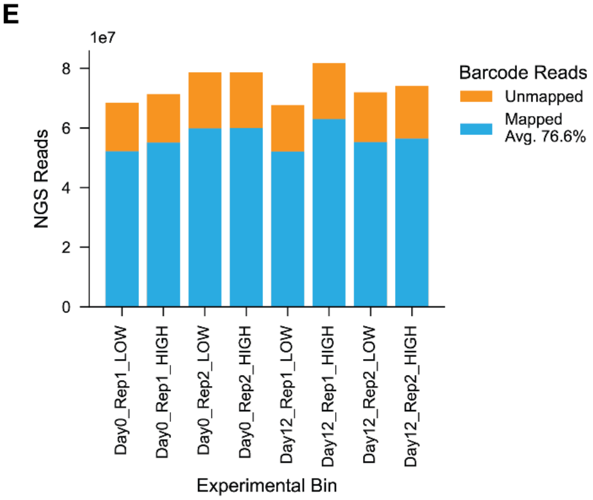

Supplementary Figure 6. Detailed procedures for Library 2 HTS and barcode mapping

(**A**) Timeline and NGS strategy for Library 2 HTS. CD81 targeting sgRNAs were constitutively expressed from Tet-On dCas9 K562 cell lines (with CD81 guide array).
(**B**) FACS plots of Library 2 HTS on Day 0 illustrating 25th and 75th percentile gates, which were continuously adjusted throughout the sort.
(**C**) FACS plots of Library 2 HTS on Day 12 illustrating 25th and 75th percentile gates, which were continuously adjusted throughout the sort.
(**D**) Number of cells sorted per screening condition.
(**E**) Number of NGS reads per screening condition with the fraction of reads mapped to bivalent effectors indicated in blue.

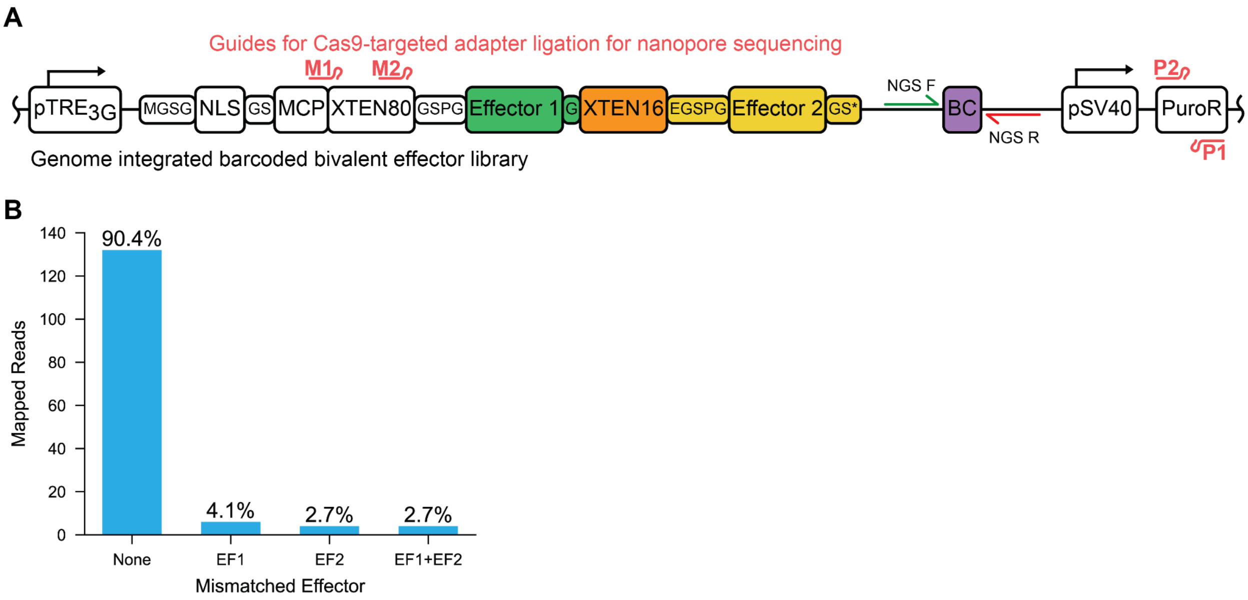

Supplementary Figure 7. Investigation of lentiviral barcode swapping by targeted nanopore sequencing

(**A**) Schematic of genome integrated bivalent effectors from Library 2. Cas9 guide positions for targeted nanopore sequencing are shown in red.

(**B**) Number and percentage of genome-integrated nanopore reads with barcodes matching or mismatching effector pairs mapped by nanopore plasmid sequencing.

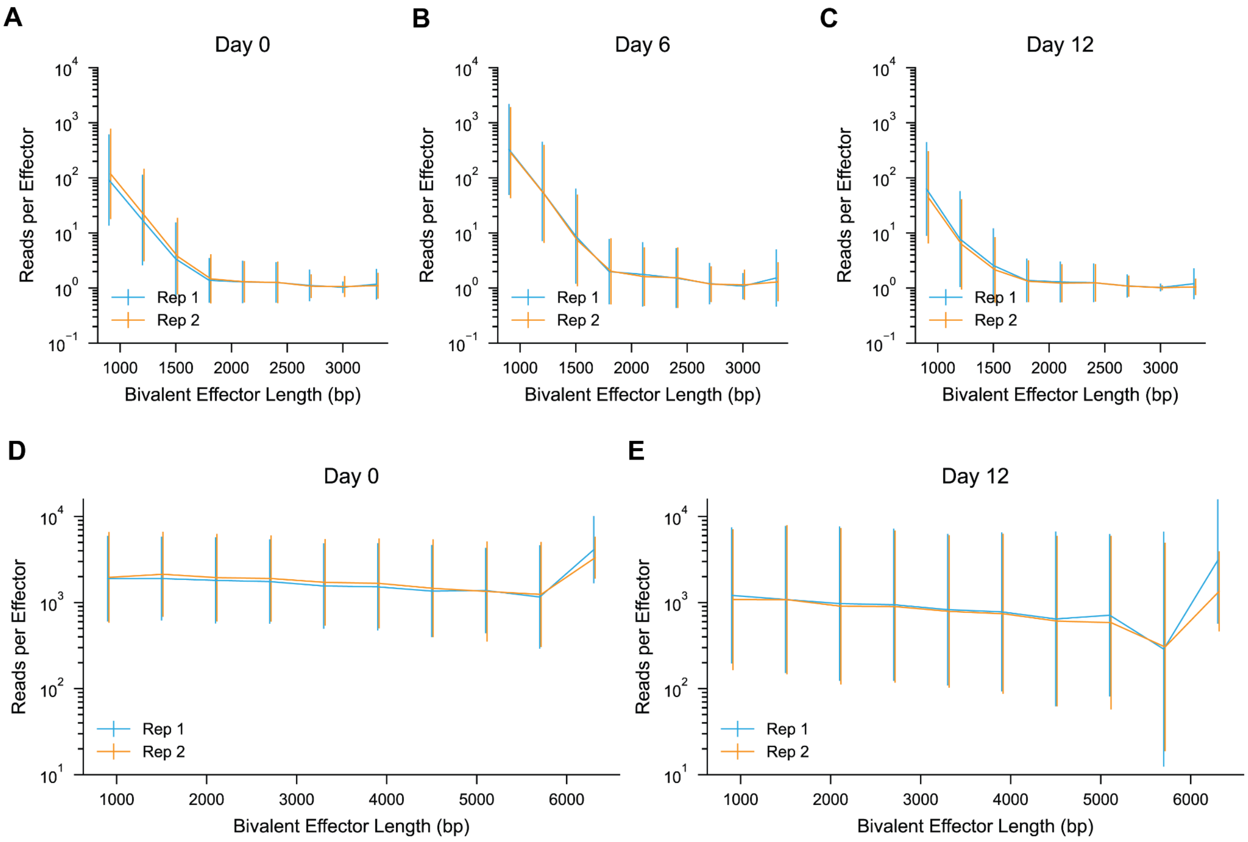

Supplementary Figure 8. Number of acquired NGS reads versus bivalent effector length

(**A**) Number of NGS reads versus length of bivalent effector from Library 1 HTS Day 0 data.
(**B**) Number of NGS reads versus length of bivalent effector from Library 1 HTS Day 6 data.
(**C**) Number of NGS reads versus length of bivalent effector from Library 1 HTS Day 12 data.
(**D**) Number of NGS barcode reads versus length of the corresponding bivalent effector from Library 2 HTS Day 0 data.
(**E**) Number of NGS barcode reads versus length of the corresponding bivalent effector from Library 2 HTS Day 12 data.

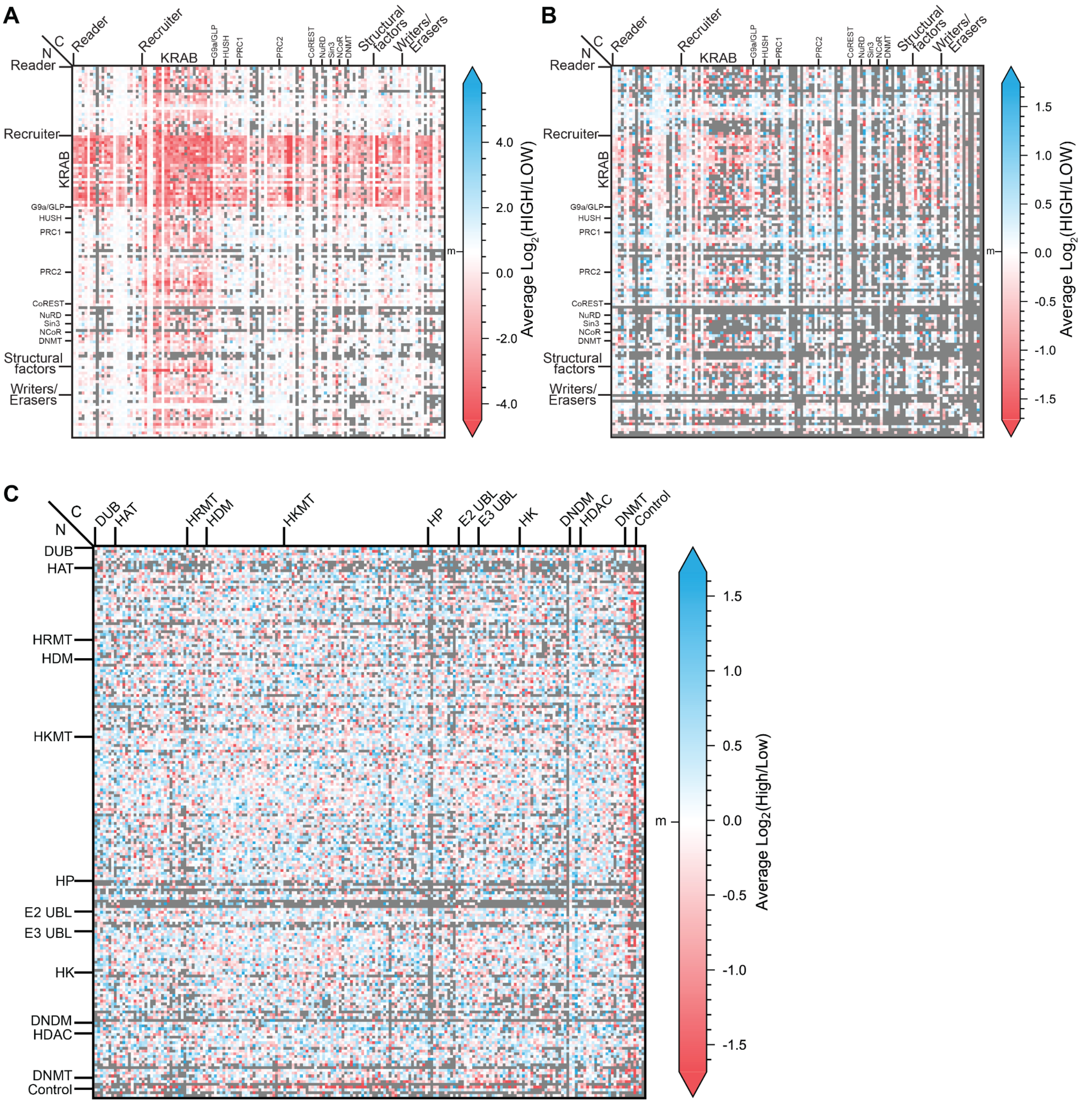

Supplementary Figure 9. Heatmaps of enrichment scores from later HTS timepoints

(**A**) Heatmap of enrichment scores generated from Library 1 at Day 6. Average Log_2(High/Low) enrichment scores across both replicates are plotted. Color scale is centered at the mode of the enrichment score distribution, indicated by the “m” tick. The maximum or minimum color saturation is determined by the 1st or 99th percentile, depending on which is farther from the mode, and the scale is symmetrically extrapolated to the same distance on the opposite side. For this repressor-focused library, lower $\log_2$ scores indicate stronger repression. Dropouts are indicated in gray.

(**B**) Heatmap of enrichment scores generated from Library 1 at Day 12. Average Log_2(High/Low) enrichment scores across both replicates are plotted. Color scale is centered at the mode of the

enrichment score distribution, indicated by the “m” tick. The maximum or minimum color saturation is determined by the 1st or 99th percentile, depending on which is farther from the mode, and the scale is symmetrically extrapolated to the same distance on the opposite side. For this repressor-focused library, lower log_2 scores indicate stronger repression. Dropouts are indicated in gray.

(**C**) Heatmap of enrichment scores generated from Library 2 at Day 12. Average Log_2(High/Low) enrichment scores across both replicates are plotted. Color scale is centered at the mode of the enrichment score distribution, indicated by the “m” tick. The maximum or minimum color saturation is determined by the 1st or 99th percentile, depending on which is farther from the mode, and the scale is symmetrically extrapolated to the same distance on the opposite side. For this library composed of putative repressors and activators, lower scores indicate more repression and higher scores indicate more activation. Dropouts are indicated in gray. Histone deubiquitinase (DUB), histone acetyltransferase (HAT), histone arginine methyltransferase (HRMT), histone demethylase (HDM), histone lysine methyltransferase (HKMT), histone phosphatase (HP), E2 ubiquitin ligases (E2 UBL), E3 ubiquitin ligases (E3 UBL), histone kinase (HK), DNA demethylation machinery (DNDM), histone deacetylase (HDAC), and DNA methyltransferase (DNMT).

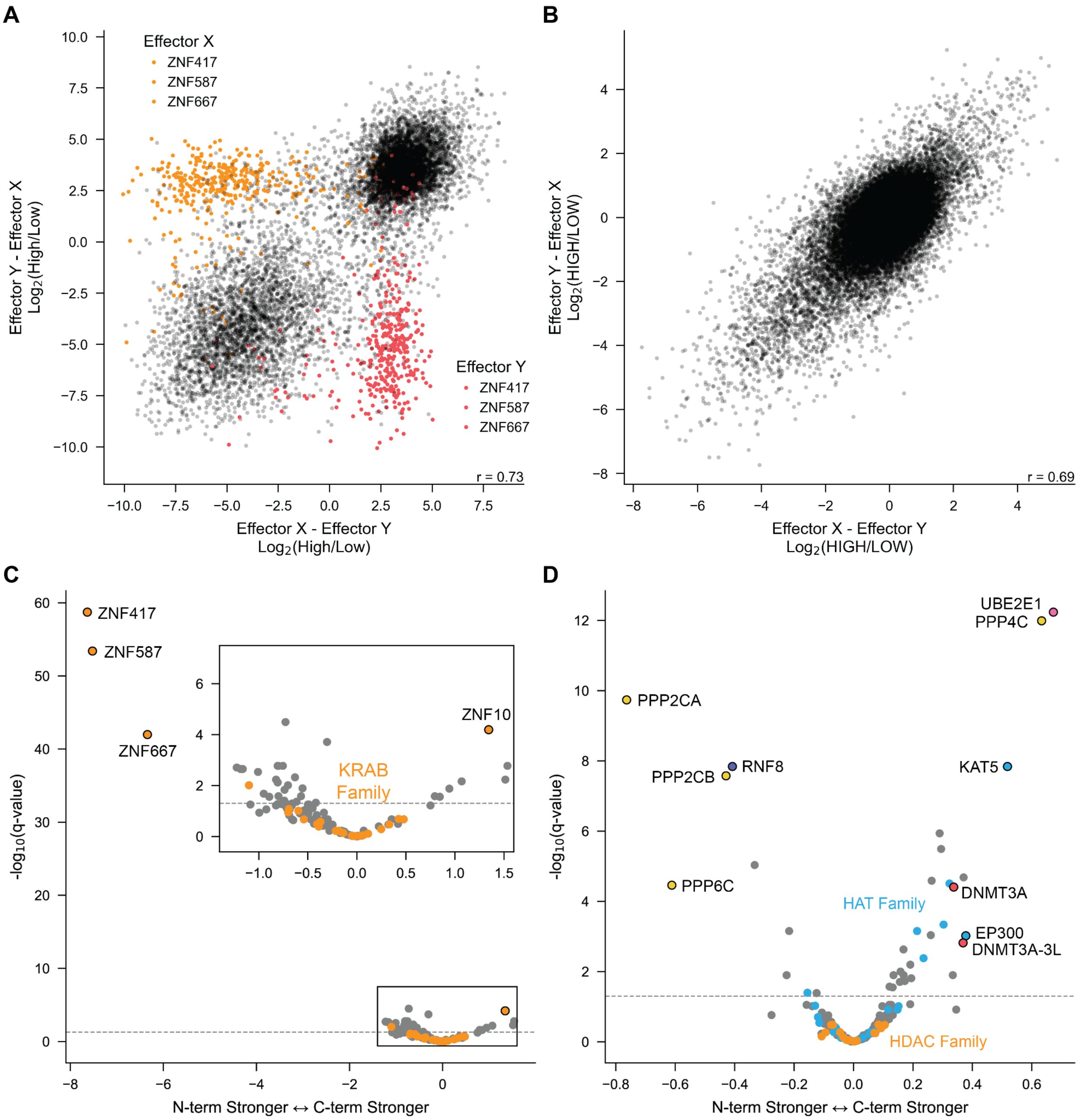

Supplementary Figure 10. N- versus C-terminal position effects

(**A**) Scatter plot of enrichment scores from X-Y versus Y-X bivalent effector pairs from Library 1 HTS on Day 0. Effectors with strong positional preference for the N-terminus are indicated in the X position in orange and the Y position in red.

(**B**) Scatter plot of enrichment scores from X-Y versus Y-X bivalent effector pairs from Library 2 HTS on Day 0.

(**C**) Volcano plot illustrating positional preference of individual effectors from Library 1. X-axis score calculated as the difference in mean $\log_2$(High/Low) between matched effector pairs with given effector at N- versus C-terminus (C-terminus minus N-terminus), multiplied by -1 for

effectors with repressive marginal scores and +1 for effectors with activating marginal scores. Q-values were calculated as FDR-corrected p-values from a two-sample t-test. Only effectors with ≥30 matched pairs are shown.

(**D**) Volcano plot illustrating positional preference of individual effectors from Library 2. Calculated as (**C**).

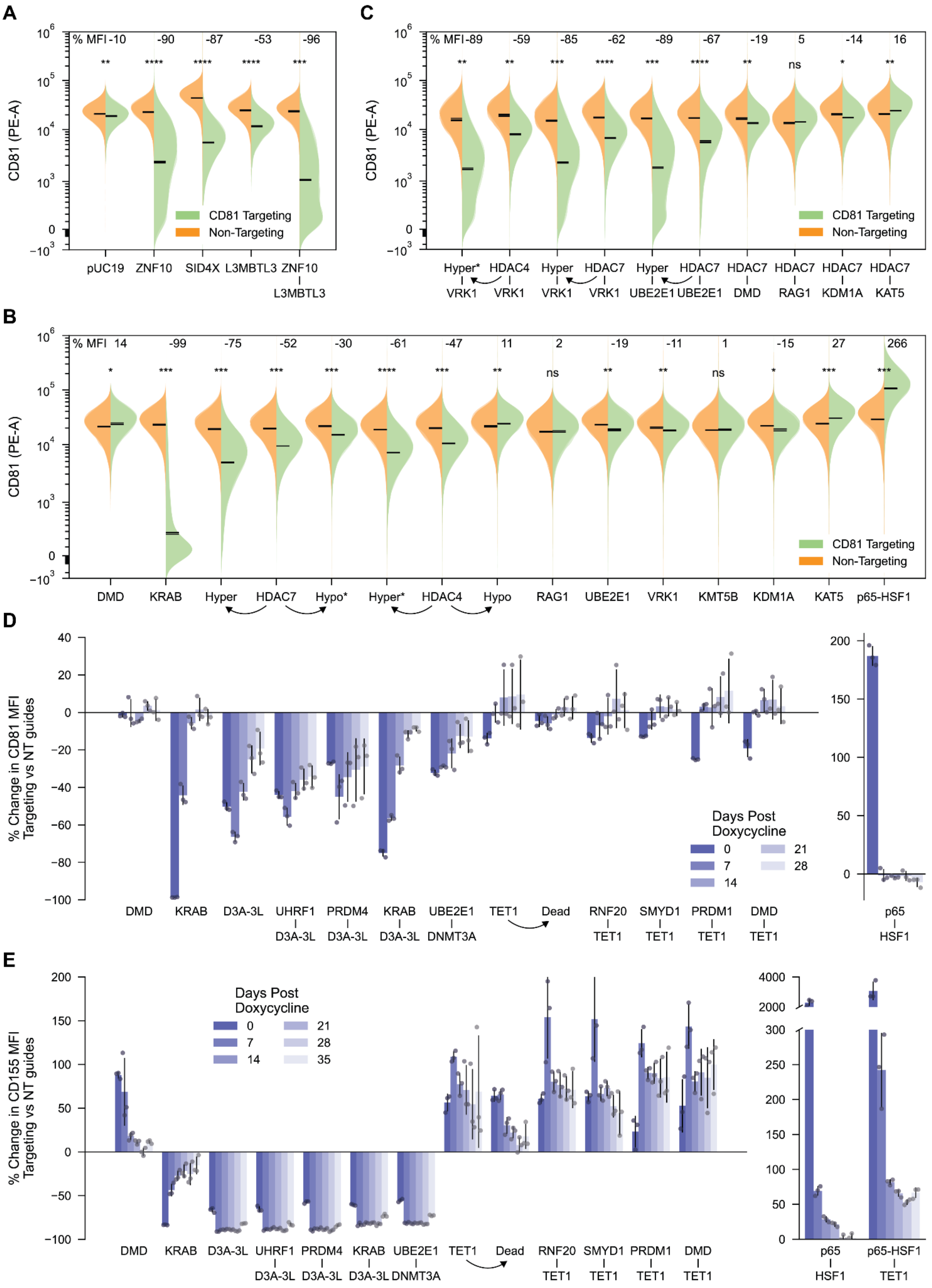
A
CD81 (PE-A)
% MFI -10 -90 -87 -53 -96
pUC19 ZNF10 SID4X L3MBTL3 ZNF10 L3MBTL3
CD81 Targeting
Non-Targeting
C
% MFI -89 -59 -85 -62 -89 -67 -19 5 -14 16
Hyper* VRK1 HDAC4 VRK1 Hyper VRK1 HDAC7 VRK1 Hyper UBE2E1 HDAC7 UBE2E1 HDAC7 DMD HDAC7 RAG1 HDAC7 KDM1A HDAC7 KAT5
B
% MFI 14 -99 -75 -52 -30 -61 -47 11 2 -19 -11 1 -15 27 266
DMD KRAB Hyper HDAC7 Hypo* Hyper* HDAC4 Hypo RAG1 UBE2E1 VRK1 KMT5B KDM1A KAT5 p65-HSF1
D
% Change in CD81 MFI Targeting vs NT guides
Days Post Doxycycline
0 7 14 21 28
DMD KRAB D3A-3L UHRF1 D3A-3L PRDM4 D3A-3L KRAB D3A-3L UBE2E1 DNMT3A TET1 Dead RNF20 TET1 SMYD1 TET1 PRDM1 TET1 DMD TET1 p65 HSF1
E
% Change in CD155 MFI Targeting vs NT guides
Days Post Doxycycline
0 7 14 21 28 35
DMD KRAB D3A-3L UHRF1 D3A-3L PRDM4 D3A-3L KRAB D3A-3L UBE2E1 DNMT3A TET1 Dead RNF20 TET1 SMYD1 TET1 PRDM1 TET1 DMD TET1 p65 HSF1 p65-HSF1 TET1

Supplementary Figure 11. Targeting versus non-targeting guides for key effectors

(**A**) Violin plots of CD81 expression at 3 days after nucleofection and induction of effectors from Library 1. Effectors nucleofected into the PB Tet-On dCas9 cell line with the 3X CD81 guide array colored green. Effectors nucleofected into the PB Tet-On dCas9 cell line with the 3X NT guide array colored orange. 3 independent replicates are illustrated as translucent overlays. The geometric means of each replicate are shown as solid black lines. The average percent changes in CD81 MFI for CD81 targeting versus non-targeting guides for each effector are indicated. Geometric means from targeting versus non-targeting conditions for each effector were tested for statistically significant differences using Welch's t-test. Significance levels are indicated as: *$p \leq 0.05$, **$p \leq 0.01$, ***$p \leq 0.001$, ****$p \leq 0.0001$, ns = not significant.

(**B**) Violin plots of CD81 expression at 5 days after nucleofection and induction of monovalent effectors from Library 2. Described as (**A**). *proposed mutant inferred from homology. Arrows point from WT effector to mutant form of the same effector.

(**C**) Violin plots of CD81 expression at 5 days after nucleofection and induction of HDAC combinations from Library 2. Described as (**A**). *proposed mutant inferred from homology. Arrows point from WT effector to mutant form of the same effector.

(**D**) Timecourse of percent change in CD81 MFI for CD81 targeting versus non-targeting guides for long-term effectors from Library 2 and key controls including DMD, KRAB, and p65-HSF1. Error bars represent the mean ± standard deviation between 3 independent replicates. Arrows point from WT effector to mutant form of the same effector.

(**E**) Timecourse of percent change in CD155 MFI for CD155 targeting versus non-targeting guides for long-term effectors from Library 2 and key controls including DMD, KRAB, and p65-HSF1. Error bars represent the mean ± standard deviation between 3 independent replicates. Arrows point from WT effector to mutant form of the same effector.

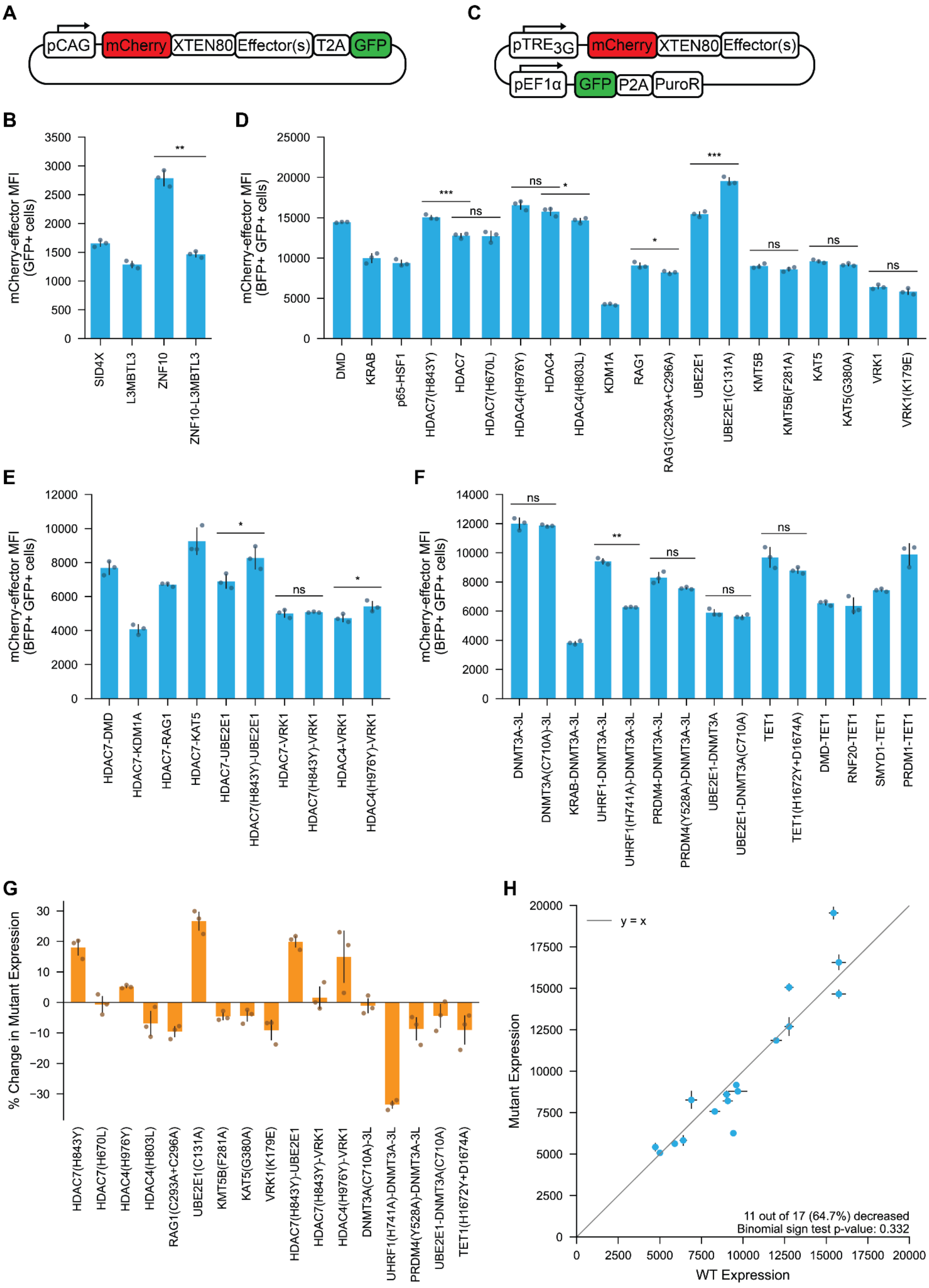
A
pCAG
mCherry
XTEN80
Effector(s)
T2A
GFP
C
pTRE3G
mCherry
XTEN80
Effector(s)
pEF1α
GFP
P2A
PuroR
B
mCherry-effector MFI (GFP+ cells)
SID4X
L3MBTL3
ZNF10
ZNF10-L3MBTL3
D
mCherry-effector MFI (BFP+ GFP+ cells)
DMD
KRAB
p65-HSF1
HDAC7(H843Y)
HDAC7
HDAC7(H670L)
HDAC4(H976Y)
HDAC4
HDAC4(H803L)
KDM1A
RAG1
RAG1(C293A+C296A)
UBE2E1
UBE2E1(C131A)
KMT5B
KMT5B(F281A)
KAT5
KAT5(G380A)
VRK1
VRK1(K179E)
E
mCherry-effector MFI (BFP+ GFP+ cells)
HDAC7-DMD
HDAC7-KDM1A
HDAC7-RAG1
HDAC7-KAT5
HDAC7-UBE2E1
HDAC7(H843Y)-UBE2E1
HDAC7-VRK1
HDAC7(H843Y)-VRK1
HDAC4-VRK1
HDAC4(H976Y)-VRK1
F
mCherry-effector MFI (BFP+ GFP+ cells)
DNMT3A-3L
DNMT3A(C710A)-3L
KRAB-DNMT3A-3L
UHRF1-DNMT3A-3L
UHRF1(H741A)-DNMT3A-3L
PRDM4-DNMT3A-3L
PRDM4(Y528A)-DNMT3A-3L
UBE2E1-DNMT3A
UBE2E1-DNMT3A(C710A)
TET1
TET1(H1672Y+D1674A)
DMD-TET1
RNF20-TET1
SMYD1-TET1
PRDM1-TET1
G
% Change in Mutant Expression
HDAC7(H843Y)
HDAC7(H670L)
HDAC4(H976Y)
HDAC4(H803L)
RAG1(C293A+C296A)
UBE2E1(C131A)
KMT5B(F281A)
KAT5(G380A)
VRK1(K179E)
HDAC7(H843Y)-UBE2E1
HDAC7(H843Y)-VRK1
HDAC4(H976Y)-VRK1
DNMT3A(C710A)-3L
UHRF1(H741A)-DNMT3A-3L
PRDM4(Y528A)-DNMT3A-3L
UBE2E1-DNMT3A(C710A)
TET1(H1672Y+D1674A)
H
y = x
Mutant Expression
WT Expression
11 out of 17 (64.7%) decreased
Binomial sign test p-value: 0.332

Supplementary Figure 12. Assessment of expression levels of effector and catalytic mutants

(**A**) Schematic of plasmids used to assess expression of effectors from Library 1. mCherry-effector(s) fusions and GFP are driven by the CAG promoter.

(**B**) mCherry-effector MFIs of Library 1 effectors in GFP+ nucleofected cells. Measured 3 days after nucleofection into PB Tet-On dCas9 K562 with 3X NT guide array. Error bars represent mean ± standard deviation between 3 independent replicates. Black horizontal bar represents statistical testing between ZNF10 and ZNF10-L3MBTL3 using Welch's t-test. Significance levels are indicated as: *$p \leq 0.05$, **$p \leq 0.01$, ***$p \leq 0.001$, ****$p \leq 0.0001$, ns = not significant.

(**C**) Schematic of plasmids used to assess expression of effectors from Library 2. mCherry-XTEN80-Effector(s) are expressed by doxycycline-inducible TRE3G promoter. GFP and puromycin resistance gene are expressed by EF1α promoter.

mCherry-effector MFIs of individual effectors (**D**), HDAC combinations (**E**), and long-term effectors (**F**) and mutants from Library 2 in GFP+ nucleofected cells and BFP+ cells expressing reverse tetracycline-controlled transactivator 3 (rtTA3). Measured 5 days after nucleofection into PB Tet-On dCas9 K562 with 3X NT guide array and doxycycline induction. Error bars represent mean ± standard deviation between 3 independent replicates. Black horizontal bars represent statistical testing between WT effectors and catalytic mutants using Welch's t-test. Significance levels are indicated as: *$p \leq 0.05$, **$p \leq 0.01$, ***$p \leq 0.001$, ****$p \leq 0.0001$, ns = not significant.

(**G**) Percent change in mutant expression versus WT effectors calculated from data above on a per replicate basis. Error bars represent mean ± standard deviation between 3 independent replicates.

(**H**) Scatter plot representing WT effector expression versus mutant effector expression for all tested effector/mutant pairs. Vertical and horizontal error bars represent mean ± standard deviations between 3 independent replicates. Black diagonal line shows $y = x$, indicating an equal expression level. 11 out of 17 effector/mutant pairs showed decreased expression yielding an insignificant binomial sign test p-value.

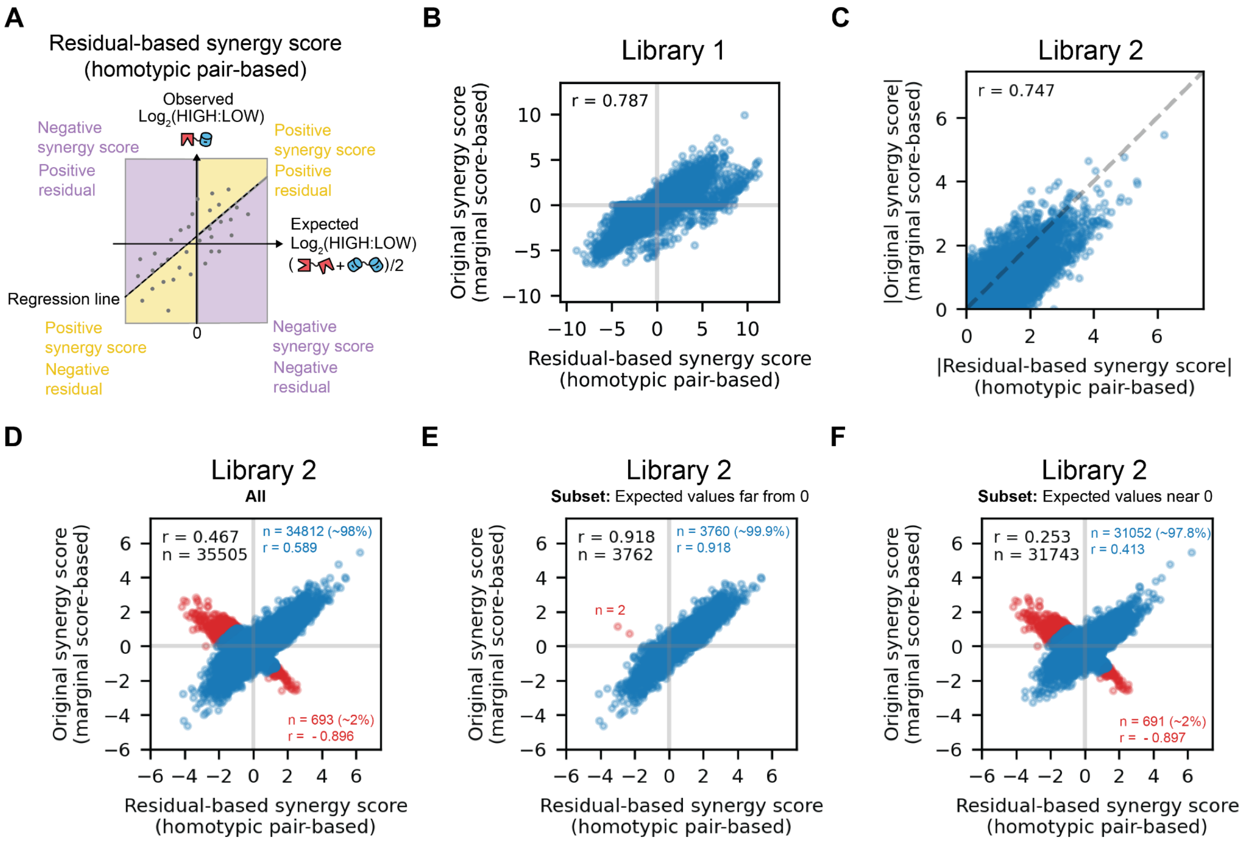

Supplementary Figure 13. Comparison of original synergy scores with residual-based synergy scores using homotypic pairs as an alternative metric

(**A**) Schematic illustration of residual-based synergy score calculation using homotypic pairs. All Log_2(HIGH:LOW) values are first mode-corrected by subtracting the mode of the enrichment score distribution to center the data. For a heterodimer A-B, the expected value is calculated as the average of the two corresponding homodimer measurements: (A-A + B-B)/2. The observed value is the mode-corrected measured enrichment score for the A-B heterodimer. A regression line is fit to the observed versus expected values. For Library 1, which is mainly composed of repressors, the residual (deviation from regression line) sign is inverted to be consistent with the biological meaning of synergy (more repression than expected repression). For Library 2, which is composed of both repressors and activators, the synergy score is derived from the residual with sign adjustment: for pairs with negative expected values (expected < 0), the residual sign is inverted; for pairs with positive expected values (expected > 0), the residual equals the synergy score. Quadrants defined by the y-axis (expected value = 0) and the regression line show the interpretation.

(**B**) Correlation between original synergy scores and residual-based synergy scores for Library 1 Day 0. Pearson correlation r = 0.787. The positive correlation indicates general agreement between the two methods.

(**C**) Correlation between absolute values of original synergy scores and residual-based synergy scores for Library 2 Day 0. The dashed gray line represents perfect correlation (y = x). Pearson correlation r = 0.747, indicating moderate agreement between methods.

(**D**) Correlation between raw values of original synergy scores and residual-based synergy scores for Library 2 Day 0. Blue points (n = 34812, ~98%) fall within 4.5σ of the regression line obtained from (**E**), representing cases where both methods show concordant behavior (r = 0.589). Red points (n = 693, ~2%) fall outside this threshold, representing outliers with strong anti-correlation (r = -0.896) between methods. The strong anti-correlation indicates concordant results in absolute values of effect size between the two methods but with inversion of sign, due to sign flipping when the expected value is near zero.

(**E**) Subset analysis of Library 2 pairs with expected values far from zero (|sum of marginal scores| ≥ 0.3 and |average enrichment score of homotypic pairs| ≥ 1). This high-confidence subset shows strong correlation (r = 0.918, n = 3762), with 99.9% of points (n = 3760, blue) falling within the expected pattern and only 2 red outlier points.

(**F**) Subset analysis of Library 2 pairs with expected values near zero (excluded from the criteria in (**E**)). This subset shows weak correlation (r = 0.253, n = 31743), with 97.8% of points (n = 31052, blue) within the pattern but 2% (n = 691, red) showing strong anti-correlation (r = -0.897).

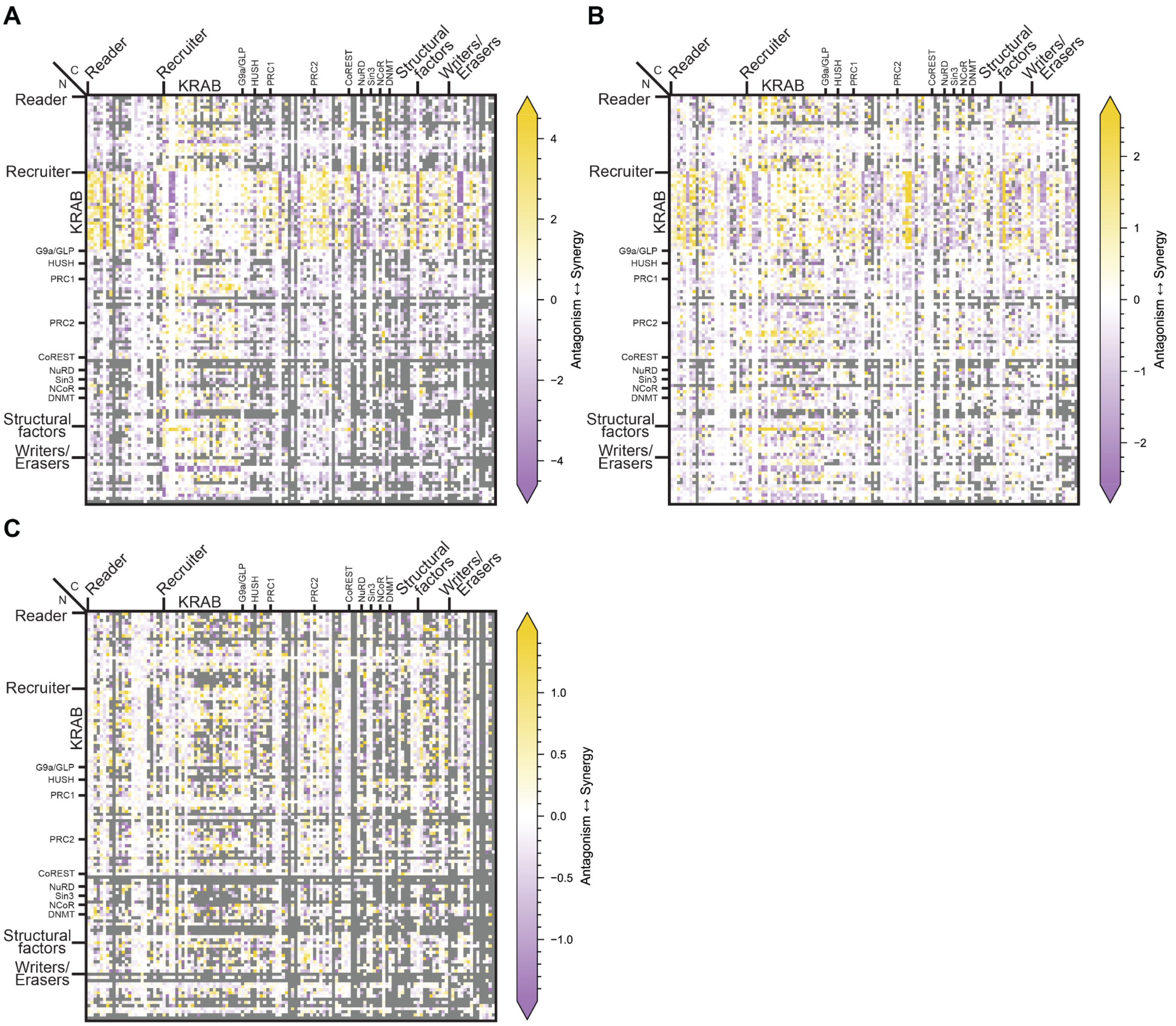

Supplementary Figure 14. Synergy score heatmaps highlight synergistic and antagonistic interactions in Library 1

Heatmaps illustrating synergy scores for bivalent combinations in Library 1 on Day 0 (**A**), Day 6 (**B**), and Day 12 (**C**). Purple indicates antagonism where measured scores were less repressive than expected, and yellow indicates synergy where scores were more repressive than expected. The magnitude of the score represents how far measured scores were outside of the expected (additive) range. Colorbar scale is linear and centered at 0, which indicates additivity. Dropouts are indicated in gray.

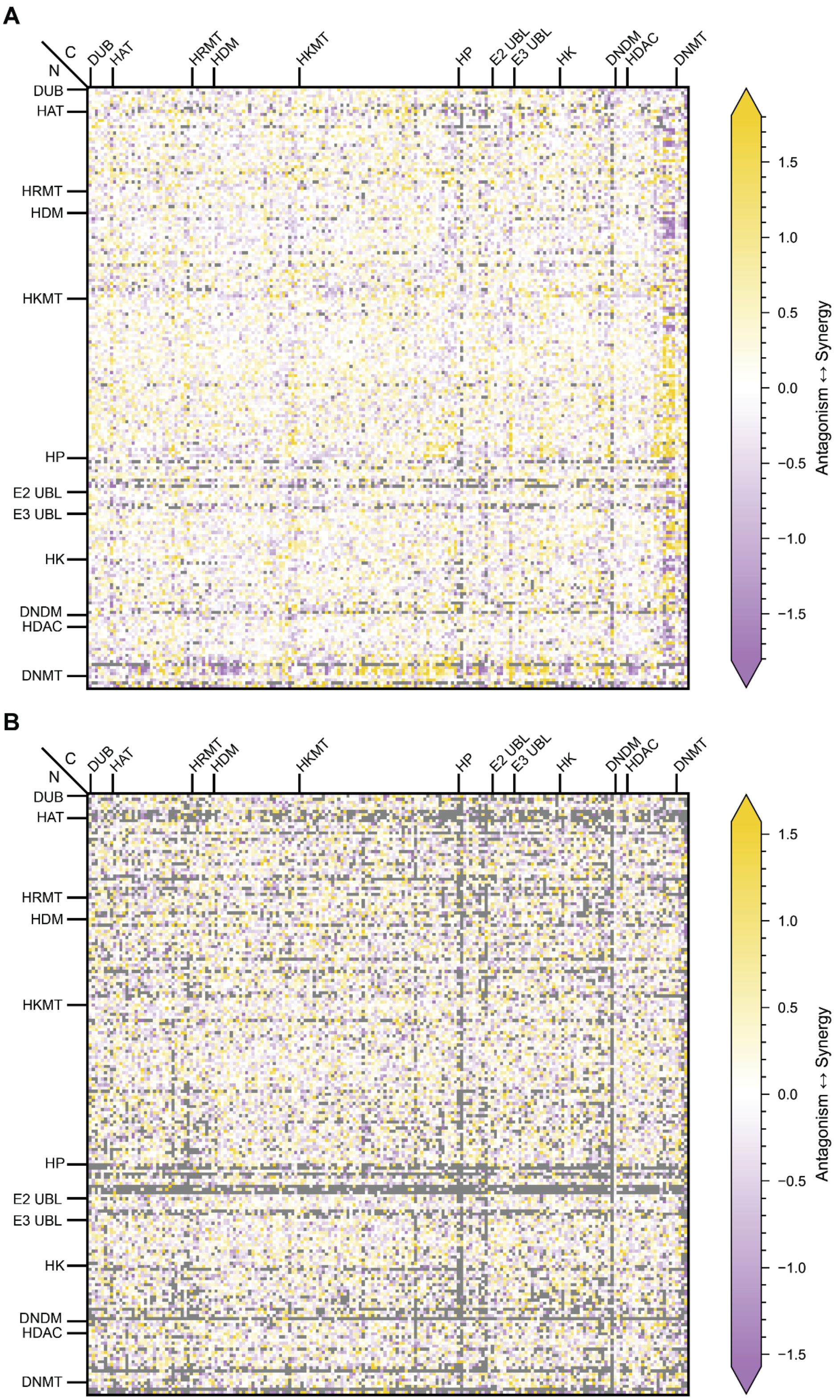

Supplementary Figure 15. Synergy score heatmaps highlight synergistic and antagonistic interactions in Library 2

Heatmaps illustrating synergy scores for bivalent combinations in Library 2 on Day 0 (**A**) and Day 12 (**B**). Purple indicates antagonism where measured scores were less repressive or

activating than expected, and yellow indicates synergy where scores were more repressive or activating than expected. Scores were computed in the direction of the stronger effector. The magnitude of the score represents how far measured scores were outside of the expected (additive) range. Colorbar scale is linear and centered at 0, which indicates additivity. Dropouts are indicated in gray. Histone deubiquitinase (DUB), histone acetyltransferase (HAT), histone arginine methyltransferase (HRMT), histone demethylase (HDM), histone lysine methyltransferase (HKMT), histone phosphatase (HP), E2 ubiquitin ligases (E2 UBL), E3 ubiquitin ligases (E3 UBL), histone kinase (HK), DNA demethylation machinery (DNDM), histone deacetylase (HDAC), and DNA methyltransferase (DNMT).

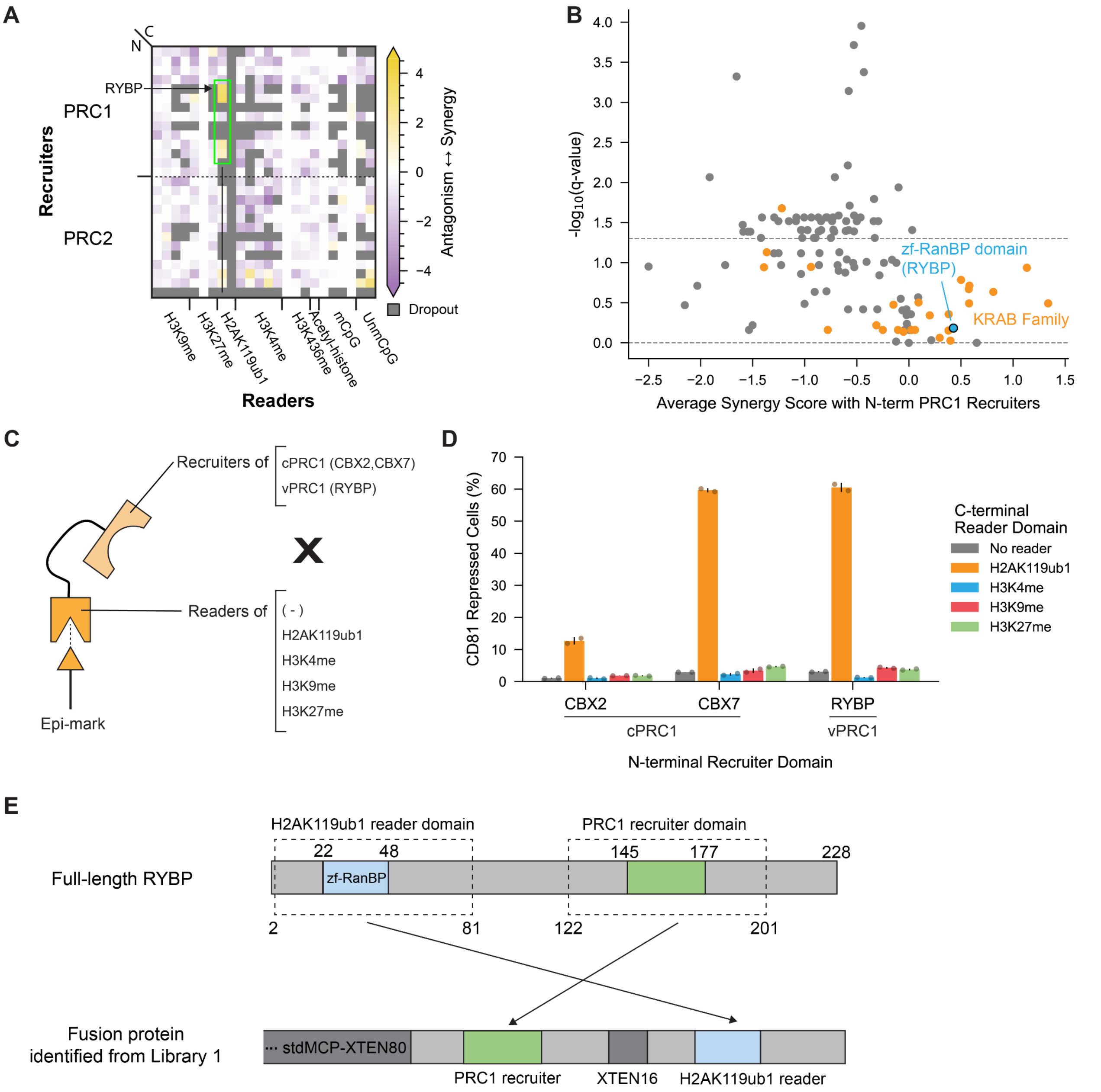

Supplementary Figure 16. Synergistic interaction between the H2AK119ub1 reader and a subset of PRC1 recruiters

(**A**) Sub-heatmap of synergy scores from Library 1 HTS Day 0 data with PRC recruiters on the N-terminus and readers of epigenetic modifications on the C-terminus. The green box indicates synergistic combinations between PRC1 recruiters and the H2AK119ub1 reader domain from the RYBP protein. The PRC1 recruiting domain from the RYBP protein is indicated by a horizontal arrow.

(**B**) Volcano plot of average synergy scores with PRC1 recruiters on the N-terminus from Library 1 HTS Day 0 data. Combinations with the KRAB family on the C-terminus are colored orange. Combination with the H2AK119ub1 mark reader, the zf-RanBP domain from RYBP protein, on

the C-terminus is colored blue. Q-values were calculated as FDR-corrected p-values from a one-sample Wilcoxon test. Horizontal dashed lines indicate q-value of 0.05 and 1.
(**C**) Illustration of PRC1 recruiter + H2AK119ub1 reader fusion proteins tested for validation.
(**D**) Percentage of CD81 repressed cells at 4 days post-nucleofection of fusion protein plasmids. CBX2 and CBX7 are components of canonical PRC1 (cPRC1) whereas RYBP is a component of variant PRC1 (vPRC1). Error bars indicate the mean ± standard deviation of 2 independent replicates.
(**E**) Illustration of full-length RYBP and potent fusion protein identified from Library1 HTS on Day 0.

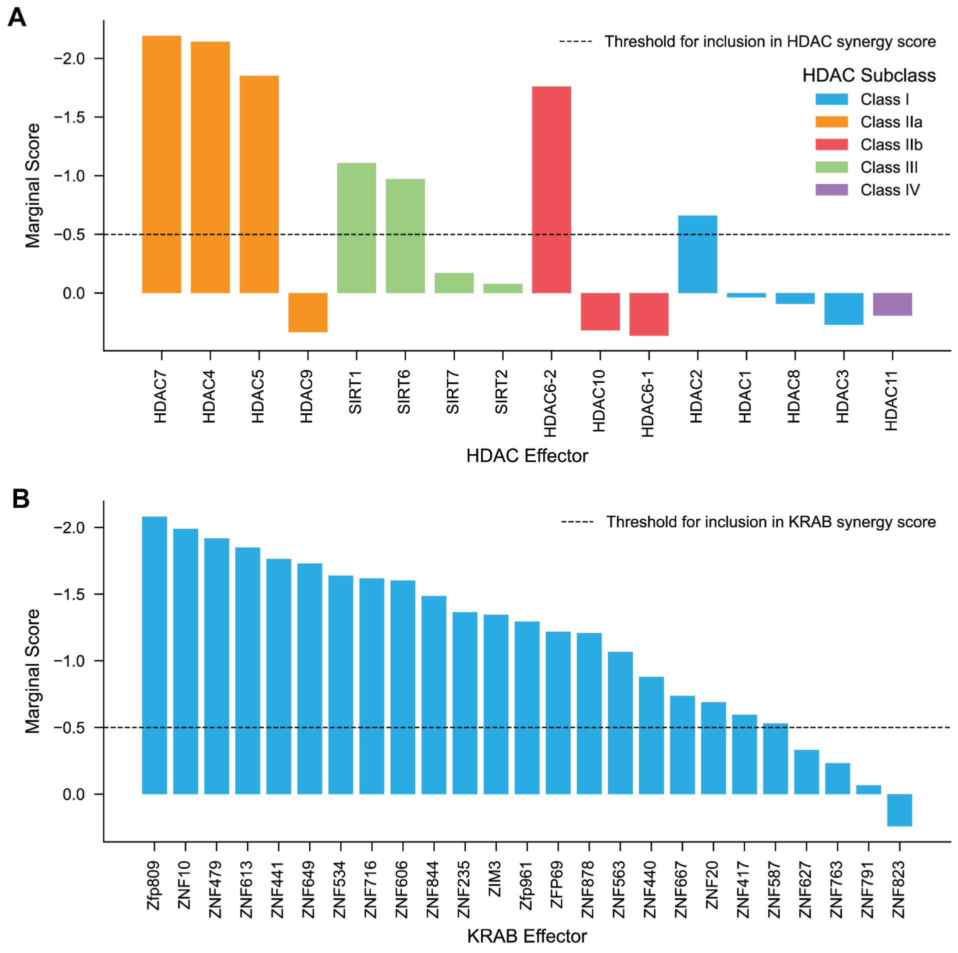

Supplementary Figure 17. HDAC and KRAB members included in the average synergy score calculation

(**A**) Marginal scores of HDAC members from Library 2 stratified by HDAC subclass. The dashed line indicates the threshold for inclusion in the average synergy score calculation. Only members with marginal scores less than -0.5 were included.

(**B**) Marginal scores of KRAB members from Library 1. The dashed line indicates the threshold for inclusion in the average synergy score calculation. Only members with marginal scores less than -0.5 were included.

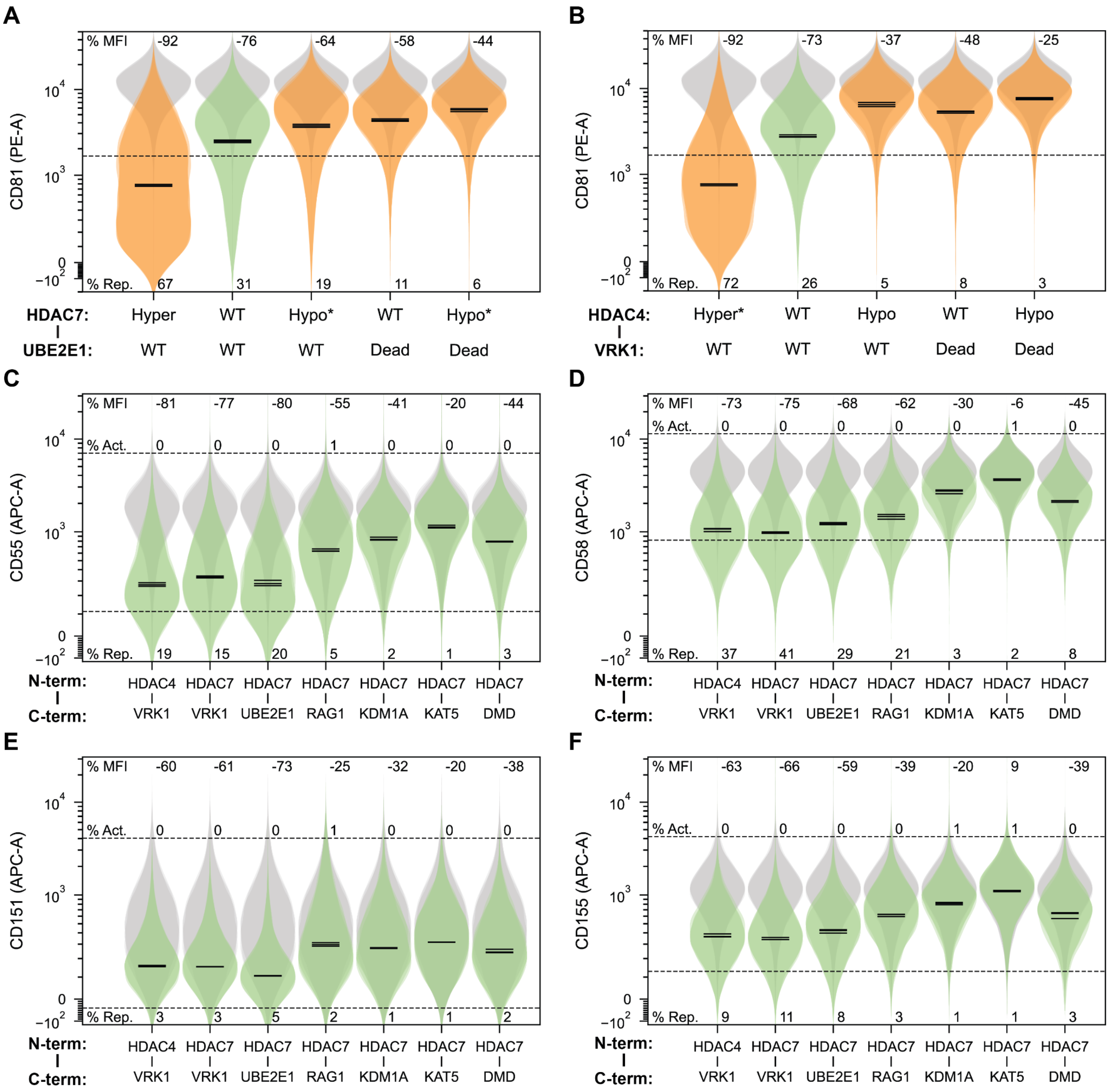

Supplementary Figure 18. HDAC combinations show catalytic dependence for CD81 repression, and also perturb multiple surface proteins

(**A**) Violin plots of CD81 expression at 5 days after nucleofection and induction of the HDAC7 + UBE2E1 combination and its mutants. WT domains are colored green. Mutant combinations are colored orange. MCP-DMD neutral condition is colored in gray. 3 independent replicates are illustrated as translucent overlays. The geometric means of each replicate are shown as solid black lines. Dashed line indicates repression gate at the 1st percentile of neutral condition. The average percentages of repressed cells in each condition are indicated. The average percent changes in CD81 MFI versus DMD for each condition are indicated. *proposed mutant inferred from homology to HDAC4.

(**B**) Violin plots of CD81 expression at 5 days after nucleofection and induction of HDAC4 + VRK1 and enzymatic mutants. WT domains are colored in green. Mutant combinations are

colored orange. MCP-DMD neutral condition colored gray. 3 independent replicates are illustrated as translucent overlays. The geometric means of each replicate are shown as solid black lines. Dashed line indicates repression gate at the 1st percentile of neutral condition. The average percentages of repressed cells in each condition are indicated. The average percent changes in CD81 MFI versus DMD for each condition are indicated. *proposed mutant inferred from HDAC7.

(**C**) Violin plots of CD55 expression at 5 days after nucleofection and induction of WT HDAC combinations, colored in green. MCP-DMD neutral condition is colored in gray. 3 independent replicates are illustrated as translucent overlays. The geometric means of each replicate are shown as solid black lines. Dashed lines indicate repression and activation gate at the 1st and 99th percentile of neutral condition. The average percentages of repressed and activated cells in each condition are indicated. The average percent changes in MFI versus the neutral DMD domain for each condition are indicated.

(**D**) Violin plots of CD58 expression at 5 days after nucleofection and induction of WT HDAC combinations. Described as (**C**).

(**E**) Violin plots of CD151 expression at 5 days after nucleofection and induction of WT HDAC combinations. Described as (**C**).

(**F**) Violin plots of CD155 expression at 5 days after nucleofection and induction of WT HDAC combinations. Described as (**C**).

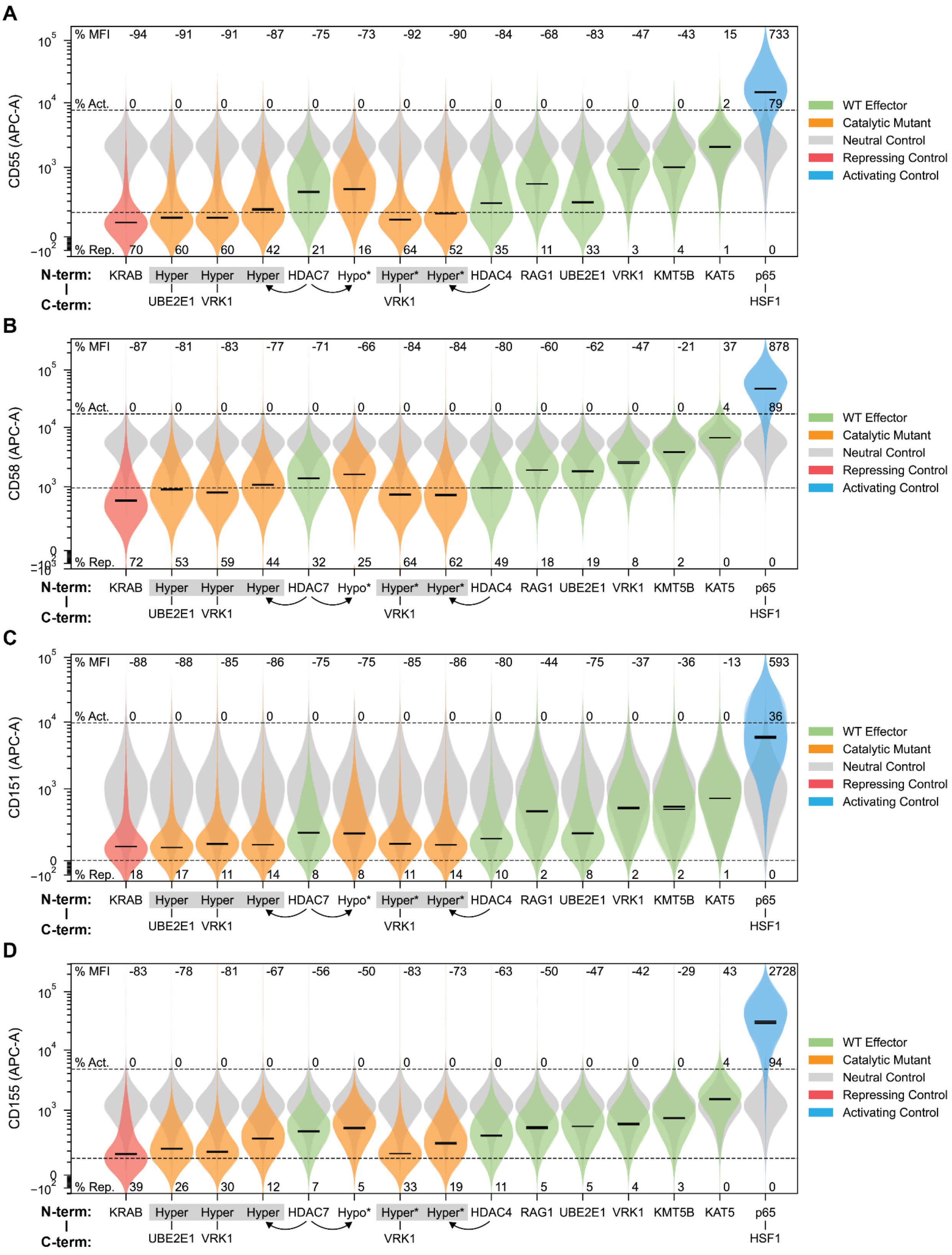

A
CD55 (APC-A)
% MFI -94 -91 -91 -87 -75 -73 -92 -90 -84 -68 -83 -47 -43 15 733
% Act. 0 0 0 0 0 0 0 0 0 0 0 0 0 2 79
% Rep. 70 60 60 42 21 16 64 52 35 11 33 3 4 1 0
B
CD58 (APC-A)
% MFI -87 -81 -83 -77 -71 -66 -84 -84 -80 -60 -62 -47 -21 37 878
% Act. 0 0 0 0 0 0 0 0 0 0 0 0 0 4 89
% Rep. 72 53 59 44 32 25 64 62 49 18 19 8 2 0 0
C
CD151 (APC-A)
% MFI -88 -88 -85 -86 -75 -75 -85 -86 -80 -44 -75 -37 -36 -13 593
% Act. 0 0 0 0 0 0 0 0 0 0 0 0 0 0 36
% Rep. 18 17 11 14 8 8 11 14 10 2 8 2 2 1 0
D
CD155 (APC-A)
% MFI -83 -78 -81 -67 -56 -50 -83 -73 -63 -50 -47 -42 -29 43 2728
% Act. 0 0 0 0 0 0 0 0 0 0 0 0 0 4 94
% Rep. 39 26 30 12 7 5 33 19 11 5 5 4 3 0 0
N-term: KRAB Hyper Hyper Hyper HDAC7 Hypo* Hyper* Hyper* HDAC4 RAG1 UBE2E1 VRK1 KMT5B KAT5 p65
C-term: UBE2E1 VRK1 VRK1 HSF1
WT Effector
Catalytic Mutant
Neutral Control
Repressing Control
Activating Control

Supplementary Figure 19. Key effectors from Library 2 transiently perturb CD55, CD58, CD151, and CD155

(**A**) Violin plots of CD55 expression at 5 days after nucleofection and induction of key effectors identified from Library 2. WT domains are colored green. Mutants or combinations containing mutants are colored orange. Repressing and activating controls are colored red and blue respectively. MCP-DMD neutral condition is colored in gray. 3 independent replicates are illustrated as translucent overlays. The geometric means of each replicate are shown as solid black lines. Dashed lines indicate repression and activation gate at the 1st and 99th percentile of neutral condition. The average percentages of repressed and activated cells in each condition are indicated. The average percent changes in MFI versus the neutral DMD domain for each condition are indicated. *proposed mutant inferred from homology. Lack of C-terminal effector indicates that the effector was tested monovalently. Arrows point from WT effector to mutant form of the same effector. Gray boxes indicate the same effector.

(**B**) Violin plots of CD58 expression at 5 days after nucleofection and induction of key effectors identified from Library 2. Described as (**A**).

(**C**) Violin plots of CD151 expression at 5 days after nucleofection and induction of key effectors identified from Library 2. Described as (**A**).

(**D**) Violin plots of CD155 expression at 5 days after nucleofection and induction of key effectors identified from Library 2. Described as (**A**).

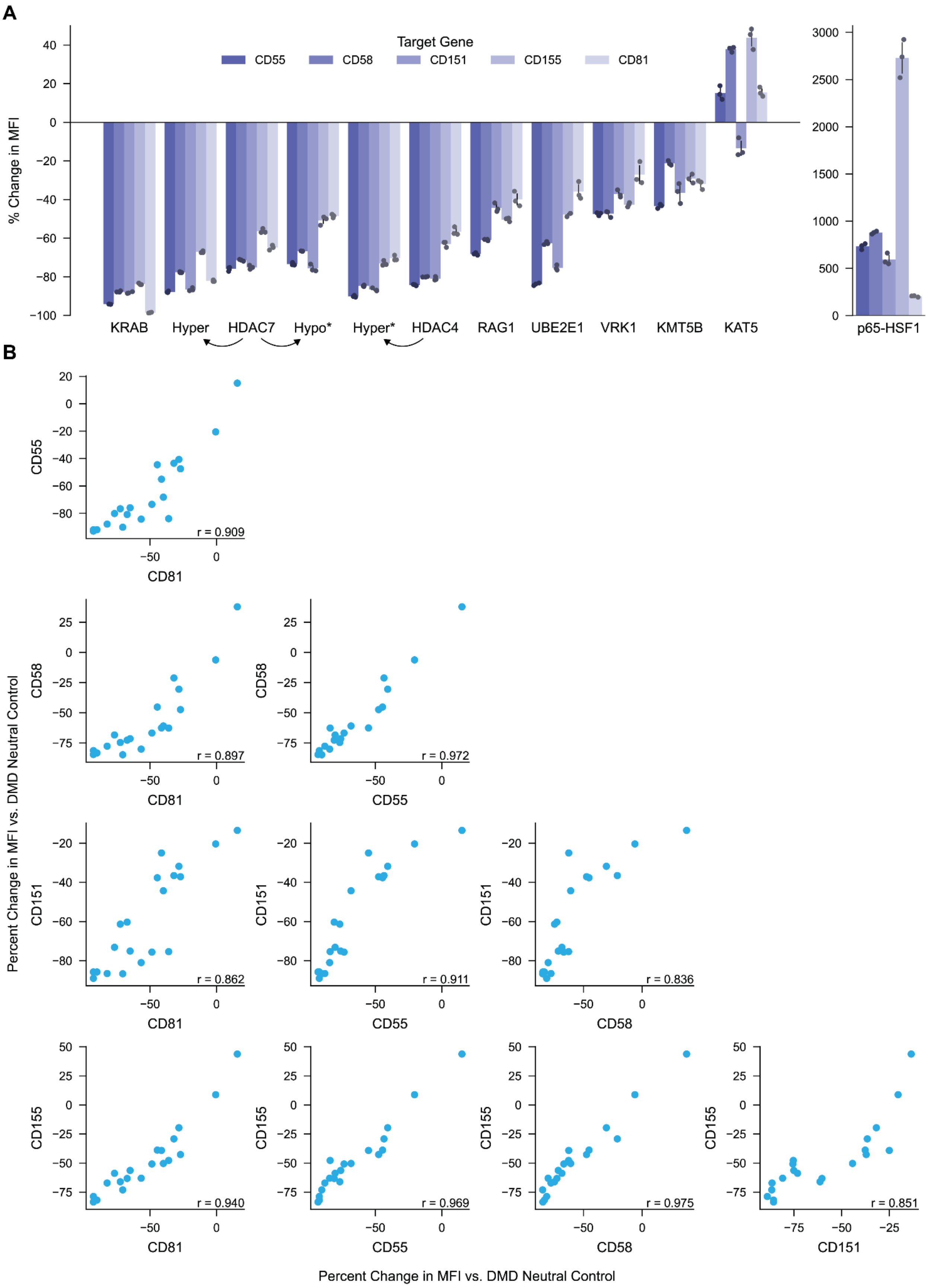

A
B
Target Gene
CD55
CD58
CD151
CD155
CD81
% Change in MFI
KRAB
Hyper
HDAC7
Hypo*
Hyper*
HDAC4
RAG1
UBE2E1
VRK1
KMT5B
KAT5
p65-HSF1
r = 0.909
r = 0.897
r = 0.972
r = 0.862
r = 0.911
r = 0.836
r = 0.940
r = 0.969
r = 0.975
r = 0.851
Percent Change in MFI vs. DMD Neutral Control
Percent Change in MFI vs. DMD Neutral Control

Supplementary Figure 20. Percent changes in MFI induced by key monovalent effectors from Library 2 with gene-by-gene scatter plots of all effectors

(**A**) Percent change in MFI relative to the neutral DMD control following dual nucleofection of plasmids encoding key monovalent effectors from Library 2 and 3X guide arrays targeting CD55, CD58, CD151, and CD155. CD81 percent MFIs are from single plasmid nucleofection into Tet-On dCas9 cell line with preinstalled 3X CD81 guide array. The dCas9/MS2 complex was induced by doxycycline for 5 days. Error bars represent the mean ± standard deviation between 3 independent replicates. *proposed mutant inferred from homology. Arrows point from WT effector to mutant form of the same effector.

(**B**) Scatter plots illustrating gene-by-gene comparisons of percent change in MFI versus the DMD neutral control for each of the 20 effectors tested in (**A**) and **Fig. 4e**. Pearson correlation for each comparison is indicated. KRAB and p65-HSF1 controls are not included in correlation calculation.

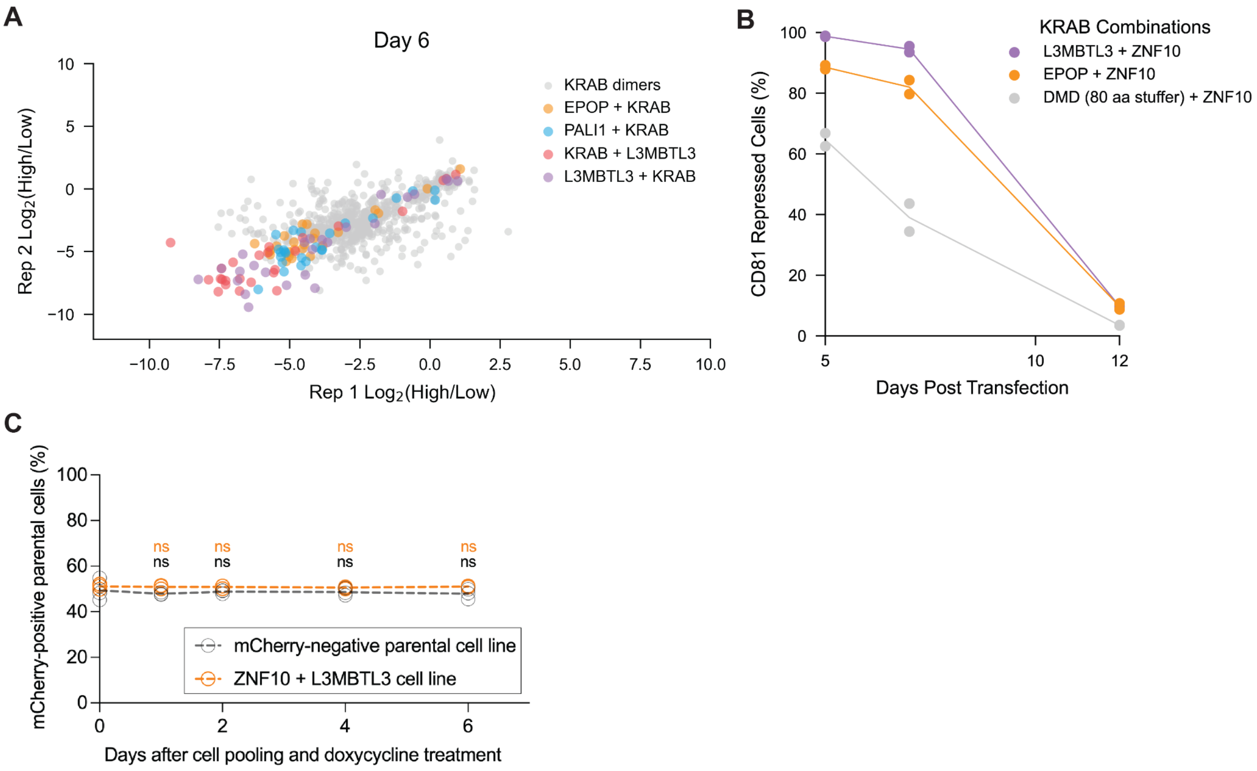

Supplementary Figure 21. Synergistic partners of KRAB
(**A**) Scatter plot of enrichment scores of selected KRAB combinations from Library 1 at Day 6. (**B**) Arrayed validation of synergistic KRAB combinations including L3MBTL3 and EPOP. DMD is an 80 aa fragment stuffer that has been validated to be well expressed and does not have transcriptional perturbation activity from a previous study[24] (n = 2 independent replicates). (**C**) Cell growth competition assay[94] (details in Methods section) comparing mCherry-positive proportions between the mCherry-negative parental cell line (gray) and ZNF10 + L3MBTL3 expressing cell line (orange) over 6 days after cell pooling and doxycycline treatment (n = 3 independent replicates). Statistical significance was determined using paired t-test comparing each time point against the first time point (ns: not significant).

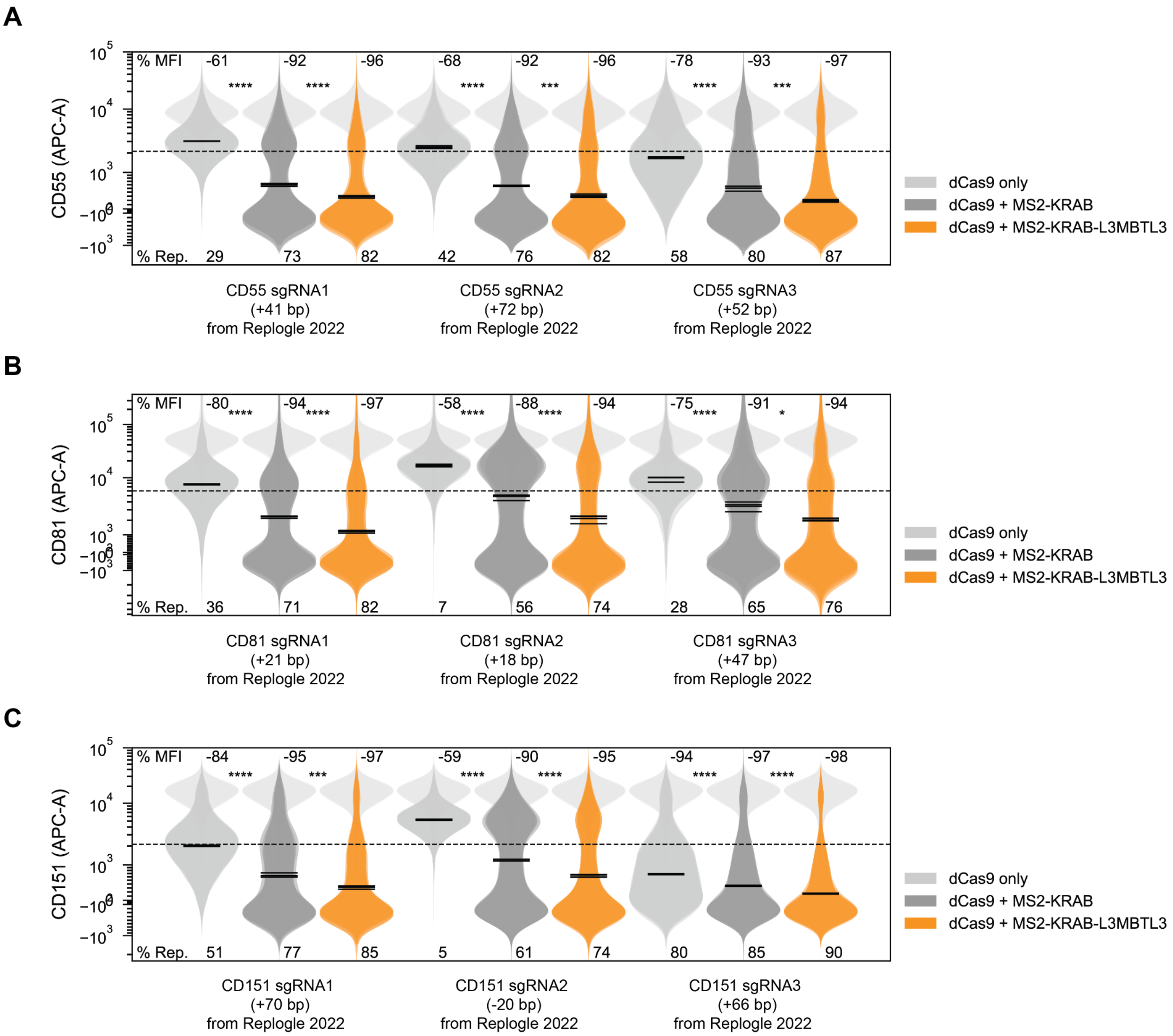

Supplementary Figure 22. Comparison of KRAB and KRAB + L3MBTL3 with highly potent TSS-downstream sgRNAs

Violin plots of target gene expression 5 days following lentiviral transduction with 1X sgRNA and doxycycline induction (1~2 µg/mL) of effector components. Stable K562 cell lines express either stdMCP-KRAB or stdMCP-KRAB-L3MBTL3 from a TRE3G promoter as described in the main text. Color scheme: dCas9 with on-target sgRNA (medium grey), dCas9 + stdMCP-KRAB with on-target sgRNA (dark grey), dCas9 + stdMCP-KRAB-L3MBTL3 with on-target sgRNA (orange), dCas9 + stdMCP-KRAB with non-target sgRNA (light grey). Distance between the sgRNA target site and the TSS is indicated in parentheses (**Supplementary Data 9**). Flow cytometry analysis was performed with gating on mCherry positive cells that were transduced with the guide RNA. 4 independent replicates are illustrated as translucent overlays. The geometric means of each replicate are shown as solid black lines. Dashed lines indicate the repression gate at the bottom 1% of stdMCP-KRAB with non-target sgRNA. The average percentages of repressed cells in

each condition are indicated. The average percent changes in target gene MFI are indicated, calculated relative to the stdMCP-KRAB non-target sgRNA control for each condition. Geometric means from dCas9 versus KRAB and KRAB versus KRAB-L3MBTL3 were tested for statistically significant differences using Welch's t-test. Significance levels are indicated as: *$p \leq 0.05$, **$p \leq 0.01$, ***$p \leq 0.001$, ****$p \leq 0.0001$.

(**A**) CD55 expression 5 days following lentiviral transduction with 1X sgRNA and doxycycline induction of the effector components (n = 4 independent replicates).

(**B**) CD81 expression 5 days following lentiviral transduction with 1X sgRNA and doxycycline induction of the effector components (n = 4 independent replicates).

(**C**) CD151 expression 5 days following lentiviral transduction with 1X sgRNA and doxycycline induction of the effector components (n = 4 independent replicates).

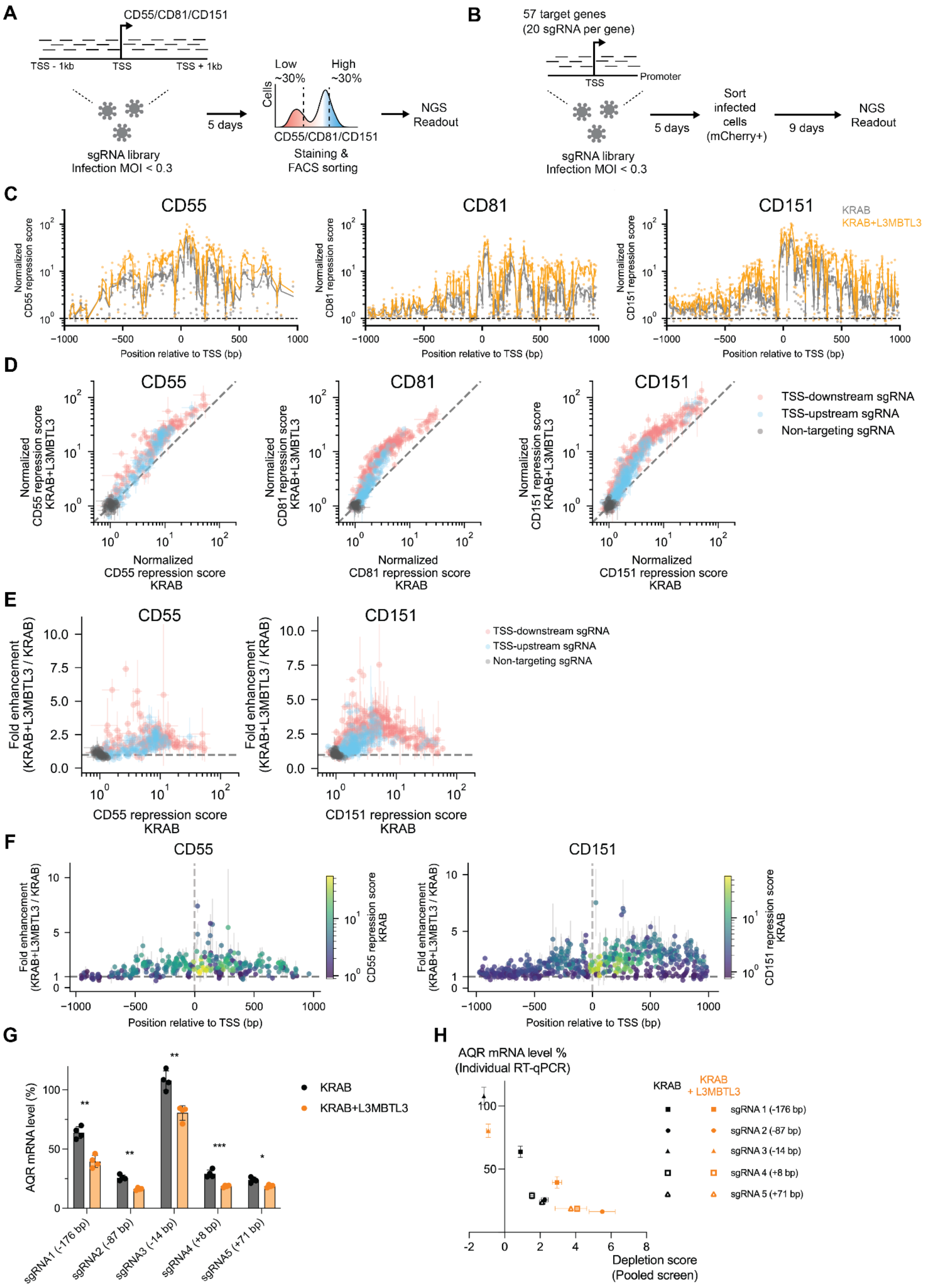
A
CD55/CD81/CD151
TSS - 1kb
TSS
TSS + 1kb
sgRNA library
Infection MOI < 0.3
5 days
Low ~30%
High ~30%
Cells
CD55/CD81/CD151
Staining & FACS sorting
NGS Readout
B
57 target genes
(20 sgRNA per gene)
TSS
Promoter
sgRNA library
Infection MOI < 0.3
5 days
Sort infected cells (mCherry+)
9 days
NGS Readout
C
CD55
CD81
CD151
KRAB
KRAB+L3MBTL3
Normalized CD55 repression score
Normalized CD81 repression score
Normalized CD151 repression score
Position relative to TSS (bp)
D
CD55
CD81
CD151
TSS-downstream sgRNA
TSS-upstream sgRNA
Non-targeting sgRNA
Normalized CD55 repression score KRAB+L3MBTL3
Normalized CD81 repression score KRAB+L3MBTL3
Normalized CD151 repression score KRAB+L3MBTL3
Normalized CD55 repression score KRAB
Normalized CD81 repression score KRAB
Normalized CD151 repression score KRAB
E
CD55
CD151
Fold enhancement (KRAB+L3MBTL3 / KRAB)
TSS-downstream sgRNA
TSS-upstream sgRNA
Non-targeting sgRNA
CD55 repression score KRAB
CD151 repression score KRAB
F
CD55
CD151
Fold enhancement (KRAB+L3MBTL3 / KRAB)
CD55 repression score KRAB
CD151 repression score KRAB
Position relative to TSS (bp)
G
AQR mRNA level (%)
KRAB
KRAB+L3MBTL3
sgRNA1 (-176 bp)
sgRNA2 (-87 bp)
sgRNA3 (-14 bp)
sgRNA4 (+8 bp)
sgRNA5 (+71 bp)
H
AQR mRNA level % (Individual RT-qPCR)
KRAB
KRAB + L3MBTL3
sgRNA 1 (-176 bp)
sgRNA 2 (-87 bp)
sgRNA 3 (-14 bp)
sgRNA 4 (+8 bp)
sgRNA 5 (+71 bp)
Depletion score (Pooled screen)

Supplementary Figure 23. Comparison of KRAB and KRAB + L3MBTL3 across CD55/CD81/CD151 promoter tiling screens and CRISPRi growth screens

(**A**) Schematic of the CD55/CD81/CD151 promoter tiling screen. sgRNA libraries targeting 2 kb regions spanning the TSS of CD55, CD81, and CD151 were designed along with non-targeting sgRNAs and transduced into K562 cells expressing the corresponding effectors. Cells were stained for surface markers, gated on mCherry positive (transduced) cells, and sorted into high and low target gene expression populations. sgRNA enrichment was quantified by NGS (see Methods for details).

(**B**) Schematic of the K562 growth screen. An sgRNA library targeting promoters of 57 genes with varying essentiality in K562 cells was designed, with 20 sgRNAs per gene spanning regions upstream and downstream of the TSS, along with non-targeting sgRNAs. sgRNA enrichment or depletion was quantified after 14 days of culture post-infection (see Methods for details).

(**C**) Normalized repression scores are plotted as a function of sgRNA target position, spanning 1 kb upstream to 1 kb downstream of the TSS. Individual data points represent mean repression scores across replicates for sgRNAs targeting each position using KRAB (grey) or KRAB + L3MBTL3 (orange) effector domains. Repression scores were normalized by dividing each targeting sgRNA score by the mean non-targeting sgRNA score, such that non-targeting controls equal 1 (dashed horizontal line). Solid lines show 3-position centered moving averages for each effector. Y-axis is displayed on a logarithmic scale.

(**D**) Scatter plots showing normalized repression scores for individual sgRNAs targeting regions upstream (blue) or downstream (pink) of TSS, with non-targeting control sgRNAs shown in grey. Each point represents the mean normalized repression score across replicates, with error bars indicating standard deviation. The dashed diagonal line (y = x) indicates equal repression efficiency between the two effector systems; points above the line represent sgRNAs with enhanced repression by KRAB + L3MBTL3 relative to KRAB alone. Both axes are displayed on a logarithmic scale.

(**E**) Scatter plots showing the fold enhancement (KRAB + L3MBTL3 normalized repression score / KRAB normalized repression score) for individual sgRNAs targeting regions upstream (blue) or downstream (pink) of the transcription start site (TSS), with non-targeting control sgRNAs shown in grey. Each point represents the mean fold enhancement across replicates, with error bars indicating standard deviation. The dashed horizontal line (y = 1) indicates no enhancement; values above 1 indicate stronger repression by KRAB + L3MBTL3 relative to KRAB alone. X-axis is displayed on a logarithmic scale.

(**F**) Fold enhancement of normalized repression score (KRAB + L3MBTL3 / KRAB alone) plotted against the sgRNA cut site relative to the transcription start site (TSS). Each point represents a unique sgRNA, with color indicating the baseline repression score achieved by KRAB alone (see colorbar). Error bars represent mean ± standard deviation across two biological replicates. The dashed horizontal line at y=1 indicates no enhancement (equal repression between KRAB + L3MBTL3 and KRAB alone). The vertical dashed line marks the TSS position (0 bp).

(**G**) AQR mRNA expression measured by qRT-PCR 2 days after doxycycline induction of dCas9 and stdMCP-KRAB or stdMCP-KRAB-L3MBTL3. Stable K562 cell lines expressing either stdMCP-KRAB or stdMCP-KRAB-L3MBTL3 from a TRE3G promoter were infected with lentivirus encoding both AQR-targeting sgRNA and mCherry in doxycycline-free media

(**Supplementary Data 9**). 3 days after lentiviral infection, mCherry positive cells were sorted; 1 day after recovery, induction with 1 µg/mL doxycycline started. AQR mRNA levels were measured by qRT-PCR 44 hr after doxycycline induction (n=4 biological replicates). Mean and standard deviation are shown. Distance between the sgRNA cut site and the TSS is indicated in parentheses. AQR mRNA levels were normalized to non-target sgRNA condition, and expression levels from KRAB versus KRAB-L3MBTL3 were tested for statistically significant differences using Welch's t-test. *p ≤ 0.05, **p ≤ 0.01, ***p ≤ 0.001.

(**H**) Scatter plot of depletion score from the K562 growth screen versus AQR mRNA levels measured by qRT-PCR. Error bars represent standard deviations of 2 replicates (x-axis, growth screen) and 4 replicates (y-axis, qRT-PCR). Mean values are plotted.

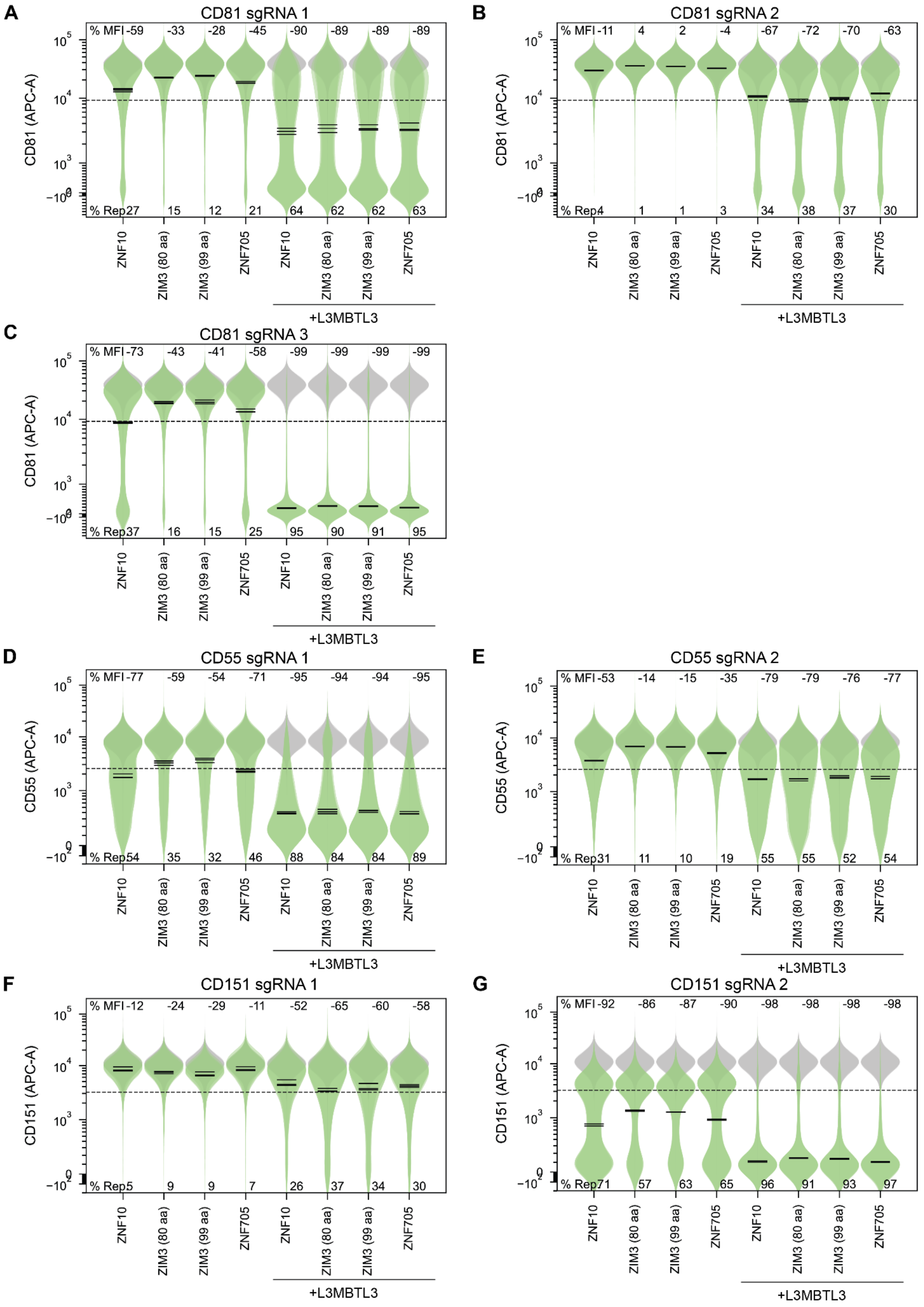
A
CD81 sgRNA 1
% MFI -59 -33 -28 -45 -90 -89 -89 -89
% Rep 27 15 12 21 64 62 62 63
CD81 (APC-A)
B
CD81 sgRNA 2
% MFI -11 4 2 -4 -67 -72 -70 -63
% Rep 4 1 1 3 34 38 37 30
C
CD81 sgRNA 3
% MFI -73 -43 -41 -58 -99 -99 -99 -99
% Rep 37 16 15 25 95 90 91 95
D
CD55 sgRNA 1
% MFI -77 -59 -54 -71 -95 -94 -94 -95
% Rep 54 35 32 46 88 84 84 89
CD55 (APC-A)
E
CD55 sgRNA 2
% MFI -53 -14 -15 -35 -79 -79 -76 -77
% Rep 31 11 10 19 55 55 52 54
F
CD151 sgRNA 1
% MFI -12 -24 -29 -11 -52 -65 -60 -58
% Rep 5 9 9 7 26 37 34 30
CD151 (APC-A)
G
CD151 sgRNA 2
% MFI -92 -86 -87 -90 -98 -98 -98 -98
% Rep 71 57 63 65 96 91 93 97
ZNF10
ZIM3 (80 aa)
ZIM3 (99 aa)
ZNF705
+L3MBTL3

Supplementary Figure 24. KRAB + L3MBTL3 synergy extends beyond the KRAB domain from ZNF10

Violin plots of target gene expression 5 days following lentiviral transduction with 1X sgRNA and doxycycline induction (1~2 μg/mL) of effector components. Stable K562 cell lines express either stdMCP-KRAB or stdMCP-KRAB-L3MBTL3 from a TRE3G promoter. Different KRAB domains from KRAB zinc finger protein paralogs (ZNF10, ZIMF3, ZNF705) were tested. ZIM3 (80 aa) denotes an exact library member in Library 1 screen. ZIM3 (99 aa) denotes 99 aa fragment characterized in the previous study[20]. Color scheme: stdMCP-KRAB or stdMCP-KRAB-L3MBTL3 with on-target sgRNA (green), stdMCP-KRAB with non-target sgRNA (light gray). Flow cytometry analysis was performed with gating on mCherry positive cells that were transduced with the guide RNA. 3 independent replicates are illustrated as translucent overlays. The geometric means of each replicate are shown as solid black lines. Dashed lines indicate the repression gate at the bottom 1% of stdMCP-KRAB with non-target sgRNA. The average percentages of repressed cells in each condition are indicated. The average percent changes in target gene MFI are indicated, calculated relative to the stdMCP-KRAB non-target sgRNA control for each condition.

(**A**) CD81 expression 5 days following lentiviral transduction with CD81 sgRNA1 and doxycycline induction of the effector components (n = 3 independent replicates).

(**B**) CD81 expression 5 days following lentiviral transduction with CD81 sgRNA2 and doxycycline induction of the effector components (n = 3 independent replicates).

(**C**) CD81 expression 5 days following lentiviral transduction with CD81 sgRNA3 and doxycycline induction of the effector components (n = 3 independent replicates).

(**D**) CD55 expression 5 days following lentiviral transduction with CD55 sgRNA1 and doxycycline induction of the effector components (n = 3 independent replicates).

(**E**) CD55 expression 5 days following lentiviral transduction with CD55 sgRNA2 and doxycycline induction of the effector components (n = 3 independent replicates).

(**F**) CD151 expression 5 days following lentiviral transduction with CD151 sgRNA1 and doxycycline induction of the effector components (n = 3 independent replicates).

(**G**) CD151 expression 5 days following lentiviral transduction with CD151 sgRNA2 and doxycycline induction of the effector components (n = 3 independent replicates).

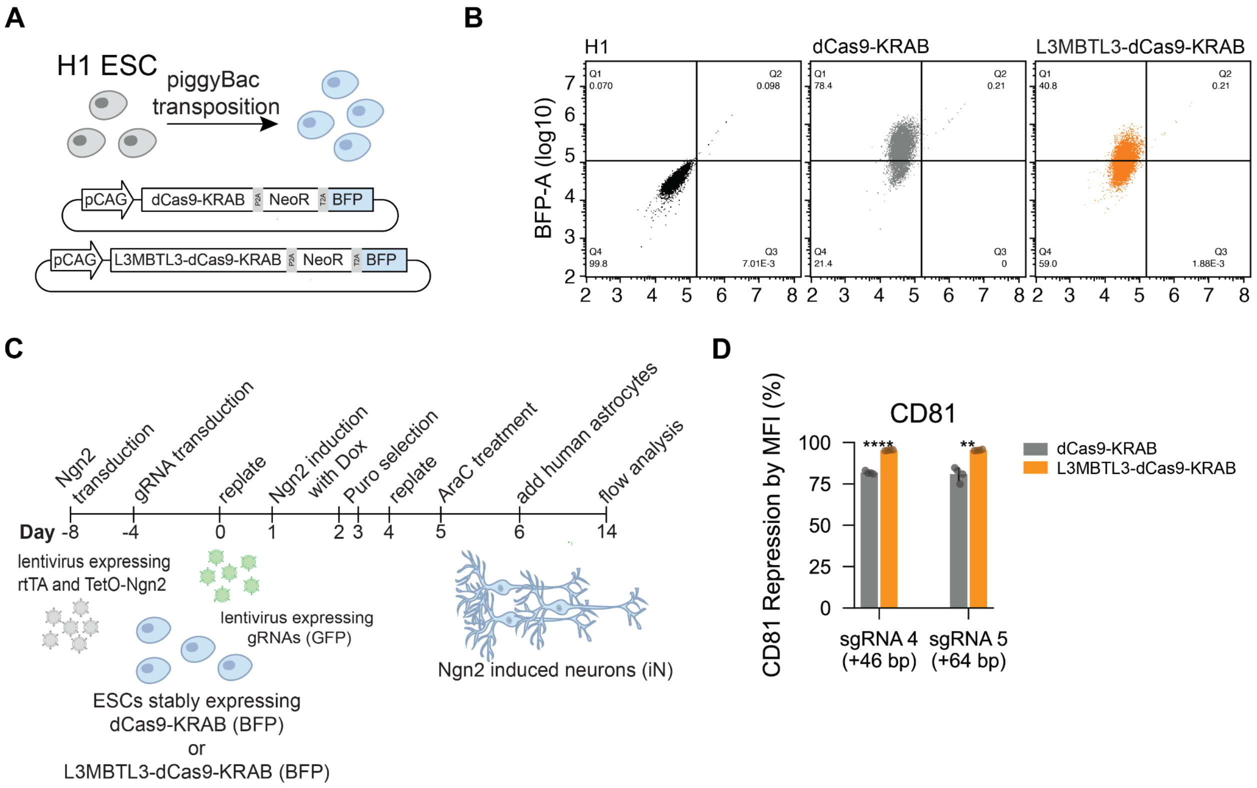

Supplementary Figure 25. Direct fusion of KRAB + L3MBTL3 to dCas9 in NGN2-induced neurons

(A) Schematic of plasmids used for the generation of ESC lines stably expressing dCas9-KRAB or L3MBTL3-dCas9-KRAB. BFP and neomycin resistance gene are expressed under the control of the CAG promoter.

(B) Representative FACS plots of BFP level in control H1 cells and H1 cells engineered to constitutively express dCas9-KRAB (BFP) or L3MBTL3-dCas9-KRAB (BFP).

(C) Timeline for testing efficiency of dCas9-KRAB (BFP) and L3MBTL3-dCas9-KRAB (BFP) in ESC-induced neurons.

(D) CD81 repression by dCas9-KRAB and L3MBTL3-dCas9-KRAB in induced neurons. Knockdown efficiency was calculated by normalizing APC-CD81-MFI in cells transduced with CD81-targeting guides to APC-CD81-MFI in cells transduced with non-targeting guides (**Supplementary Data 9**). Means from dCas9-KRAB versus L3MBTL3-dCas9-KRAB were tested for statistically significant differences using Welch's t-test. Significance levels are indicated as: *p ≤ 0.05, **p ≤ 0.01, ***p ≤ 0.001, ****p ≤ 0.0001.

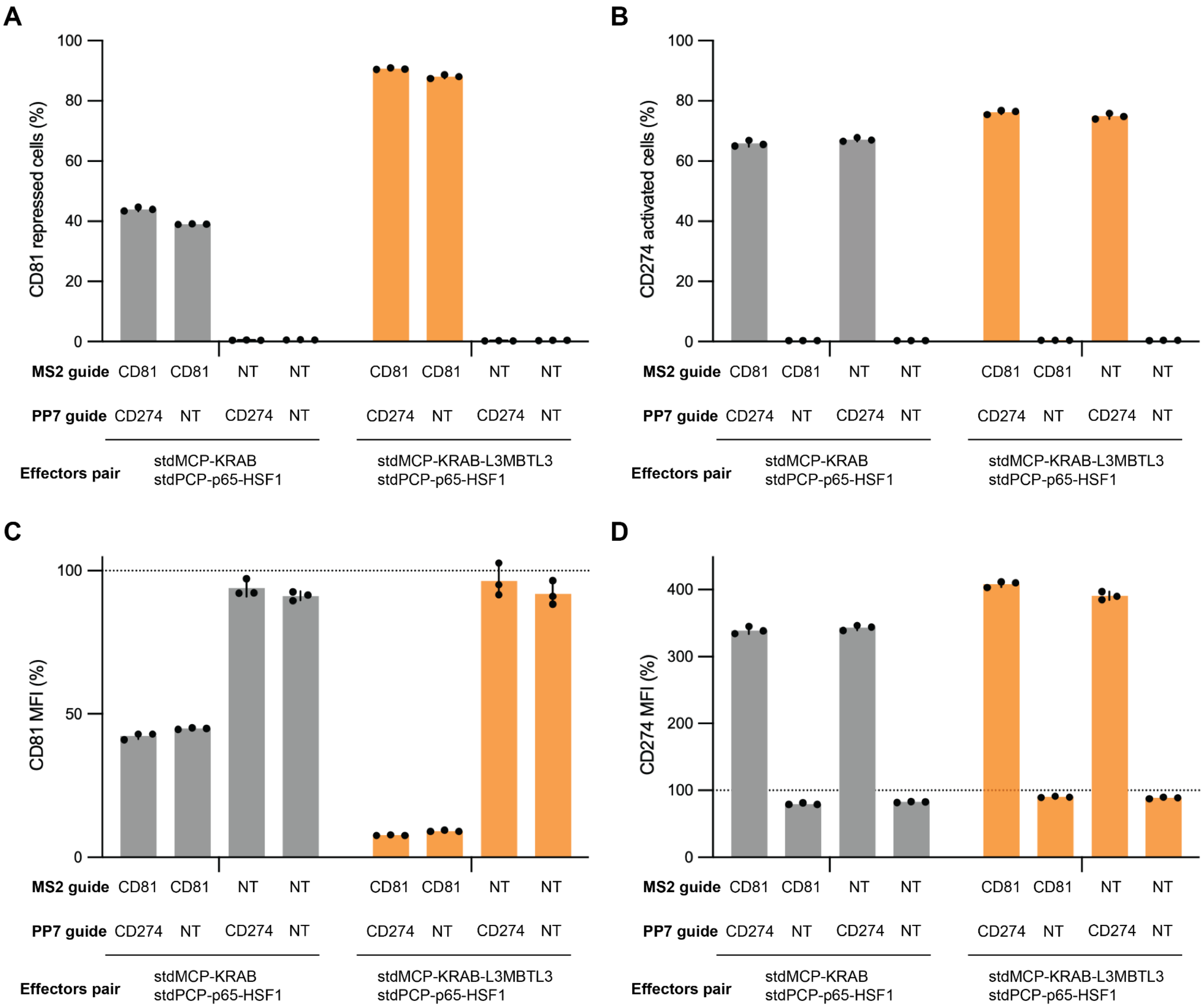

Supplementary Figure 26. Quantification of dual-directional perturbations by MS2/PP7 system

(**A**) Percentage of CD81 repressed cells 5 days post-infection of dual MS2/PP7 sgRNAs to the respective cell lines. Error bars indicate the mean ± standard deviation between 3 independent replicates.

(**B**) Percentage of CD274 activated cells 5 days post-infection of dual MS2/PP7 sgRNAs to the respective cell lines. Error bars indicate the mean ± standard deviation between 3 independent replicates.

(**C**) Mean fluorescence intensity (MFI) of CD81 repressed cells 5 days post-infection of dual MS2/PP7 sgRNAs to the respective cell lines. Expression level was normalized to control cells with no effectors. Error bars indicate the mean ± standard deviation between 3 independent replicates.

(**D**) Mean fluorescence intensity (MFI) of CD274 activated cells 5 days post-infection of dual MS2/PP7 sgRNAs to the respective cell lines. Expression level was normalized to control cells with no effectors. Error bars indicate the mean ± standard deviation between 3 independent replicates.

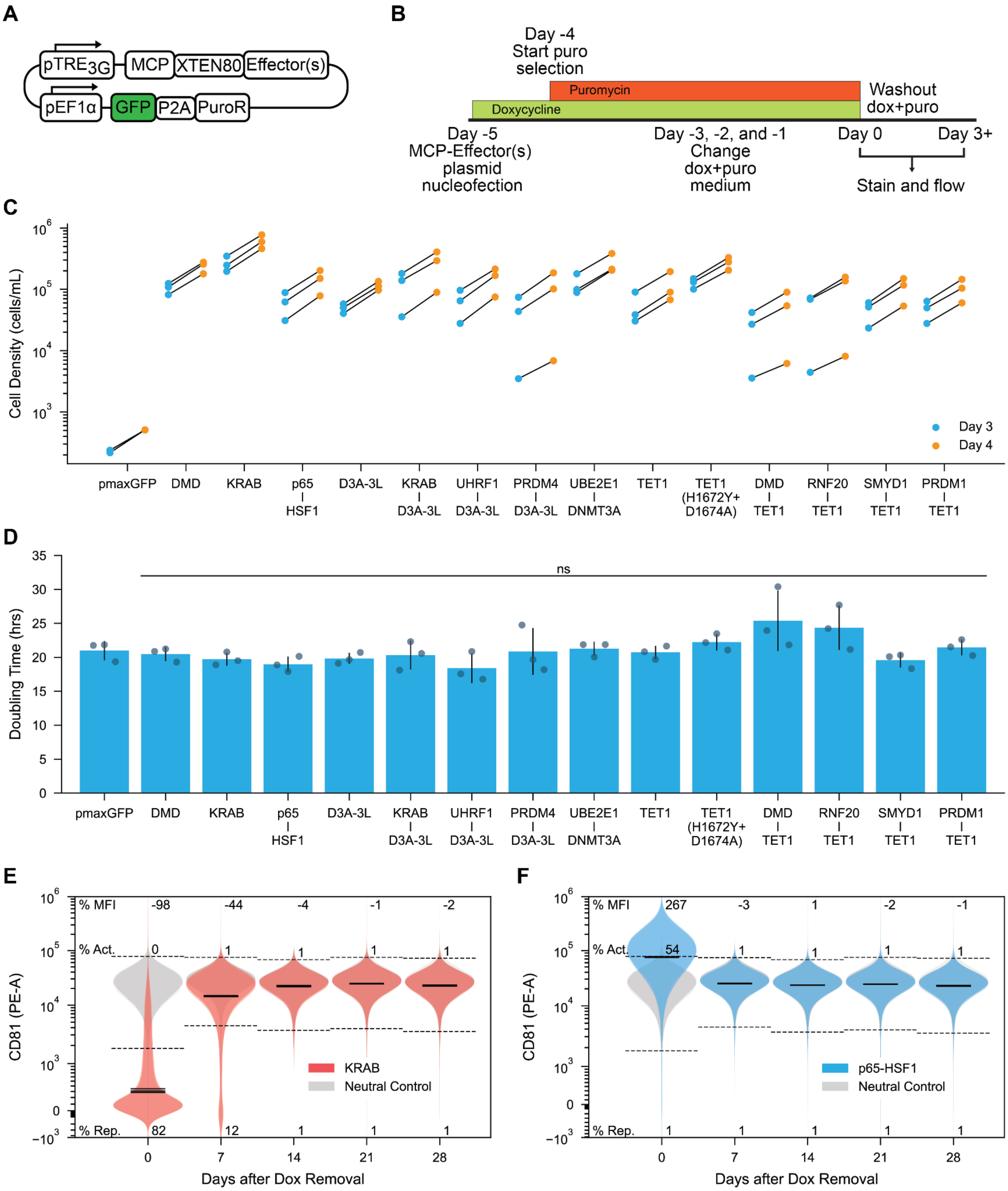

Supplementary Figure 27. Individual validation assay for long-term perturbations and doubling time measurements

(**A**) Schematic of individual validation plasmids for nucleofection. MCP-XTEN80-Effector(s) are expressed by doxycycline-inducible TRE3G promoter. GFP and puromycin resistance genes are expressed by EF1α promoter.

(**B**) Experimental timeline for measuring long-term perturbations. Refer to methods for detailed description.
(**C**) Cell density measurements at day 3 and day 4 post-doxycycline washout for key long-term effectors. 3 independent replicates are shown. Only the pmaxGFP condition does not contain the PuroR gene.
(**D**) Doubling times calculated from cell density measurements. Error bars represent mean ± standard deviation of 3 independent replicates. Significance testing for each effector was performed using a Welch's t-test, where no effectors were significantly different from the pmaxGFP condition.
(**E**) Violin plot timecourse of CD81 expression following 5 days transient recruitment of MCP-KRAB, shown in red. MCP-DMD neutral condition colored in gray. 3 independent replicates are illustrated as translucent overlays. The geometric means of each replicate are shown as solid black lines. Dashed lines indicate repression and activation gates, defined as 1st and 99th percentile of neutral condition. The average percentage of each gated population is indicated, and the average percent changes in CD81 MFI versus the neutral DMD condition are indicated.
(**F**) Violin plot timecourse of CD81 expression following 5 days transient recruitment of MCP-p65-HSF1, shown in blue. MCP-DMD neutral condition colored in gray. 3 independent replicates are illustrated as translucent overlays. The geometric means of each replicate are shown as solid black lines. Dashed lines indicate repression and activation gates, defined as 1st and 99th percentile of neutral condition. The average percentage of each gated population is indicated. The average percent changes in CD81 MFI versus the neutral DMD condition are indicated.

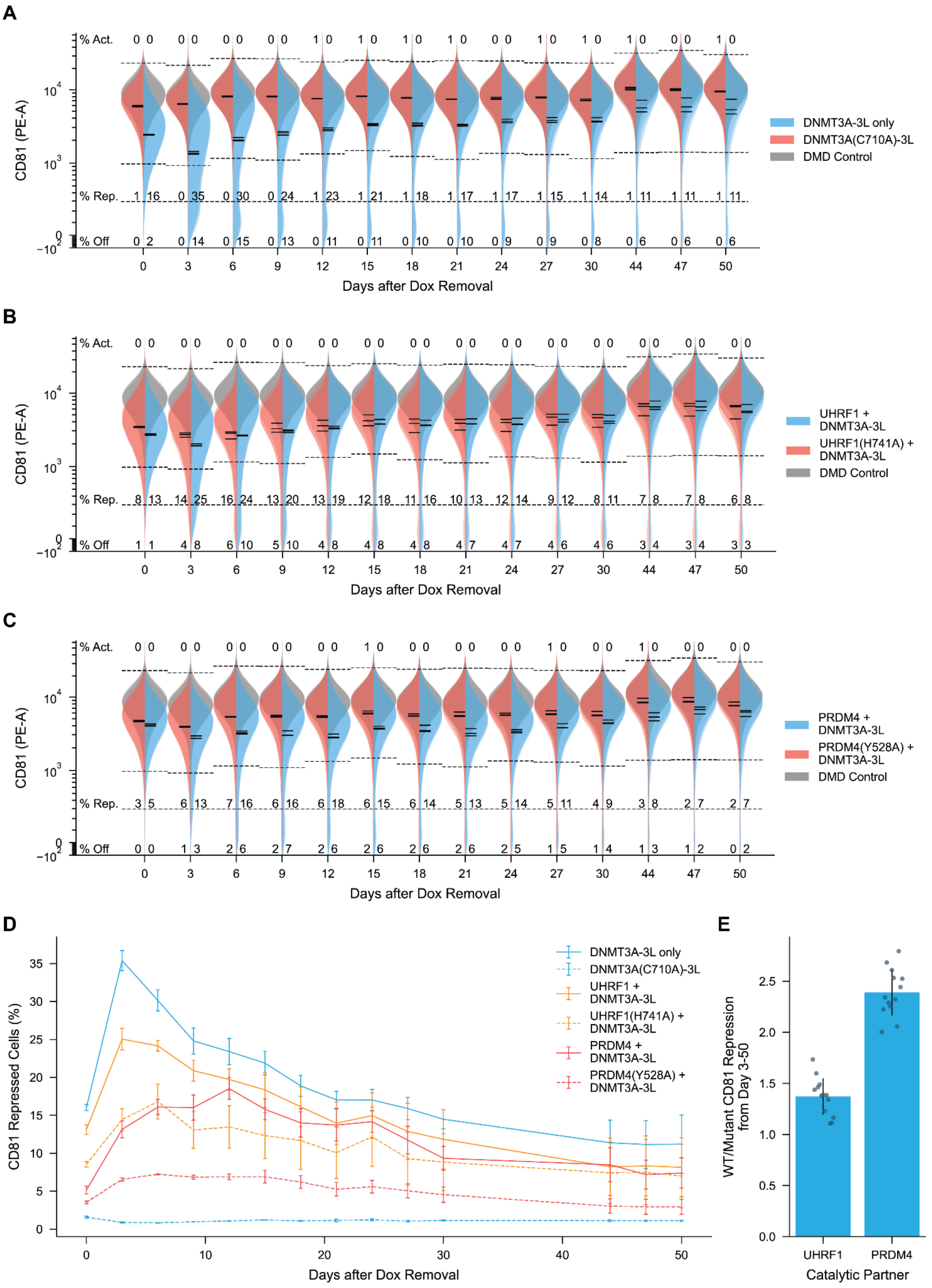
A
% Act. 0 0 0 0 0 0 0 0 1 0 1 0 1 0 1 0 0 0 1 0 1 0 1 0 0 0 1 0
% Rep. 1 16 0 35 0 30 0 24 1 23 1 21 1 18 1 17 1 17 1 15 1 14 1 11 1 11 1 11
% Off 0 2 0 14 0 15 0 13 0 11 0 11 0 10 0 10 0 9 0 9 0 8 0 6 0 6 0 6
CD81 (PE-A)
Days after Dox Removal
0 3 6 9 12 15 18 21 24 27 30 44 47 50
DNMT3A-3L only
DNMT3A(C710A)-3L
DMD Control
B
% Act. 0 0 0 0 0 0 0 0 0 0 0 0 0 0 0 0 0 0 0 0 0 0 0 0 0 0 0 0
% Rep. 8 13 14 25 16 24 13 20 13 19 12 18 11 16 10 13 12 14 9 12 8 11 7 8 7 8 6 8
% Off 1 1 4 8 6 10 5 10 4 8 4 8 4 8 4 7 4 7 4 6 4 6 3 4 3 4 3 3
CD81 (PE-A)
Days after Dox Removal
UHRF1 + DNMT3A-3L
UHRF1(H741A) + DNMT3A-3L
DMD Control
C
% Act. 0 0 0 0 0 0 0 0 0 0 1 0 0 0 0 0 0 0 1 0 0 0 1 0 0 0 0 0
% Rep. 3 5 6 13 7 16 6 16 6 18 6 15 6 14 5 13 5 14 5 11 4 9 3 8 2 7 2 7
% Off 0 0 1 3 2 6 2 7 2 6 2 6 2 6 2 6 2 5 1 5 1 4 1 3 1 2 0 2
CD81 (PE-A)
Days after Dox Removal
PRDM4 + DNMT3A-3L
PRDM4(Y528A) + DNMT3A-3L
DMD Control
D
CD81 Repressed Cells (%)
Days after Dox Removal
DNMT3A-3L only
DNMT3A(C710A)-3L
UHRF1 + DNMT3A-3L
UHRF1(H741A) + DNMT3A-3L
PRDM4 + DNMT3A-3L
PRDM4(Y528A) + DNMT3A-3L
E
WT/Mutant CD81 Repression from Day 3-50
Catalytic Partner
UHRF1
PRDM4

Supplementary Figure 28. DNMT3A-3L combinations induce long-term partial silencing of CD81

(**A**) Violin plot timecourse of CD81 expression following 5 days transient recruitment of DNMT3A-3L and catalytic mutant. MCP-DMD neutral condition colored in gray. 3 independent replicates are illustrated as translucent overlays. The geometric means of each replicate are shown as solid black lines. Dashed lines indicate repression, activation, and off gate. Repression and activation gates defined as 1st and 99th percentile of neutral condition. Off gate defined as 99th percentile of unstained WT K562s. The average percentage of each population in each condition is indicated.

(**B**) Violin plot timecourse of CD81 expression following 5 days transient recruitment of UHRF1 + DNMT3A-3L and catalytic mutant of UHRF1. Described as (**A**).

(**C**) Violin plot timecourse of CD81 expression following 5 days transient recruitment of PRDM4 + DNMT3A-3L and catalytic mutant of PRDM4. Described as (**A**).

(**D**) Timecourse of CD81 repressed cells following 5 days recruitment of WT combinations and mutant combinations. Error bars denote mean ± standard deviation between 3 independent replicates.

(**E**) Average fold change in CD81 repressed cells for WT effectors versus mutant effectors in DNMT3A-3L combinations. Error bars denote mean ± standard deviation between ratios calculated from average percentages across the 50 day timecourse (n=13).

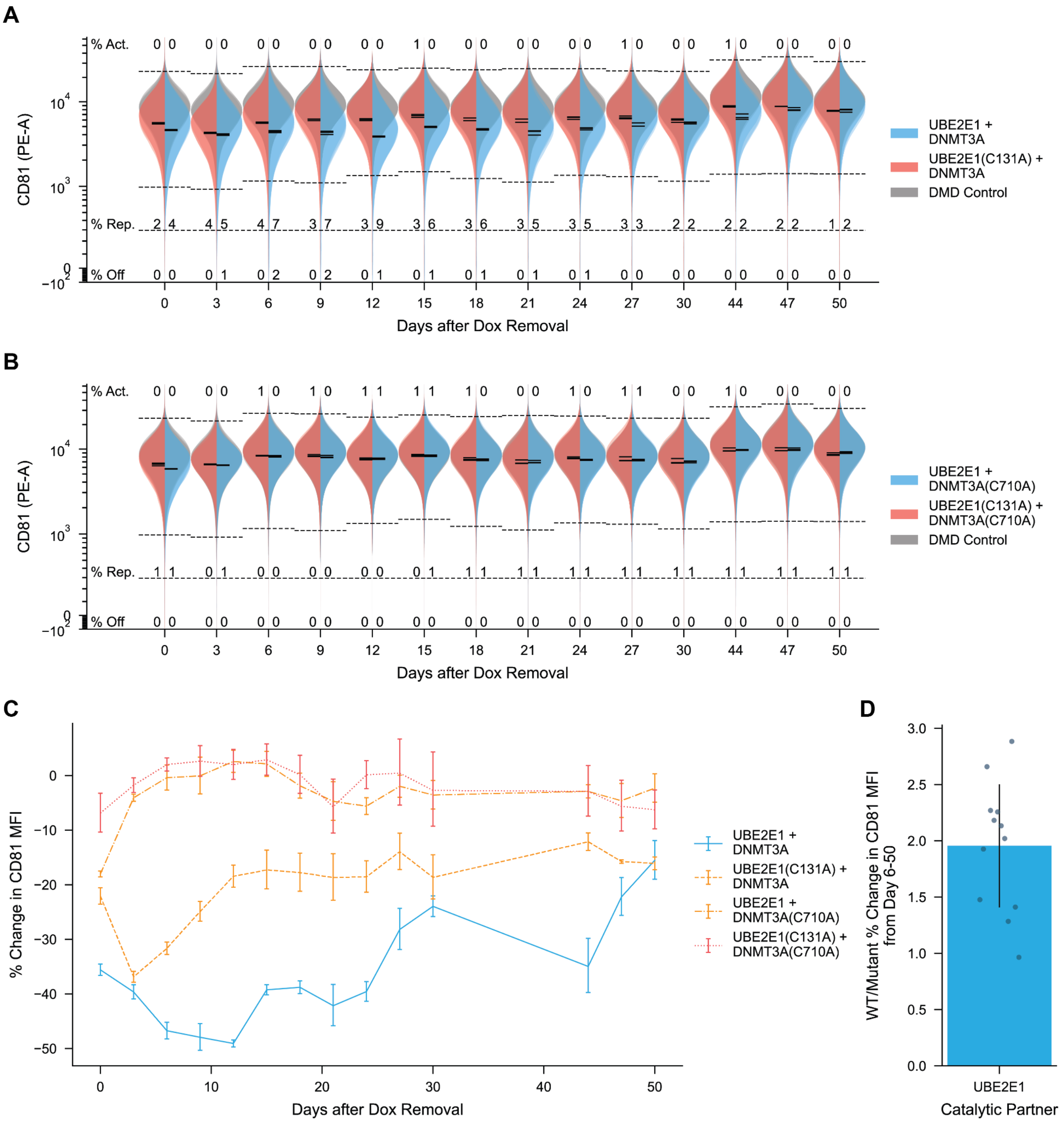

Supplementary Figure 29. UBE2E1 + DNMT3A induces long-term repression of CD81

(**A**) Violin plot timecourse of CD81 expression following 5 days transient recruitment of UBE2E1 + DNMT3A and UBE2E1(C131A) + DNMT3A. MCP-DMD neutral condition colored in gray. 3 independent replicates are illustrated as translucent overlays. The geometric means of each replicate are shown as solid black lines. Dashed lines indicate repression, activation, and off gate. Repression and activation gates defined as 1st and 99th percentile of neutral condition. Off gate defined as 99th percentile of unstained WT K562s. The average percentage of each population in each condition is indicated.

(**B**) Violin plot timecourse of CD81 expression following 5 days transient recruitment of UBE2E1 + DNMT3A(C710A) and UBE2E1(C131A) + DNMT3A(C710A). Described as (**A**).
(**C**) Timecourse of % change in CD81 MFI versus neutral DMD control following 5 days recruitment of UBE2E1 + DNMT3A and mutant combinations. Error bars denote mean ± standard deviation between 3 independent replicates.
(**D**) Average fold change in % change in CD81 MFI for UBE2E1 + DNMT3A versus UBE2E1(C131A) + DNMT3A. Error bars denote standard deviation between ratios calculated from average percentages across the 50 day timecourse (n=12).

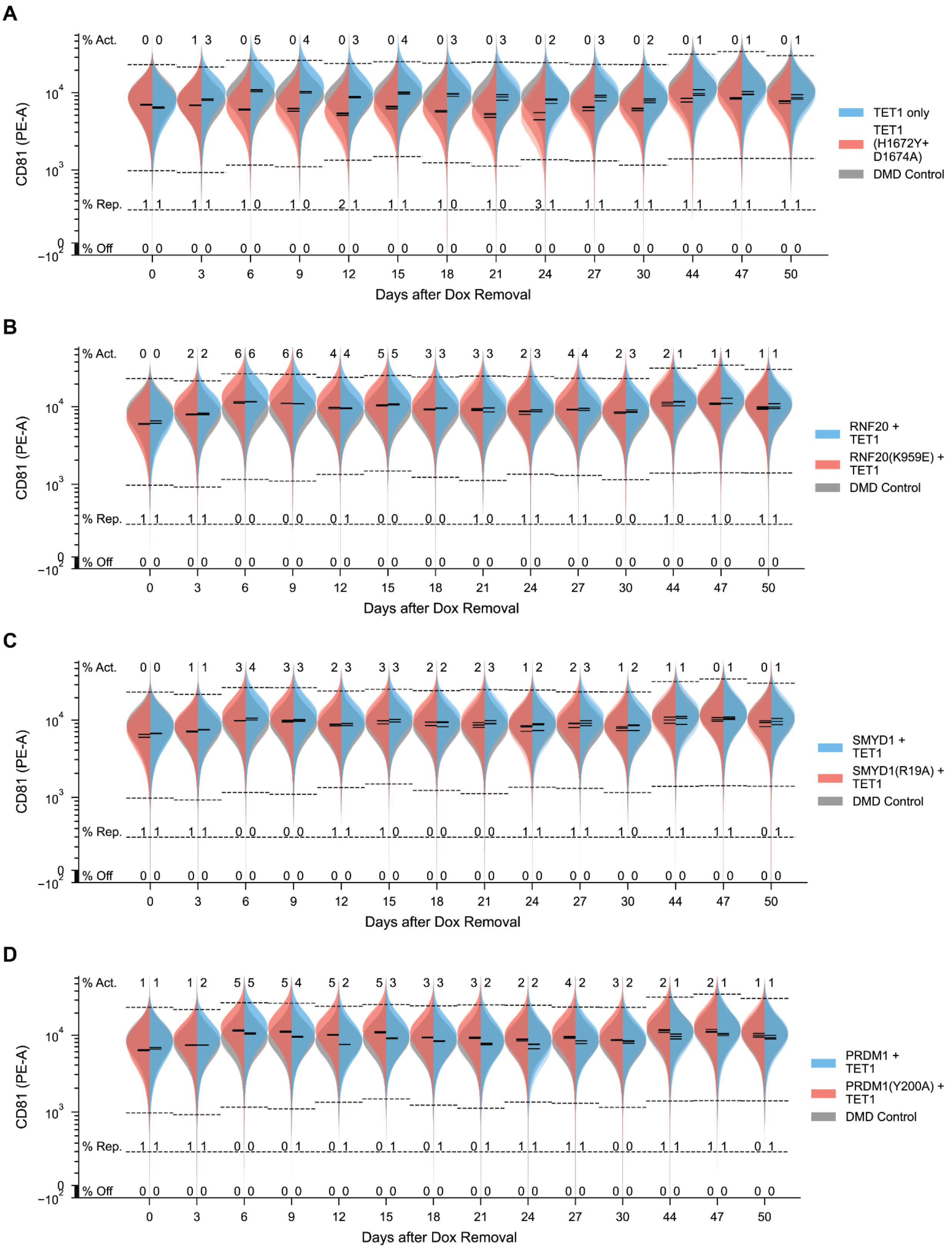
A
B
C
D
% Act.
% Rep.
% Off
CD81 (PE-A)
Days after Dox Removal
TET1 only
TET1 (H1672Y+ D1674A)
DMD Control
RNF20 + TET1
RNF20(K959E) + TET1
SMYD1 + TET1
SMYD1(R19A) + TET1
PRDM1 + TET1
PRDM1(Y200A) + TET1

Supplementary Figure 30. Violin plot timecourses of TET1 combinations

(**A**) Violin plot timecourse of CD81 expression following 5 days transient recruitment of TET1 and TET1(H1672Y+D1674A). MCP-DMD neutral condition colored in gray. 3 independent replicates are illustrated as translucent overlays. The geometric means of each replicate are shown as solid black lines. Dashed lines indicate repression, activation, and off gate. Repression and activation gates defined as 1st and 99th percentile of neutral condition. Off gate defined as 99th percentile of unstained WT K562s. The average percentage of each population in each condition is indicated.

(**B**) Violin plot timecourse of CD81 expression following 5 days transient recruitment of RNF20 + TET1 and RNF20(K959E) + TET1. Described as (**A**).

(**C**) Violin plot timecourse of CD81 expression following 5 days transient recruitment of SMYD1 + TET1 and SMYD1(R19A) + TET1. Described as (**A**).

(**D**) Violin plot timecourse of CD81 expression following 5 days transient recruitment of PRDM1 + TET1 and PRDM1(Y200A) + TET1. Described as (**A**).

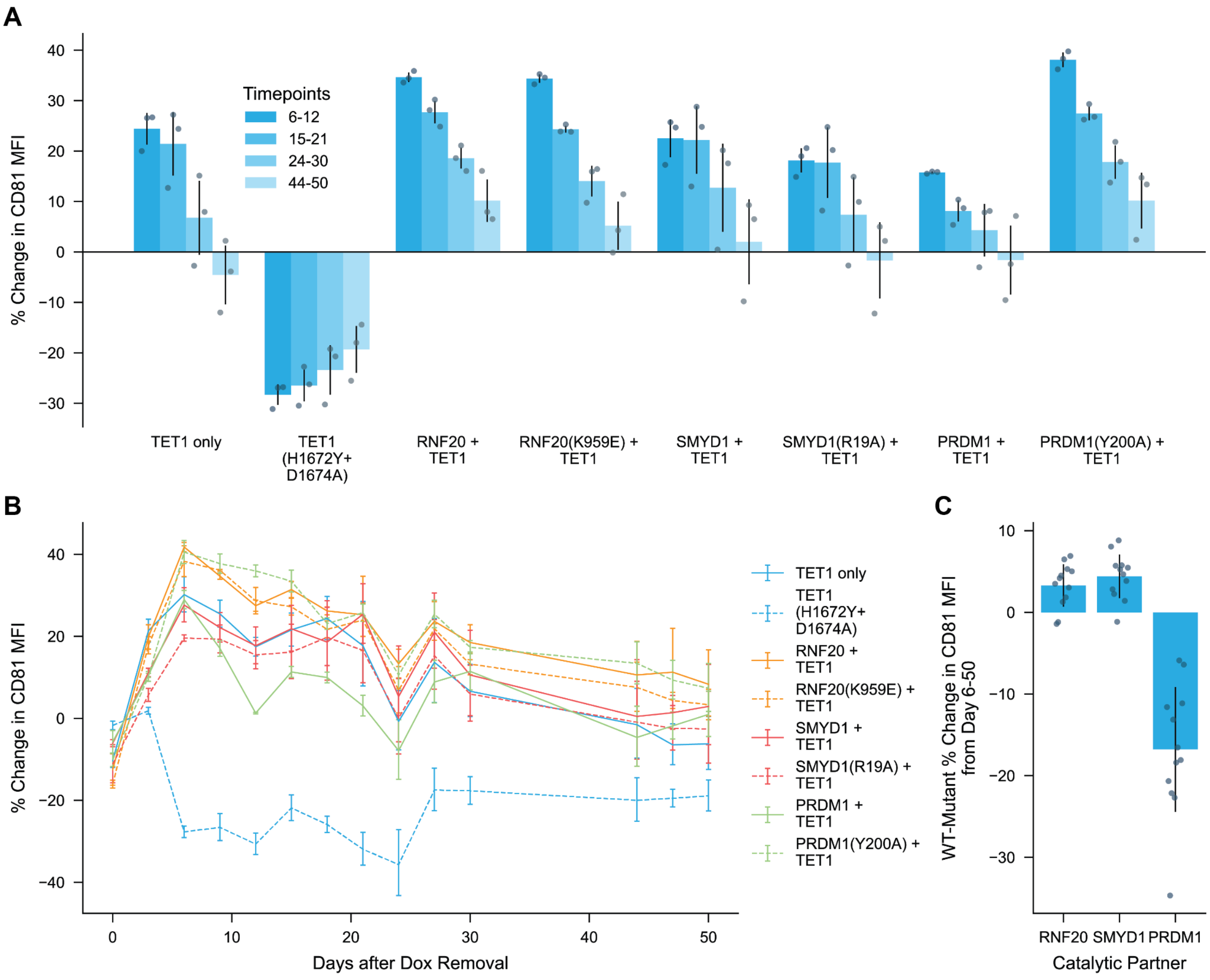

Supplementary Figure 31. TET1 combinations induce long-term activation of CD81

(**A**) Timecourse of percent change in CD81 MFI versus neutral DMD control following nucleofection of MCP-TET1 combination encoding plasmids and 5 days recruitment of dCas9/MS2 complex. Day 0 indicates the day of doxycycline washout. Each bar represents 3 timepoints collected within the specified days. The bar heights represent the average perturbation across the timepoints, and the error bars represent the standard deviation between 3 independent replicates.

(**B**) Timecourse of % change in CD81 MFI versus neutral DMD control following 5 days recruitment of TET1 combinations and mutants. Error bars denote mean ± standard deviation between 3 independent replicates.

(**C**) Average difference in % change in CD81 MFI for WT effectors versus mutant effectors in TET1 combinations. Error bars denote standard deviation between differences calculated from average percentages across the 50 day timecourse (n=12).

Supplementary Note 1

Comparison of original synergy scores with residual-based synergy scores using homotypic pairs as an alternative metric

We developed an alternative residual-based synergy metric for heterotypic pairs using homotypic pair measurements as the baseline. Unlike the original synergy score, which uses marginal scores to define the expected additive interval, this approach derives synergy scores from residuals of a regression fit between observed heterodimer enrichment and the average of the two corresponding homodimer measurements, with context-dependent sign adjustment applied to maintain consistent biological interpretation (**Supplementary Fig. 13a**).

This alternative metric correlated well with the original synergy score for Library 1 ($r = 0.787$; **Supplementary Fig. 13b**). For Library 2, the absolute values of both metrics showed strong correlation ($r = 0.747$; **Supplementary Fig. 13c**), however, raw values comprised two distinct populations: a majority showing moderate positive correlation ($n = 34812$, $r = 0.589$) and a small subset exhibiting strong negative correlation ($n = 693$, $r = -0.896$; **Supplementary Fig. 13d**). We hypothesized that this anti-correlated population arises from the sign adjustment procedure, which inverts the residual sign when the expected value is negative to maintain consistent biological interpretation of synergy scores. Restricting analysis to pairs with expected values far from zero (|sum of marginal scores| ≥ 0.3 and |average of homodimer enrichment scores| ≥ 1) recovered strong correlation ($r = 0.918$; **Supplementary Fig. 13e**), while the majority of anti-correlated pairs were found among those with expected values near zero (**Supplementary Fig. 13f**). Because the sign adjustment procedure is sensitive to small deviations when expected values are near zero, we flagged pairs falling outside the expected distribution (> 4.5σ from the regression line) in the supplementary data tables to aid interpretation (**Supplementary Data 4**).

Supplementary Note 2

Synergistic interaction between the H2AK119ub1 reader and a subset of PRC1 recruiters

Recognition of epigenetic modifications by a reader domain is a crucial step in establishing epigenetic feedback loops, representing a fundamental aspect of epigenetic interactions. Specifically, PRC1 and PRC2, responsible for installing H2AK119ub1 and H3K27me3, respectively, are well-known for their intricate feedback mechanisms. These mechanisms involve the spreading of a single epigenetic modification or cross-talk between two distinct modifications[4], all of which require the recognition of epigenetic modifications by the reader domain[6]. By focusing on the sub-heatmap defined by recruiters of PRC1 and PRC2 on the N-terminus and readers of epigenetic modifications on the C-terminus, we were able to notice a prominent signal that emerged at the intersection of a subset of PRC1 recruiters and the H2AK119ub1 reader (**Supplementary Fig. 16a**). Also, the H2AK119ub1 reader domain was nominated as the strongest synergistic C-terminus partner of PRC1 recruiters with q-value less

than 1 (excluding the KRAB family), although the q-value was higher than the threshold of 0.05 (**Supplementary Fig. 16b**).

To validate these combinations between PRC1 recruiters and the H2AK119ub1 reader, we tested several PRC1 recruiter domains encompassing domains from canonical PRC1 (CBX2/CBX7) and variant PRC1 (RYBP), in combination with the readers of distinct histone modifications (H2AK119ub1 by the RYBP Ranbp2-ZnF domain, H3K27me by the CBX6 Chromo domain, H3K9me by the CBX5 Chromo domain, and H3K4me by the ING1 PHD domain) (**Supplementary Fig. 16c**). After 4 days of recruitment, our results confirmed that only the combinations containing the H2AK119ub1 reader domain from RYBP were sufficient to induce substantial gene silencing (CBX2 + H2AK119ub1 reader: 12.7%, CBX7 + H2AK119ub1 reader: 59.7%, RYBP + H2AK119ub1 reader: 60.5% CD81 repressed cells), irrespective of whether the PRC1 component originated from the canonical or variant complex (**Supplementary Fig. 16d**). Interestingly, the validated fusion protein containing the PRC1 recruiter domain and H2AK119ub1 reader domain from RYBP essentially reconstitutes full-length RYBP with some truncations (**Supplementary Fig. 16e**). Variant PRC1 (vPRC1) comprising RYBP subunits has the unique ability to both read and write the H2AK119ub1 mark owing to the H2AK119ub1 reader domains found within RYBP protein[37]. This ability to read and write the same epigenetic modifications has been speculated as an efficient strategy for propagating silencing marks to adjacent regions, suggesting that interactions detected from our HTS experiment may recapitulate a natural architecture inherent within the PRC1 complex[37].
