# Supplementary material for: A combinatorial domain screening platform reveals epigenetic effector interactions for transcriptional perturbation
Source: Nat Commun. 2026 Apr 24;17:5697. doi: 10.1038/s41467-026-72227-9 (PMC13319239; doi:10.1038/s41467-026-72227-9)
Supplement: Supplementary file 2 — Description of Additional Supplementary Files [file 41467_2026_72227_MOESM2_ESM.pdf]

Description of Additional Supplementary Files

File Name: Supplementary Data 1
Description: Library 1 members and sequences

File Name: Supplementary Data 2
Description: Library 2 members and sequences

File Name: Supplementary Data 3
Description: Library 1 HTS results

File Name: Supplementary Data 4
Description: Library 2 HTS results

File Name: Supplementary Data 5
Description: Catalytic mutations

File Name: Supplementary Data 6
Description: Library 1 synergy scores

File Name: Supplementary Data 7
Description: Library 2 synergy scores

File Name: Supplementary Data 8
Description: Validation of KRAB + L3MBTL3 combination by CRISPRi screens

File Name: Supplementary Data 9
Description: Plasmid, primer, and guide sequences

File Name: Supplementary Data 10
Description: Library 1 individual validation details

File Name: Supplementary Data 11
Description: Analysis code

a. supduplex2fastq.sh
b. bc_extraction.py
c. count_barcodes.py
d. Minimap_batch_Lib1.py
e. R1_Forward_P5_301cycles.fasta
f. R2_Reverse_P7_301cycles.fasta
