# Supplementary material for: A combinatorial domain screening platform reveals epigenetic effector interactions for transcriptional perturbation
Source: Nat Commun. 2026 Apr 24;17:5697. doi: 10.1038/s41467-026-72227-9 (PMC13319239; doi:10.1038/s41467-026-72227-9)
Supplement: Supplementary file 14 — Reporting Summary [file 41467_2026_72227_MOESM14_ESM.pdf]

nature portfolio

Corresponding author(s): Patrick D. Hsu, Silvana Konermann

Last updated by author(s): Mar 23, 2026

Reporting Summary

Nature Portfolio wishes to improve the reproducibility of the work that we publish. This form provides structure for consistency and transparency in reporting. For further information on Nature Portfolio policies, see our Editorial Policies and the Editorial Policy Checklist.

Statistics

For all statistical analyses, confirm that the following items are present in the figure legend, table legend, main text, or Methods section.

n/a	Confirmed	
☐	☒	The exact sample size (*n*) for each experimental group/condition, given as a discrete number and unit of measurement
☐	☒	A statement on whether measurements were taken from distinct samples or whether the same sample was measured repeatedly
☐	☒	The statistical test(s) used AND whether they are one- or two-sided<br>*Only common tests should be described solely by name; describe more complex techniques in the Methods section.*
☐	☒	A description of all covariates tested
☐	☒	A description of any assumptions or corrections, such as tests of normality and adjustment for multiple comparisons
☐	☒	A full description of the statistical parameters including central tendency (e.g. means) or other basic estimates (e.g. regression coefficient) AND variation (e.g. standard deviation) or associated estimates of uncertainty (e.g. confidence intervals)
☐	☒	For null hypothesis testing, the test statistic (e.g. *F*, *t*, *r*) with confidence intervals, effect sizes, degrees of freedom and *P* value noted<br>*Give P values as exact values whenever suitable.*
☐	☒	For Bayesian analysis, information on the choice of priors and Markov chain Monte Carlo settings
☐	☒	For hierarchical and complex designs, identification of the appropriate level for tests and full reporting of outcomes
☐	☒	Estimates of effect sizes (e.g. Cohen's *d*, Pearson's *r*), indicating how they were calculated

Our web collection on statistics for biologists contains articles on many of the points above.

Software and code

Policy information about availability of computer code

Data collection	Attune NxT Software v3.1, NextSeq System Suite v2.2.0, BD FACSDiva v9.0, MinKNOW GUI v5.7.14, MinKNOW Standalone Release for Mk1B 23.04.6
Data analysis	CHOPCHOP v3.0.0, Geneious Prime v2022.1.1, DnaChisel v3.2.8, Biopython v1.79, Dorado v0.2.4, duplex-tools v0.3.2, Cutadapt v4.4, Minimap2 v2.26, SeqFu v1.17.1, Flowjo v10.7.1, GraphPad Prism v9.3.1, CytoFlow v1.2, matplotlib v3.7.3, pandas v2.0.1<br>Custom codes written to analyze high-throughput screening data is available at https://github.com/hsulab-arc/COMBINE.

For manuscripts utilizing custom algorithms or software that are central to the research but not yet described in published literature, software must be made available to editors and reviewers. We strongly encourage code deposition in a community repository (e.g. GitHub). See the Nature Portfolio guidelines for submitting code & software for further information.

Data

Policy information about availability of data

All manuscripts must include a data availability statement. This statement should provide the following information, where applicable:

- Accession codes, unique identifiers, or web links for publicly available datasets
- A description of any restrictions on data availability
- For clinical datasets or third party data, please ensure that the statement adheres to our policy

Illumina and Nanopore sequencing datasets generated in this study have been deposited in the NCBI Sequence Read Archive under BioProject PRJNA1143488 [https://www.ncbi.nlm.nih.gov/bioproject/PRJNA1143488]. Source data are provided as a Source Data file. All data are publicly available without restrictions.

Research involving human participants, their data, or biological material

Policy information about studies with human participants or human data. See also policy information about sex, gender (identity/presentation), and sexual orientation and race, ethnicity and racism.

Reporting on sex and gender	*Use the terms sex (biological attribute) and gender (shaped by social and cultural circumstances) carefully in order to avoid confusing both terms. Indicate if findings apply to only one sex or gender; describe whether sex and gender were considered in study design; whether sex and/or gender was determined based on self-reporting or assigned and methods used. Provide in the source data disaggregated sex and gender data, where this information has been collected, and if consent has been obtained for sharing of individual-level data; provide overall numbers in this Reporting Summary. Please state if this information has not been collected. Report sex- and gender-based analyses where performed, justify reasons for lack of sex- and gender-based analysis.*
Reporting on race, ethnicity, or other socially relevant groupings	*Please specify the socially constructed or socially relevant categorization variable(s) used in your manuscript and explain why they were used. Please note that such variables should not be used as proxies for other socially constructed/relevant variables (for example, race or ethnicity should not be used as a proxy for socioeconomic status). Provide clear definitions of the relevant terms used, how they were provided (by the participants/respondents, the researchers, or third parties), and the method(s) used to classify people into the different categories (e.g. self-report, census or administrative data, social media data, etc.) Please provide details about how you controlled for confounding variables in your analyses.*
Population characteristics	*Describe the covariate-relevant population characteristics of the human research participants (e.g. age, genotypic information, past and current diagnosis and treatment categories). If you filled out the behavioural & social sciences study design questions and have nothing to add here, write "See above."*
Recruitment	*Describe how participants were recruited. Outline any potential self-selection bias or other biases that may be present and how these are likely to impact results.*
Ethics oversight	*Identify the organization(s) that approved the study protocol.*

Note that full information on the approval of the study protocol must also be provided in the manuscript.

Field-specific reporting

Please select the one below that is the best fit for your research. If you are not sure, read the appropriate sections before making your selection.

☒ Life sciences ☐ Behavioural & social sciences ☐ Ecological, evolutionary & environmental sciences

For a reference copy of the document with all sections, see nature.com/documents/nr-reporting-summary-flat.pdf

Life sciences study design

All studies must disclose on these points even when the disclosure is negative.

Sample size	The experiments described in this study were done for the first time. No pre-specified effect size could be determined a priori. In this study we used a minimum of two replicates, with excellent reproducibility between replicates.
Data exclusions	No data were excluded from the study.
Replication	For high-throughput screening experiments, 2 replicates (independent lentiviral infections) were performed per standards in functional genomics field. For individual validation experiments, a minimum of 2 replicates (transfection or nucleofection) was performed. All data supports replicability.
Randomization	Randomization was not relevant for this study. The same cell lines were used for positive and negative controls per experiment, which does not require allocating samples into experimental groups.
Blinding	Investigators were not blinded, we are a small team performing cell & molecular biology experiments.

Reporting for specific materials, systems and methods

We require information from authors about some types of materials, experimental systems and methods used in many studies. Here, indicate whether each material, system or method listed is relevant to your study. If you are not sure if a list item applies to your research, read the appropriate section before selecting a response.

Materials & experimental systems

n/a	Involved in the study
☐	☒ Antibodies
☐	☒ Eukaryotic cell lines
☒	☐ Palaeontology and archaeology
☒	☐ Animals and other organisms
☒	☐ Clinical data
☒	☐ Dual use research of concern
☒	☐ Plants

Methods

n/a	Involved in the study
☒	☐ ChIP-seq
☐	☒ Flow cytometry
☒	☐ MRI-based neuroimaging

Antibodies

Antibodies used

Library 1 HTS experiment
- APC Mouse Anti-Human CD81 antibody (Cat# 561958, BD Bioscience) dilution 1:10

Library 2 HTS experiment
- PE-conjugated CD81 monoclonal antibody (Invitrogen, MA1-10292) dilution 1:20

Library 1 individual validation, KRAB-L3MBTL3, and bidirectional perturbation experiments
- APC anti-human CD55 antibody (Cat# 311311, Biolegends) dilution 1:100, Alexa Fluor 647 Mouse Anti-Human CD58 antibody (Cat# 563567, BD Pharmingen) dilution 1:100, APC Mouse Anti-Human CD81 antibody (Cat# 561958, BD Bioscience) dilution 1:20, APC anti-human CD151 antibody (Cat# 350405, Biolegends) dilution 1:100, and Alexa Fluor 488 Mouse Anti-Human CD274 antibody (Cat# 53-5983-42, Invitrogen) dilution 1:100

Library 2 individual validation experiments
- APC CD55 antibody (Biolegends, 311311) dilution 1:100, Alexa Fluor 647 CD58 antibody (BD Pharmingen, 563567) dilution 1:100, PE CD81 antibody (Invitrogen, MA1-10292) dilution 1:100, APC CD151 antibody (Biolegends, 350405) dilution 1:100, and APC CD155 antibody (eBioscience, 2H7CD155) dilution 1:100

Validation

APC Mouse Anti-Human CD81 antibody (Cat# 561958, BD Bioscience): Validated on human peripheral blood lymphocytes.
PE-conjugated CD81 monoclonal antibody (Invitrogen, MA1-10292):Used for separation of human lymphocytes (red-filled) from neutrophil granulocytes (black-dashed) in peripheral whole blood.
APC anti-human CD55 antibody (Cat# 311311, Biolegends):Validated on human peripheral blood lymphocytes
Alexa Fluor 647 Mouse Anti-Human CD58 antibody (Cat# 563567, BD Pharmingen): Validated on human peripheral blood lymphocytes.
APC anti-human CD151 antibody (Cat# 350405, Biolegends):Validated on human peripheral blood platelets.
APC CD155 antibody (eBioscience, 2H7CD155): Validated on U937 cells.
Alexa Fluor 488 Mouse Anti-Human CD274 antibody (Cat# 53-5983-42, Invitrogen):Validated on human peripheral blood cells.

Eukaryotic cell lines

Policy information about cell lines and Sex and Gender in Research

Cell line source(s)	HEK293FT and viral production cells from Thermo Fisher, K562 from ATCC, Lenti-X from Takara Bio.
Authentication	None of the cell lines used were authenticated.
Mycoplasma contamination	Cell lines were tested negative for mycoplasma.
Commonly misidentified lines (See ICLAC register)	No commonly misidentified cell lines were used.

Plants

Seed stocks

Report on the source of all seed stocks or other plant material used. If applicable, state the seed stock centre and catalogue number. If plant specimens were collected from the field, describe the collection location, date and sampling procedures.

Novel plant genotypes

Describe the methods by which all novel plant genotypes were produced. This includes those generated by transgenic approaches, gene editing, chemical/radiation-based mutagenesis and hybridization. For transgenic lines, describe the transformation method, the number of independent lines analyzed and the generation upon which experiments were performed. For gene-edited lines, describe the editor used, the endogenous sequence targeted for editing, the targeting guide RNA sequence (if applicable) and how the editor was applied.

Authentication

Describe any authentication procedures for each seed stock used or novel genotype generated. Describe any experiments used to assess the effect of a mutation and, where applicable, how potential secondary effects (e.g. second site T-DNA insertions, mosiacism, off-target gene editing) were examined.

Flow Cytometry

Plots

Confirm that:

☒ The axis labels state the marker and fluorochrome used (e.g. CD4-FITC).

☒ The axis scales are clearly visible. Include numbers along axes only for bottom left plot of group (a 'group' is an analysis of identical markers).

☒ All plots are contour plots with outliers or pseudocolor plots.

☒ A numerical value for number of cells or percentage (with statistics) is provided.

Methodology

Sample preparation	After initial washing K562 cells with stain buffer, cells were stained with antibodies diluted in the stain buffer for 1 hour at 4° C. After staining, cells were washed twice with stain buffer before the measurement.
Instrument	Attune NxT Flow Cytometer, FACSAria Fusion Special Order Research Product
Software	Flowjo v10.7.1, CytoFlow v1.0
Cell population abundance	Since samples are single cell lines, relevant population is the entire sample excluding dead cells and doublets, often >60% of the sample
Gating strategy	Cells initially gated on FSC/SSC for single cells to exclude doublets and dead cells. BFP, GFP, mCherry, and APC gates were determined by gating on cells delivered with an empty plasmid backbone (pUC19). More detailed gating strategies are described in Supplementary Figs. 5 and 6 and Supplementary Data 10.

☒ Tick this box to confirm that a figure exemplifying the gating strategy is provided in the Supplementary Information.
