# Supplementary material for: A combinatorial domain screening platform reveals epigenetic effector interactions for transcriptional perturbation
Source: Nat Commun. 2026 Apr 24;17:5697. doi: 10.1038/s41467-026-72227-9 (PMC13319239; doi:10.1038/s41467-026-72227-9)

nature portfolio

Peer Review File

A combinatorial domain screening platform reveals epigenetic effector interactions for transcriptional perturbation

Corresponding Author: Professor Patrick Hsu

This manuscript has been previously reviewed at another journal. This document only contains information relating to versions considered at Nature Communications.

This file contains all reviewer reports in order by version, followed by all author rebuttals in order by version.

Version 0:

Reviewer comments:

Reviewer #3

(Remarks to the Author)
Moon et al. present COMBINE, a platform for high-throughput screening of domain effector pairs to assess their ability to modulate transcription. The authors note that most existing screening strategies focus on single domains, limiting our understanding of how domains interact when combined. By constructing two libraries with varying effector lengths and implementing differential sequencing strategies, they profile more than 50,000 effector-pair combinations in human cells.

Reviewer #1 raised several valuable points that I agree are important to address. It is worth noting that I have not seen pooled epigenetic effector-screening data calculated in this manner, but this study is also unique in its search for combinatorial effects. The authors responded to reviewer #1's feedback effectively in their response and incorporated clarifying text within the discussion. Thus, I support its publication.

Reviewer #4

(Remarks to the Author)
I have been asked to serve as a replacement for reviewer 2, with the specific task of addressing the adequacy of the response to the prior reviewer's concerns. Overall, I agree with the other reviewers' appreciation for the technological achievement of the COMBINE approach and think this is a good fit for Nature Communications. However, I have technical concerns regarding the focused CRISPRi screen that should be addressed prior to publication. Furthermore, this experiment and the ZIM3 comparison experiments raise the issue that most of the individual sgRNAs tested to demonstrate the superiority of the L3MBTL3 fusion are performed on sgRNAs designed by CRISPRa rules. This was presumably done to enable studies on dual directional perturbations and increase dynamic range, but in the context of direct CRISPRi experiments serves to over-emphasize the improvement and raises the concern that L3MBTL3 acts through increasing the spread of silencing.
1. The added discussion adequately addresses the concern regarding the limitations of the contexts tested for the COMBINE screens.
2. In response to concerns regarding the validation performed in a small set of genes, the authors have conducted a focused CRISPRi screen to show improvements in functional consequences of knockdown of essential genes. While this is conceptually a good way to address this question, there are a few concerns with the analysis and data.
a. The growth phenotypes are expressed as log2 enrichment. While this is a standard method for expressing screen phenotypes, it is susceptible to confounders when comparing screens across conditions: differences in the growth rate of the derived cell lines, handling of cells at bottlenecks such as sorting, and coverage of the library throughout the experiment (especially for a drop-out screen where essential genes have non-linear phenotypes once those cells have been fully depleted) can all produce a different slope. One method to somewhat correct for this would be to normalize log2 enrichment by the number of doublings for each experiment.
b. It is surprising that the negative controls are so correlated, and overall if the L3MBTL3 fusion is working as shown in the CD81 experiments I would expect a much less monotonic relationship between the KRAB and KRAB+L3MBTL3 conditions.

For one, many of the CRISPRa sgRNAs should give little repression in the KRAB-only condition. For another, the relationship between knockdown and growth phenotype is not linear for many genes, so even a monotonic increase in knockdown would not give the result seen here. I would expect plots resembling PMID: 36576240 Figure 1C or 33838111 Figure 4D. This makes me concerned that there was sample cross-contamination in library preparation that leads to correlated negative controls and phenotypes.
c. As this is examining the effect of L3MBTL3 on CRISPRi efficacy, data should be presented on an individual sgRNA level instead of or in addition to the gene-level plot. To enhance clarity, negatived controls could be centered at 0.
d. I see several potential approaches to further address this, including conducting an additional replicate of each arm using sample prep reagents not previously used for this same library, demonstration of improved endogenous knockdown with RT-qPCR for sgRNAs demonstrating differential effects in the screen, or conducting small-scale Perturb-seq-style experiments to quantify knockdown and transcriptional phenotypes simultaneously.
3. I agree that mechanistic studies are largely outside the scope as presented and for the journal, and the discussion adequately notes this possibility of non-targeted mechanisms. However, the vast improvement seen at upstream loci suggests there could be an increase in spreading of heterochromatin, which is important both for design of KRAB+L3MBTL3 sgRNAs and for understanding the risk of neighbor gene knockdown. H3K9me3 ChIP-seq or similar would address this and also give clues regarding possible mechanisms. An alternative would be a tiling screen of sgRNAs around the TSS of genes to define the change of rules induced by L3MBTL3, but this is outside the scope of the manuscript (unless it is technically easier for the authors than the ChIP-seq suggested above).
4. As noted above, these experiments highlight the improvement in knockdown with L3MBTL3 but are not direct comparisons to sgRNAs that would be used in CRISPRi experiments. I would recommend direct comparison with the CD81/55/151 sgRNAs used in Replogle 2022 (36576240) and discussion of the benefits and risks of increased targeting windows for sgRNAs balanced by increased neighbor effects, etc. to position this work within the existing field.

Reviewer #5

(Remarks to the Author)
This reviewer was tasked with responding to the author's comments made to Reviewer #2.

The authors supply additional experiments, primarily in Figure 5, to address a majority of the concerns (besides mechanistic work).

1) Here, my understanding is that Reviewer #2 was questioning why CD81 was used for activation study when the gene is already highly expressed. It is likely results are biased toward repression because of this, though I do not see major emphasis on activation of CD81 in the current manuscript.

2) The authors have provided additional data in Figure 5 in response to the review. In particular, Figure 5g targets additional genes with the KRAB-L3MBTL3 system. How many of the 57 genes (or 570 guides) are enhanced by L3MBTL3 addition? Why are the designed guides utilized named "CRISPRa"? An alternate terminology would be better served here, such as "upstream".

Figure 5h, i are needlessly dense. Combinations containing the NT guides may be better left in the supplement.

3) Mechanistic study may be considered outside of the scope.

4) The authors provide evidence that L3MBTL3 enhances repression of commonly used KRAB variants. It appears that sgRNAs that perform poorly in the KRAB only system were selected.

Version 1:

Reviewer comments:

Reviewer #4

(Remarks to the Author)
The authors have adequately addressed all of my comments. The tiling screens and comparisons to published sgRNAs nicely contextualize the improvements of the KRAB+L3MBTL3 combination. I have no further reservations prior to publication.

Reviewer #5

(Remarks to the Author)
The authors have addressed my comments/concerns.

Open Access This Peer Review File is licensed under a Creative Commons Attribution 4.0 International License, which permits use, sharing, adaptation, distribution and reproduction in any medium or format, as long as you give appropriate credit to the original author(s) and the source, provide a link to the Creative Commons license, and indicate if changes were made.
In cases where reviewers are anonymous, credit should be given to 'Anonymous Referee' and the source.
The images or other third party material in this Peer Review File are included in the article's Creative Commons license, unless indicated otherwise in a credit line to the material. If material is not included in the article's Creative Commons license and your intended use is not permitted by statutory regulation or exceeds the permitted use, you will need to obtain permission directly from the copyright holder.
To view a copy of this license, visit https://creativecommons.org/licenses/by/4.0/

Response to Reviewers :
We thank the reviewers for their constructive comments and have revised the manuscript according to their suggestions. We provide a detailed point-by-point response to each of the reviewers' concerns below (our responses are in blue text):

REVIEWER COMMENTS
Reviewer #3 (Remarks to the Author):

Moon et al. present COMBINE, a platform for high-throughput screening of domain effector pairs to assess their ability to modulate transcription. The authors note that most existing screening strategies focus on single domains, limiting our understanding of how domains interact when combined. By constructing two libraries with varying effector lengths and implementing differential sequencing strategies, they profile more than 50,000 effector-pair combinations in human cells.

Reviewer #1 raised several valuable points that I agree are important to address. It is worth noting that I have not seen pooled epigenetic effector-screening data calculated in this manner, but this study is also unique in its search for combinatorial effects. The authors responded to reviewer #1's feedback effectively in their response and incorporated clarifying text within the discussion. Thus, I support its publication.

> We thank the reviewer for carefully reviewing the preceding revision process and for their supportive assessment of our manuscript.

Reviewer #4 (Remarks to the Author):

I have been asked to serve as a replacement for reviewer 2, with the specific task of addressing the adequacy of the response to the prior reviewer's concerns. Overall, I agree with the other reviewers' appreciation for the technological achievement of the COMBINE approach and think this is a good fit for Nature Communications. However, I have technical concerns regarding the focused CRISPRi screen that should be addressed prior to publication. Furthermore, this experiment and the ZIM3 comparison experiments raise the issue that most of the individual sgRNAs tested to demonstrate the superiority of the L3MBTL3 fusion are performed on sgRNAs designed by CRISPRa rules. This was presumably done to enable studies on dual directional perturbations and increase dynamic range, but in the context of direct CRISPRi experiments serves to over-emphasize the improvement and raises the concern that L3MBTL3 acts through increasing the spread of silencing.

> We appreciate the reviewer's effort in taking the time to review the preceding revision process and their expert suggestions for improving the manuscript. We acknowledge the concern that using sgRNAs designed by CRISPRa rules may over-emphasize the improvement

seen with L3MBTL3. To address this, we have performed additional experiments and edited the manuscript as described in the specific responses below.

1. The added discussion adequately addresses the concern regarding the limitations of the contexts tested for the COMBINE screens.

> We thank the reviewer for this assessment.

2. In response to concerns regarding the validation performed in a small set of genes, the authors have conducted a focused CRISPRi screen to show improvements in functional consequences of knockdown of essential genes. While this is conceptually a good way to address this question, there are a few concerns with the analysis and data.

a. The growth phenotypes are expressed as log2 enrichment. While this is a standard method for expressing screen phenotypes, it is susceptible to confounders when comparing screens across conditions: differences in the growth rate of the derived cell lines, handling of cells at bottlenecks such as sorting, and coverage of the library throughout the experiment (especially for a drop-out screen where essential genes have non-linear phenotypes once those cells have been fully depleted) can all produce a different slope. One method to somewhat correct for this would be to normalize log2 enrichment by the number of doublings for each experiment.

> We appreciate the reviewer's expertise regarding potential confounders in essential gene growth screens. We acknowledge that normalizing by the number of doublings would be ideal; unfortunately, we did not record the exact number of cells during passaging. However, we sorted and maintained at least 1000x coverage relative to the guide RNA library at every step, and given the relatively small size of our library (1340 guides), a single T75 flask was sufficient to ensure high coverage (minimum of 1000x) throughout. Regarding potential differences in growth rate between derived cell lines, the KRAB-L3MBTL3 cell line showed a similar growth rate to its parental cell line (Supplementary Figure 21c), suggesting this is unlikely to be a significant confounder.

b. It is surprising that the negative controls are so correlated, and overall if the L3MBTL3 fusion is working as shown in the CD81 experiments I would expect a much less monotonic relationship between the KRAB and KRAB+L3MBTL3 conditions. For one, many of the CRISPRa sgRNAs should give little repression in the KRAB-only condition. For another, the relationship between knockdown and growth phenotype is not linear for many genes, so even a monotonic increase in knockdown would not give the result seen here. I would expect plots resembling PMID: 36576240 Figure 1C or 33838111 Figure 4D. This makes me concerned that there was sample cross-contamination in library preparation that leads to correlated negative controls and phenotypes.

> We appreciate the reviewer's precise comment regarding the correlation between negative controls, which differs from PMID: 36576240 Figure 1C and 33838111 Figure 4D. We

note a key difference in experimental procedure: while those studies used cells post-infection as T0 for each replicate, we used the plasmid pool as T0. This could account for the observed correlation, as any shift in lentiviral library representation compared to the plasmid library would introduce correlated signal across replicates when normalizing against the plasmid pool, independent of growth effects. While this is not ideal, we performed additional experiments the reviewer suggested to examine the reliability of our essential gene screen results (see response to Comment 2d).

c. As this is examining the effect of L3MBTL3 on CRISPRi efficacy, data should be presented on an individual sgRNA level instead of or in addition to the gene-level plot. To enhance clarity, negatived controls could be centered at 0.

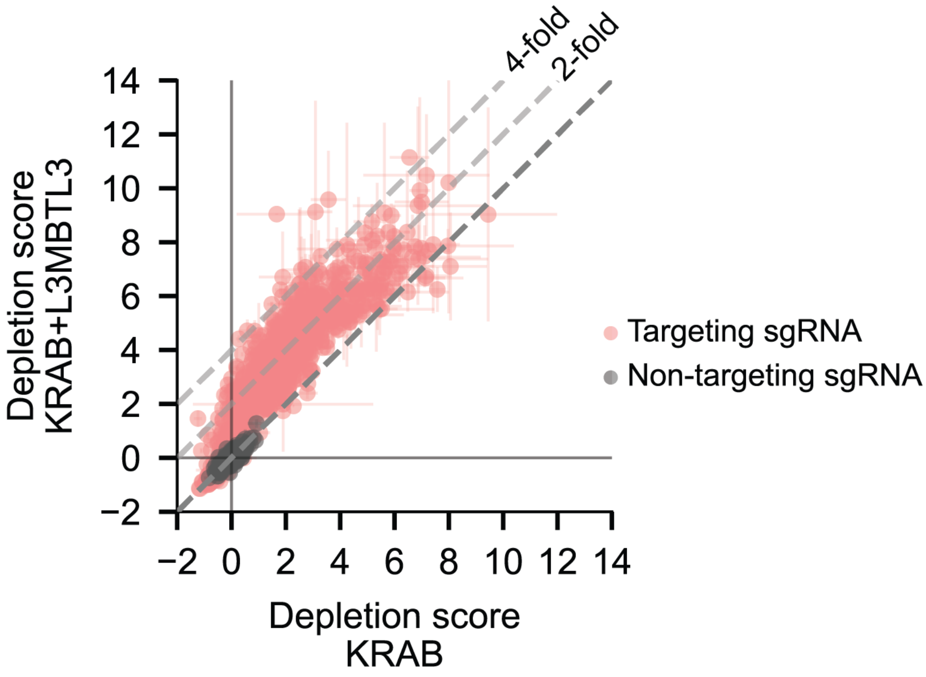

> We added a scatter plot at the individual sgRNA level to Figure 5. All data are normalized such that non-targeting controls are centered at 0. Based on our revised conclusions from the sgRNA tiling screen (see response to Comment 3), we now present the data without distinguishing between TSS-downstream and upstream sgRNAs.

d. I see several potential approaches to further address this, including conducting an additional replicate of each arm using sample prep reagents not previously used for this same library, demonstration of improved endogenous knockdown with RT-qPCR for sgRNAs demonstrating differential effects in the screen, or conducting small-scale Perturb-seq-style experiments to quantify knockdown and transcriptional phenotypes simultaneously.

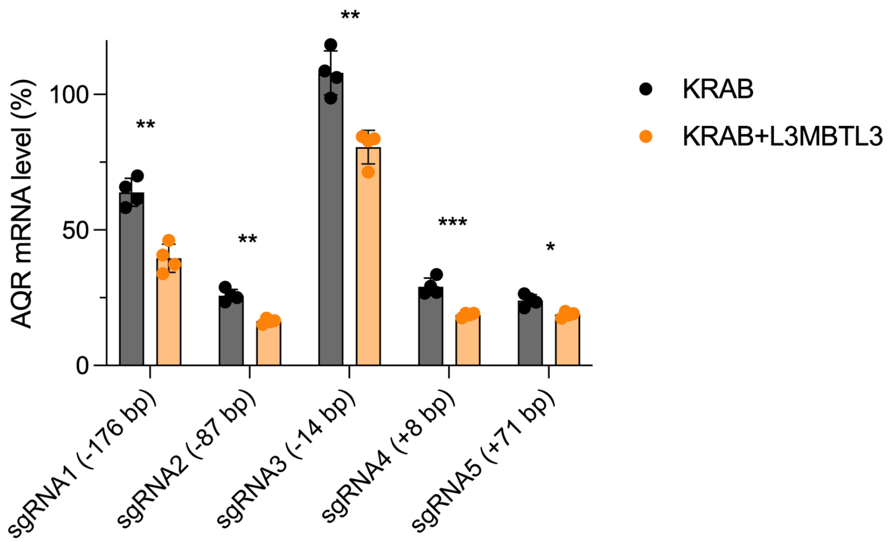

> We conducted RT-qPCR validation for 5 sgRNAs targeting the AQR gene, one of the essential genes included in the screen. All 5 sgRNAs, targeting both upstream and downstream of the TSS, showed enhanced transcriptional repression with KRAB+L3MBTL3 compared to KRAB alone, consistent with the screen results.

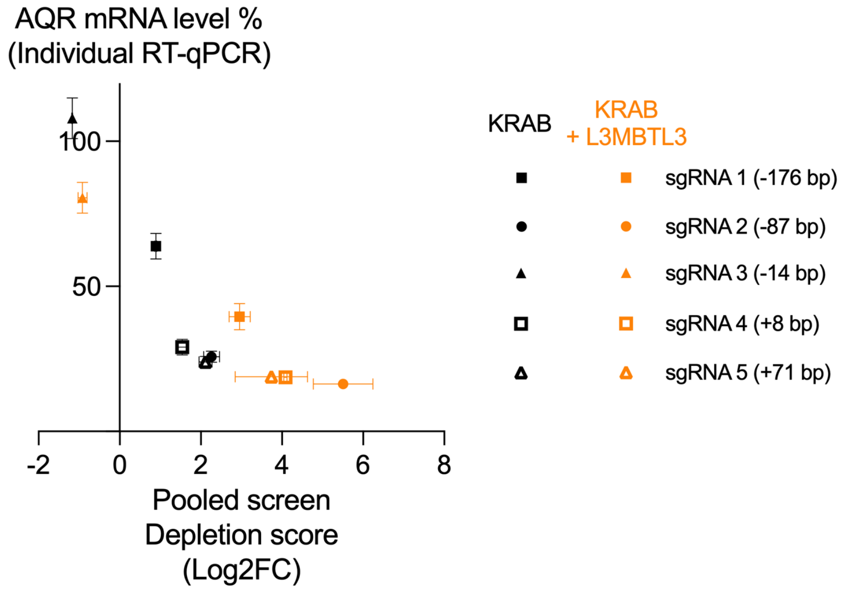

When plotting depletion scores from the pooled screen against AQR mRNA levels measured by RT-qPCR, we observed concordance between screen results and individual validations. For example, sgRNA3, which poorly represses AQR, shows minimal depletion, whereas sgRNAs that strongly repress AQR exhibit higher depletion scores. These validation results are included in Supplementary Figure 23.

In conclusion, we acknowledge that the correlation between negative controls is not ideal, likely due to using a single plasmid pool as the initial timepoint (T0). However, individual RT-qPCR validation experiments confirm the reliability of our screen results and support the

core conclusion: the KRAB+L3MBTL3 combination enhances transcriptional repression compared to KRAB alone across diverse target genes.

3. I agree that mechanistic studies are largely outside the scope as presented and for the journal, and the discussion adequately notes this possibility of non-targeted mechanisms. However, the vast improvement seen at upstream loci suggests there could be an increase in spreading of heterochromatin, which is important both for design of KRAB+L3MBTL3 sgRNAs and for understanding the risk of neighbor gene knockdown. H3K9me3 ChIP-seq or similar would address this and also give clues regarding possible mechanisms. An alternative would be a tiling screen of sgRNAs around the TSS of genes to define the change of rules induced by L3MBTL3, but this is outside the scope of the manuscript (unless it is technically easier for the authors than the ChIP-seq suggested above).

CD81 sgRNA tiling screen

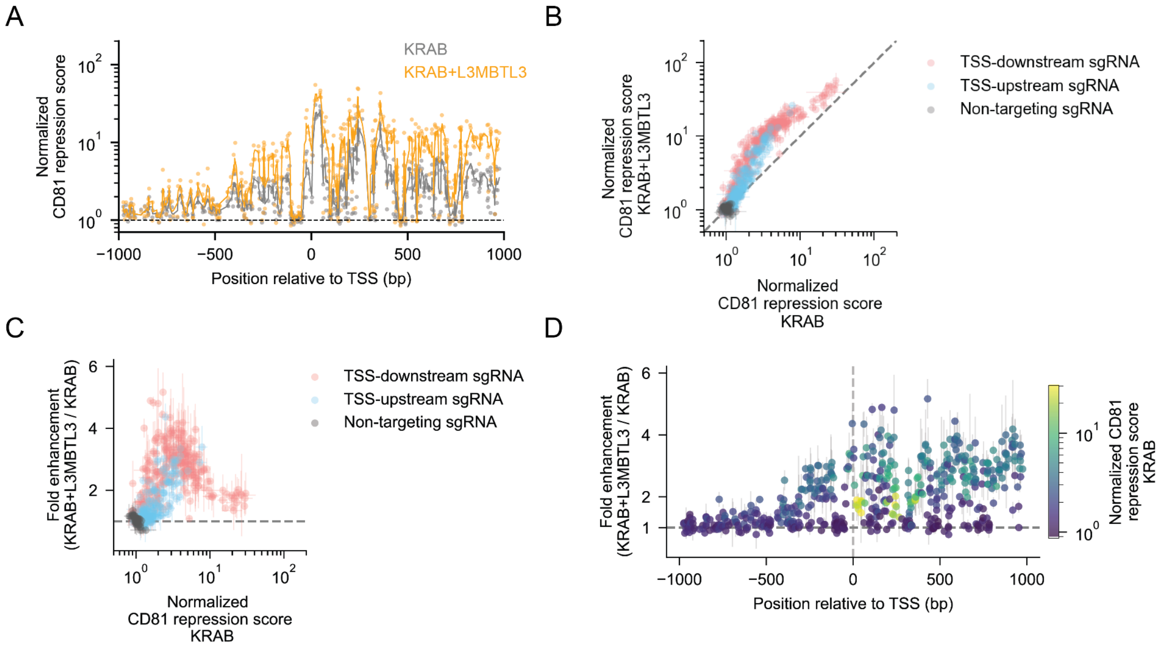

> Thank you for this suggestion. We performed tiling screens of sgRNAs spanning ±1000 bp around the TSS of three genes (CD55, CD81, CD151). CD55 and CD151 results are presented in Supplementary Figure 23.

Analysis of the tiling screen suggests that the fold-change improvement (KRAB+L3MBTL3 vs KRAB) is not preferentially greater for upstream-targeting sgRNAs. For example, in the panel B&C, TSS-downstream sgRNAs with moderate potency show greater fold enhancement compared to upstream sgRNAs of similar potency. We also observed that for highly potent sgRNAs, the fold change approaches y=x (panel B), likely because these sgRNAs already achieve substantial knockdown with KRAB alone, leaving limited room for further improvement (panel C). This is consistent with new individual validation experiments using

sgRNAs from Replogle 2022 (see response to Comment 4). However, we also discovered sgRNAs (panel D) that show substantial enhancement with the KRAB+L3MBTL3 combination, which could be useful when target site constraints exist (e.g., allele-specific targeting).

While the tiling screen does not directly address heterochromatin spreading (which would require H3K9me3 ChIP-seq), it demonstrates that the improvement with KRAB+L3MBTL3 is not preferentially greater at upstream loci. We revised our conclusions accordingly and incorporated these results into the main text (lines: 421-429), Figure 5f,g, and Supplementary Figure 23.

4. As noted above, these experiments highlight the improvement in knockdown with L3MBTL3 but are not direct comparisons to sgRNAs that would be used in CRISPRi experiments. I would recommend direct comparison with the CD81/55/151 sgRNAs used in Replogle 2022 (36576240) and discussion of the benefits and risks of increased targeting windows for sgRNAs balanced by increased neighbor effects, etc. to position this work within the existing field.

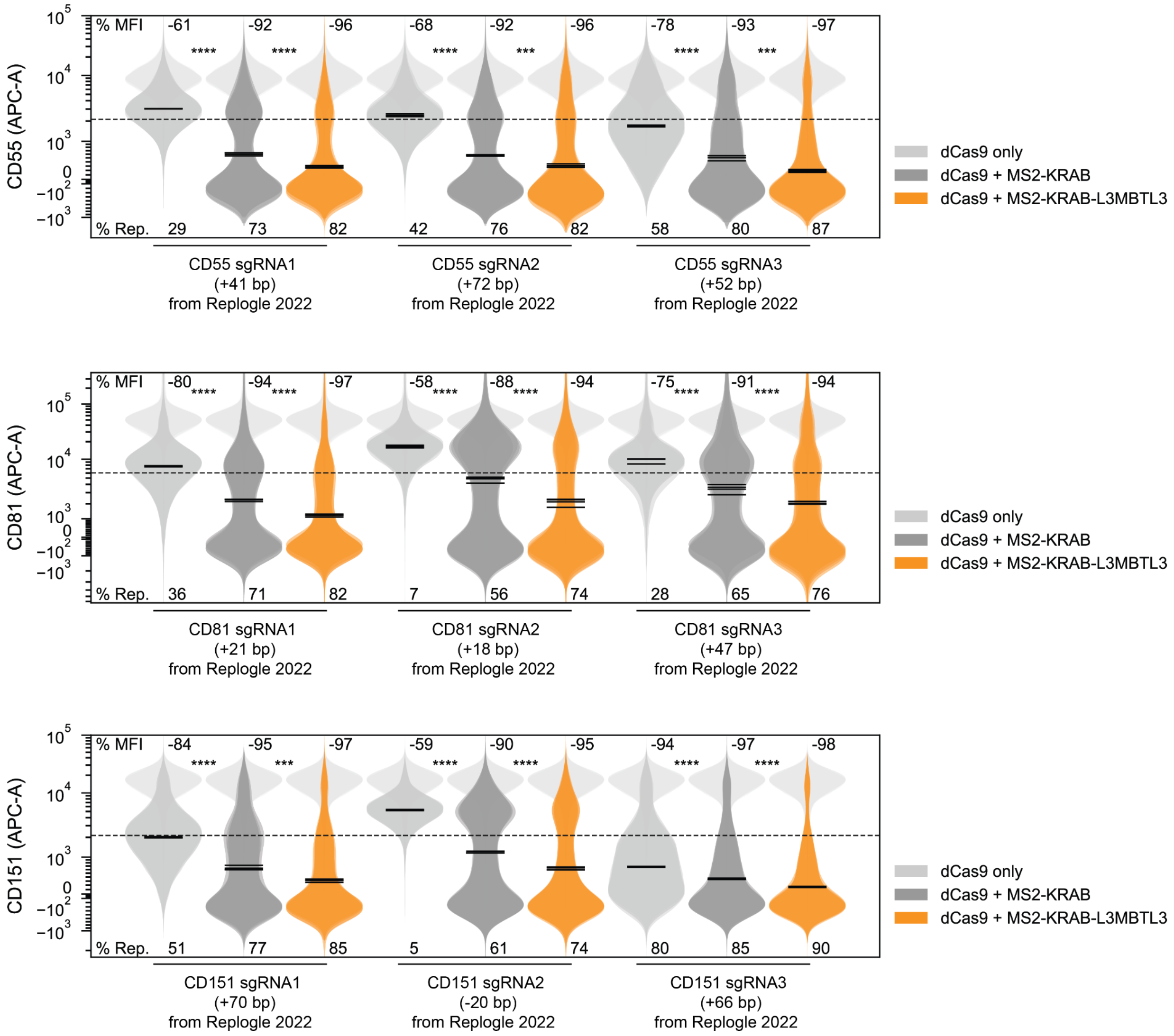

> Thank you for the suggestion. We performed CRISPRi experiments on CD55, CD81, and CD151 with the top 3 sgRNAs from Replogle 2022 (PMID: 36576240) paired with three effector conditions: dCas9 only (physical blockade of RNA polymerase II), KRAB, and KRAB+L3MBTL3. These sgRNAs achieved substantial knockdown even with dCas9 only or KRAB alone, leaving limited room for further improvement by L3MBTL3. This is consistent with the sgRNA tiling screen results, where highly potent sgRNAs show minimal fold changes.

Throughout the manuscript, we clarified that while strong sgRNAs may reach a ceiling, KRAB+L3MBTL3 can substantially enhance repression for subsets of sgRNAs including weaker sgRNAs (lines: 412-421 and 424-427). We further discuss the benefits and risks of increased potency of KRAB+L3MBTL3 in the Discussion section (lines: 574-577).

Reviewer #5 (Remarks to the Author):

This reviewer was tasked with responding to the author's comments made to Reviewer #2.

The authors supply additional experiments, primarily in Figure 5, to address a majority of the concerns (besides mechanistic work).

> We appreciate the reviewer's effort in taking the time to review the preceding revision process and their suggestions for improving the manuscript.

1) Here, my understanding is that Reviewer #2 was questioning why CD81 was used for activation study when the gene is already highly expressed. It is likely results are biased toward repression because of this, though I do not see major emphasis on activation of CD81 in the current manuscript.

> We thank the reviewer for this point. As noted, we do not emphasize CD81 activation in the current manuscript. We also acknowledge this consideration in the Discussion (lines 589–592), where we recommend performing screens tailored to specific contexts of interest.

2) The authors have provided additional data in Figure 5 in response to the review. In particular, Figure 5g targets additional genes with the KRAB-L3MBTL3 system. How many of the 57 genes (or 570 guides) are enhanced by L3MBTL3 addition? Why are the designed guides utilized named "CRISPRa"? An alternate terminology would be better served here, such as "upstream". Figure 5h, i are needlessly dense. Combinations containing the NT guides may be better left in the supplement.

> Thank you for the suggestion. We have changed the terminology from "CRISPRa" to "TSS-upstream" and "CRISPRi" to "TSS-downstream" throughout the manuscript.

Regarding the enhancement by L3MBTL3 addition: at the guide level, 918/1140 (80.5%) targeting guides exceeded the enhancement threshold (difference in depletion score between KRAB+L3MBTL3 and KRAB > 2 SD of non-targeting controls), providing ~1.8X greater effect sizes overall. At the gene level, 54/57 genes (94.7%) showed significant enhancement in depletion (Wilcoxon signed-rank test, FDR < 0.05). These results are now incorporated into the main text (lines: 442-447) and Supplementary Table 8.

3) Mechanistic study may be considered outside of the scope.

> We thank the reviewer for this consideration.

4) The authors provide evidence that L3MBTL3 enhances repression of commonly used KRAB variants. It appears that sgRNAs that perform poorly in the KRAB only system were selected.

> We would like to mention that all the CD81 sgRNAs in Figure 5 are the exact sgRNAs that were used for high-throughput screening of combinatorial epigenetic effectors.

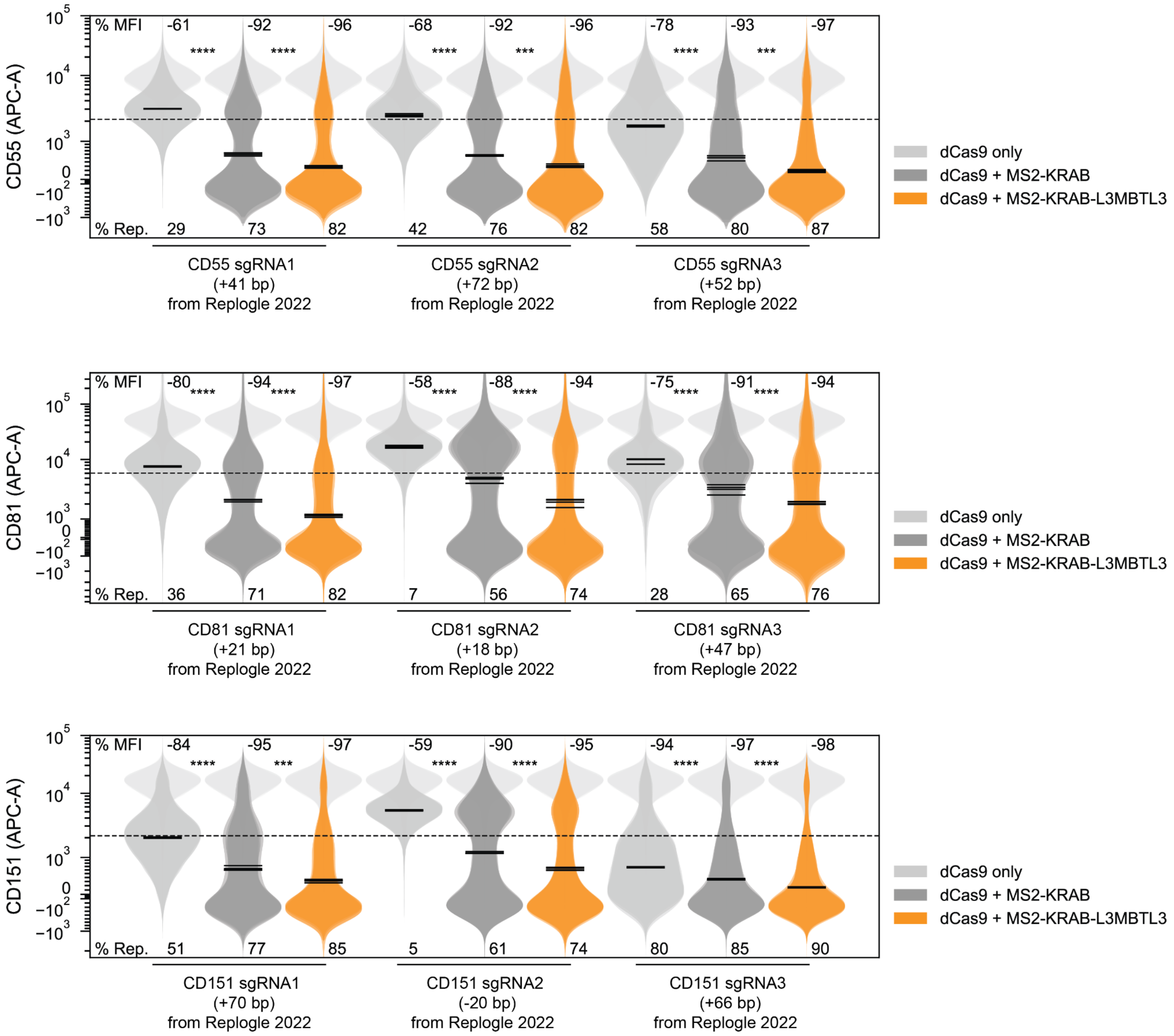

However, we also acknowledge that strong sgRNAs can already achieve sufficient repression with KRAB alone or even dCas9 only, leaving limited room for further improvement. During this revision round, we added a comparison experiment using strong sgRNAs from Replogle 2022 (PMID: 36576240). These results are incorporated into the main text (lines: 412-421) and Supplementary Figure 22.

Moreover, the new sgRNA tiling screen data (Figure 5f,g and Supplementary Figure 23) demonstrates this sgRNA-dependent differential enhancement. Throughout the manuscript, we clarified that while strong sgRNAs may reach a ceiling, KRAB+L3MBTL3 can substantially enhance repression for subsets of sgRNAs including weaker sgRNAs (lines: 412-421 and 424-427).

CD81 sgRNA tiling screen

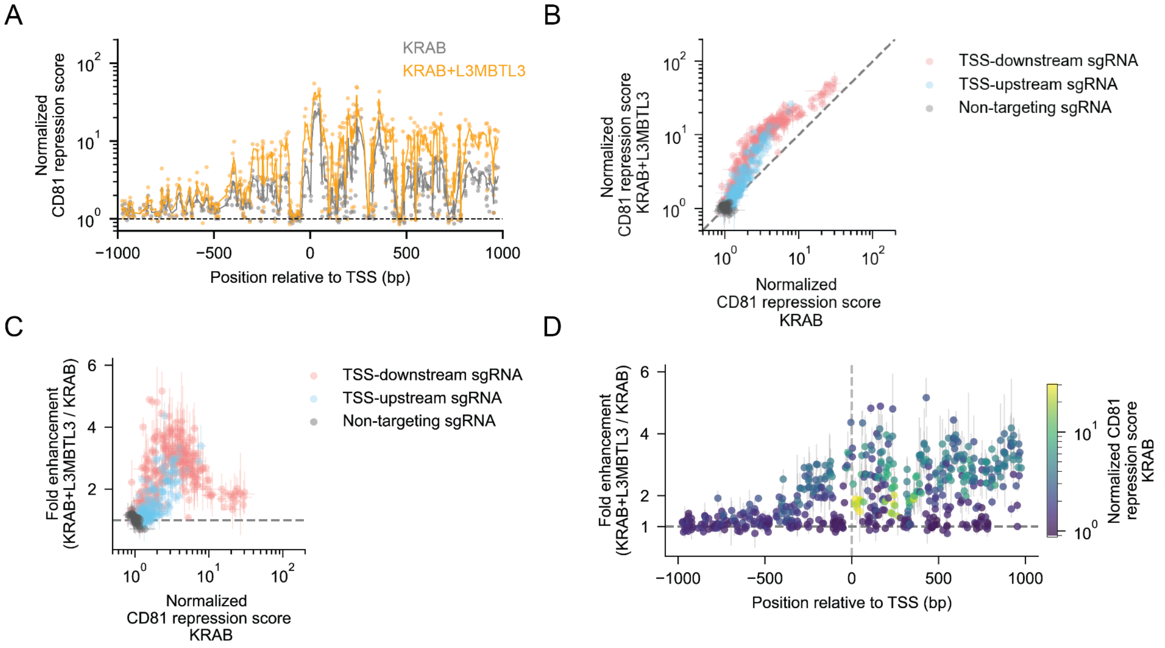

Supplement: Supplementary file 15 — Transparent Peer Review file [file 41467_2026_72227_MOESM15_ESM.pdf]
